# Mortality in sepsis and septic shock in Europe, North America and Australia between 2009 and 2019— results from a systematic review and meta-analysis

**DOI:** 10.1186/s13054-020-02950-2

**Published:** 2020-05-19

**Authors:** Michael Bauer, Herwig Gerlach, Tobias Vogelmann, Franziska Preissing, Julia Stiefel, Daniel Adam

**Affiliations:** 1 Universitätsklinikum Jena, Klinik für Anästhesiologie und Intensivmedizin, Am Klinikum 1, 07747 Jena, Germany; 2 Vivantes Klinikum Neukölln, Rudower Straße 48, 12351 Berlin, Germany; 3 LinkCare GmbH, Kyffhäuserstr. 64, 70469 Stuttgart, Germany; 4 CytoSorbents Europe GmbH, Müggelseedamm 131, 12587 Berlin, Germany

**Keywords:** Sepsis, Septic shock, Mortality, Trend, SOFA, Meta-analysis

## Abstract

**Background:**

Sepsis and septic shock remain drivers for mortality in critically ill patients. The heterogeneity of the syndrome hinders the generation of reproducible numbers on mortality risks. Consequently, mortality rates range from 15 to 56%. We aimed to update and extend the existing knowledge from meta-analyses and estimate 30- and 90-day mortality rates for sepsis and septic shock separately, stratify rates by region and study type and assess mortality rates across different sequential organ failure assessment (SOFA) scores.

**Methods:**

We performed a systematic review of articles published in PubMed or in the Cochrane Database, between 2009 and 2019 in English language including interventional and observational studies. A meta-analysis of pooled 28/30- and 90-day mortality rated separately for sepsis and septic shock was done using a random-effects model. Time trends were assessed via Joinpoint methodology and for the assessment of mortality rate over different SOFA scores, and linear regression was applied.

**Results:**

Four thousand five hundred records were identified. After title/abstract screening, 783 articles were assessed in full text for eligibility. Of those, 170 studies were included. Average 30-day septic shock mortality was 34.7% (95% CI 32.6–36.9%), and 90-day septic shock mortality was 38.5% (95% CI 35.4–41.5%). Average 30-day sepsis mortality was 24.4% (95% CI 21.5–27.2%), and 90-day sepsis mortality was 32.2% (95% CI 27.0–37.5%). Estimated mortality rates from RCTs were below prospective and retrospective cohort studies. Rates varied between regions, with 30-day septic shock mortality being 33.7% (95% CI 31.5–35.9) in North America, 32.5% (95% CI 31.7–33.3) in Europe and 26.4% (95% CI 18.1–34.6) in Australia. A statistically significant decrease of 30-day septic shock mortality rate was found between 2009 and 2011, but not after 2011. Per 1-point increase of the average SOFA score, average mortality increased by 1.8–3.3%.

**Conclusion:**

Trends of lower sepsis and continuous septic shock mortality rates over time and regional disparities indicate a remaining unmet need for improving sepsis management. Further research is needed to investigate how trends in the burden of disease influence mortality rates in sepsis and septic shock at 30- and 90-day mortality over time.

## Background

Sepsis and septic shock remain high-risk factors for mortality in critically ill patients [1]. Sepsis, i.e. organ dysfunction due to an inappropriate host response to infection affects approximately 1 out of 3 intensive care unit patients. Worldwide, sepsis is estimated to affect more than 30 million people every year, potentially leading to 6 million deaths [2].

However, the heterogeneity of sepsis and septic shock hinders the generation of reproducible data on mortality risks, with ranges between 15 and 56% reported [1, 3–6]. There is also dissent regarding trends in mortality over time, and while most studies have reported a decline, the annual decrease has been estimated in the range of 0.42 to 3.3% [1, 7–9].

These variations may in part be explained by variations in severity, type of study, geographical region and improvements in the standard of care. Regarding the latter, improved care has been attributed to the launch of the international guidelines of the Surviving Sepsis Campaign [10, 11], although this has created substantial controversy and the ‘1 hour bundle’ has been put on hold in the United States (US) recently [12]. One significant argument to stop the bundle, specifically for the US, was justified by potential differences between the US and resource-limited countries, albeit that systematic data for regional differences are lacking.

Despite the alleged importance of the severity of the disease in determining the mortality rate, few studies have discriminated accordingly. A retrospective study using a national critical care database reported an overall hospital sepsis mortality rate of 31.8% versus a 55.5% for septic shock patients [6], while another retrospective cohort study using health record data reported 15% in hospital mortality for sepsis and septic shock [1].

So far, comparisons between mortality rates for sepsis and septic shock are missing, as are comparisons between 30- and 90-day mortality, and an analysis to explore any impact of geographical regions or turning points in mortality trends over time.

Accordingly, we investigated mortality trends in patients with sepsis and septic shock from 2009 to 2019 including interventional, observational, prospective and retrospective studies to get a broad understanding of published mortality rates. This enabled us to stratify results by study types and geographic region (Europe, North America, Australia) as secondary objectives. Additionally, mortality rates were estimated for changing SOFA score, to take the severity of disease into account.

## Methods

### Eligibility criteria

A systematic literature search following the Preferred Reporting Items for Systematic Reviews and Meta-Analyses (PRISMA) statement [13] was conducted from February 14, 2019, to March 29, 2019. A sensitive strategy was used to search PubMed for randomized trials and prospective as well as retrospective cohort studies, and meta-analyses enrolling patients with sepsis or septic shock. The Cochrane Library was searched for systematic reviews. We used the following terms plus combinations thereof to identify studies in PubMed: sepsis/septic shock, length of stay, mortality and multi-organ failure. The full list of search terms is provided online (Additional file 1: Tables S1-S2).

Studies were included if they (i) enrolled adult patients with suspected or confirmed sepsis, severe sepsis, or septic shock according to the definition by Bone et al. [14] or sepsis in the Sepsis-3 definition [15]; (ii) reported overall 30- ± 2 days) or 90-day mortality; and (iii) were published between 2009 and 2019. Studies were restricted to English publications, which were (iv) conducted in Europe, North America and Australia (incl. New Zealand), to guarantee a similar standard of care. Multi-country studies that were conducted in the mentioned regions and for example an Asian or South American country were included as well. We decided against including studies that were solely performed in Asia, Africa, or South America because previous studies showed higher mortality rates in developing regions than in developed regions due to non-comparable healthcare systems [16]. (v) Studies with numbers of patients ≤ 20 and population-based studies that addressed specific socioeconomic strata were excluded.

### Study selection, data-collection process and data items

Based on the title and abstract screening, studies that met explicit predefined exclusion criteria, namely assessment in animals or in vitro only, results not related to sepsis, none relevant outcomes, or paediatric only studies, were excluded. In the second step, the remaining manuscripts were assessed for full-text reading by two independent reviewers. In cases of disagreement among the reviewers, a third reviewer assessed the manuscript and a decision for inclusion was reached by consensus. To avoid bias from duplicate data, patient cohorts reported in multiple studies were only considered only once by the reviewers.

Three independent investigators (T.V., J.S. and D.A.) extracted data from the included studies into a pretested Microsoft Office Excel spreadsheet, which was designed according to the checklist of the *data extraction for complex meta-analysis (DECiMAL)* guide [17].

Utilizing the data extraction spreadsheet, the following data were recorded from each trial: (1) study characteristics (including study type, geographical location, timing and number of sites and enrolled participants); (2) characteristics of trial participants including sepsis type, age (mean or median), stage and severity of disease in SOFA score (mean or median); (3) type of outcome measure (including length of follow-up, 30-day mortality, 90-day mortality). As 28-day mortality was commonly reported, we included 30-day (± 2 days) mortality. A reference list of included studies is provided online (Additional file 2). An overview of all included studies that shows which study was used for which end point (septic shock/sepsis 30-/90-day mortality) in the meta-analysis is provided in the additional files (Additional file 3: Tables S1-S4).

### Assessment of risk of bias

Risk of bias of the studies that were eligible for meta-analysis were performed using ‘The Cochrane Collaboration’s Tool RoB 2’ for randomized trials [18] and with ‘ROBINS-I’ used for observational studies [19], for sepsis and septic shock, 30 and 90 days mortality each. Risk of bias for meta-analyses and systematic reviews was assumed as ‘low’.

### Statistical analysis

Univariate random-effects models were used to calculate pooled point mortality estimates for all outcomes (sepsis, septic shock, 30-day and 90-day mortality each) along with 95% confidence intervals (95% CIs). Random-effects models were used to address the expected heterogeneity between the included studies. To stratify data by region, each study was assigned to one of the regions Europe, North America and Australia (incl. New Zealand), according to the location of study sites. Studies conducted in more than one geographical region were excluded from these particular analyses but are part of the overall meta-analysis. For stratification by study type, studies were assigned to ‘RCT’, ‘prospective cohort study’, ‘retrospective cohort study’, ‘meta-analysis’ or ‘other study type’, whereas the category ‘meta-analysis’ and ‘other study type’ (including a mix of several study types, e.g. mixed methods between observational studies and RCTs) were not reported in this paper. We calculated separate random effects pooled mortality per region and type of study. Heterogeneity across studies and consistency were evaluated through Cochran’s Q and the *I*^2^ statistics, respectively. Linear regressions were used to calculate the change of average 30-day/90-day septic shock/sepsis mortality with increasing SOFA scores in studies. *R*^2^ was used to determine the strengths of correlation.

Trends in 30-day/90-day septic shock/sepsis mortality were assessed between 2009 and 2019. To investigate whether there was a trend in mortality over time, pooled mortality was estimated separately per year using random effects models. Trends for mortality were then analysed using the Joinpoint Trend Analysis 4.7.0.0 (National Cancer Institute). The Joinpoint Trend Analysis uses permutation testing among other statistical methods to optimize standard errors and determine the number of times a trend changes. A Joinpoint year is defined as the year in which a statistically significant trend change was found [20]. Data were analysed by using SAS® software version 9.3 (SAS Institute Inc., Cary, NC, USA), Microsoft® Excel 2016 (Microsoft Corporation, Redmond, WA, USA) and Joinpoint Trend Analysis Software Version 4.7.0.0.

## Results

### Study selection

**Fig. 1:**
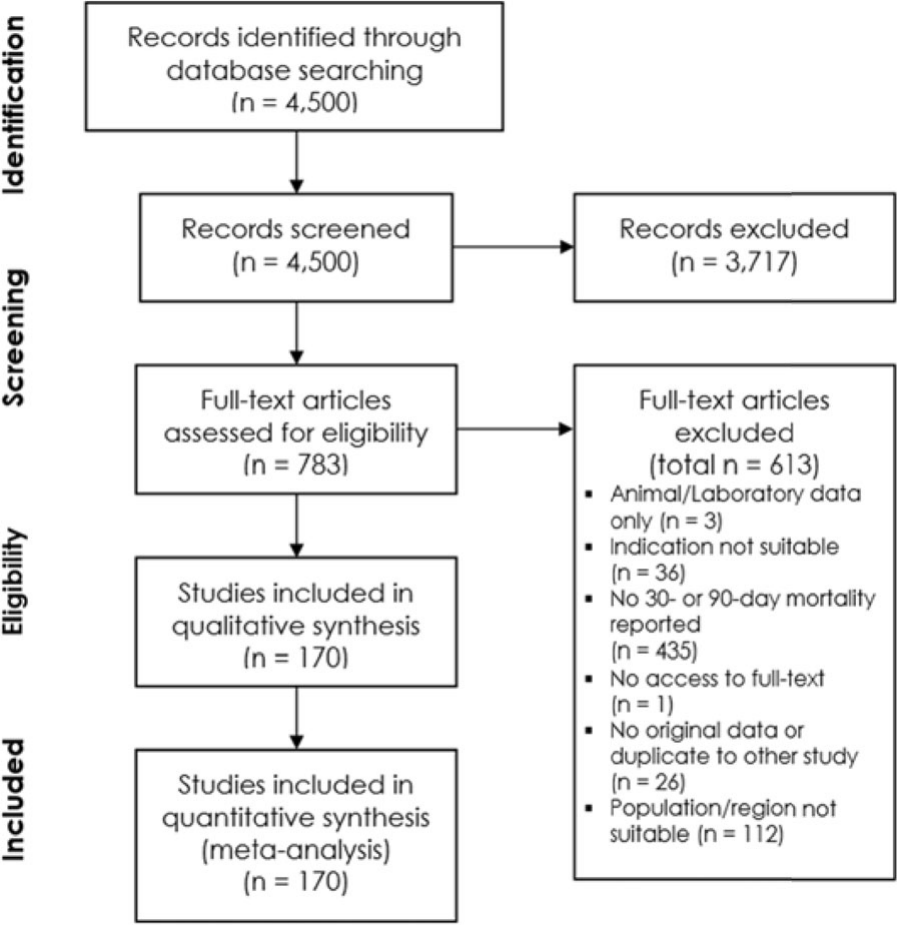
PRISMA flowchart of studies. PRISMA flowchart describing the process of selecting studies eligible for meta-analysis

Of the 4500 records identified in the database search, 3717 records were excluded after title and abstract screening and 783 records were assessed in full-text for eligibility. The full-text screening excluded 613 records. Reasons for exclusion for each study are reported online (Additional file 5: Table S1). One hundred seventy studies were included in the pooled analysis (Fig. 1). Of those, 80 reported data on 30-day (± 2 days) septic shock mortality, 37 on 90-day septic shock mortality, 72 on 30-day sepsis mortality and 25 on 90-day mortality (multiple outcome reporting was possible per study).

### Study characteristics

Included studies represented a total of 371,937 patients with a sample size per study ranging from 21 to 108,939 patients. Median age of patients from all studies was 64 years (quartile range, 61–67), and median SOFA score was 9.5 (quartile range, 8–10). Most of the included studies were prospective cohort studies (*n* = 60), followed by RCTs (*n* = 49), retrospective cohort studies (*n* = 32) and others (*n* = 29).

### Risk of bias assessment

The results of risk of bias assessment of the studies for RCTs, assessed with RoB2 and observational studies, assessed with ROBINS-I for each outcome are presented in the supplementary material (Additional file 3: Tables S1-S4).

### Synthesis of results

#### 30-day septic shock mortality

**Fig. 2:**
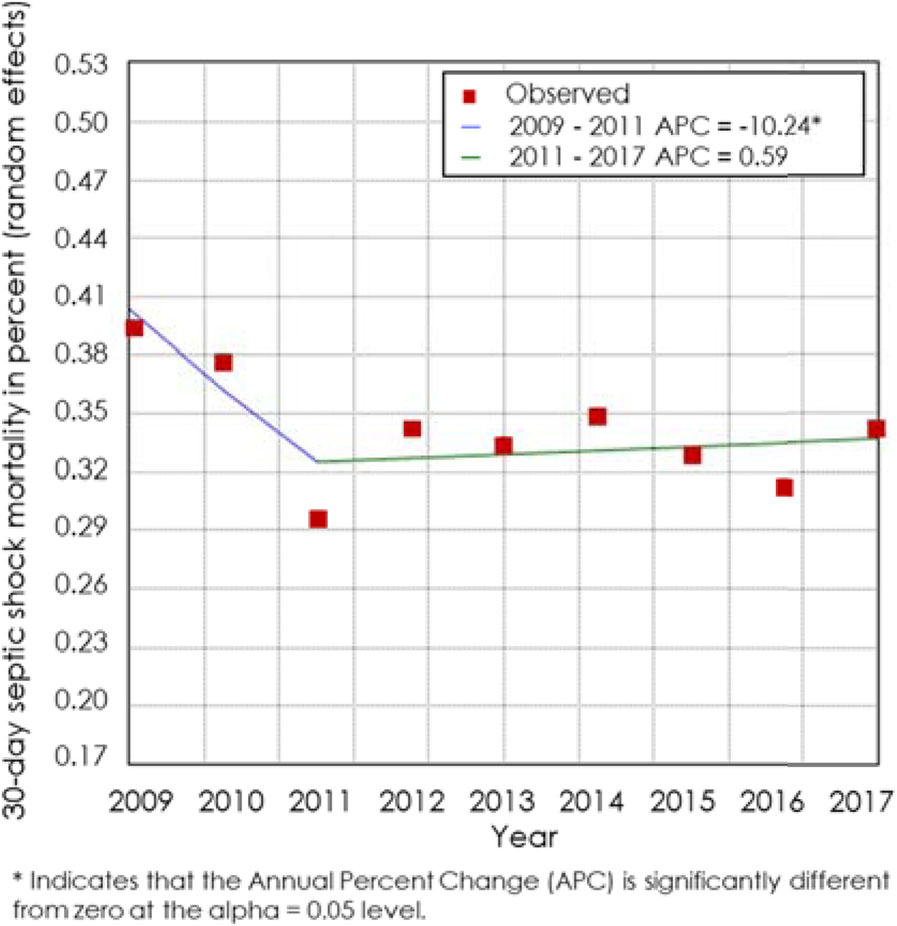
Joinpoint estimation of 30-day septic shock mortality. Results from the estimation of pooled 30-day septic shock mortality rates per year. The Joinpoint analysis shows the time point when trends in mortality rates change. A trend change of 30-day sepsis mortality was found in 2011

Observed 30-day mortality of septic shock patients was 34.73% (95% CI 32.61–36.85%). Mortality rates ranged between 15 and 57% per study. *I*^2^ for heterogeneity of 94.24, indicated high heterogeneity. This analysis covered 56,641 participants from *N* = 80 studies. Mortality rate trends are reported from 2009 to 2018 (Fig. 2). 2011 was identified as Joinpoint year, where a statistically significant trend change of mortality rate was found.

**Fig. 3:**
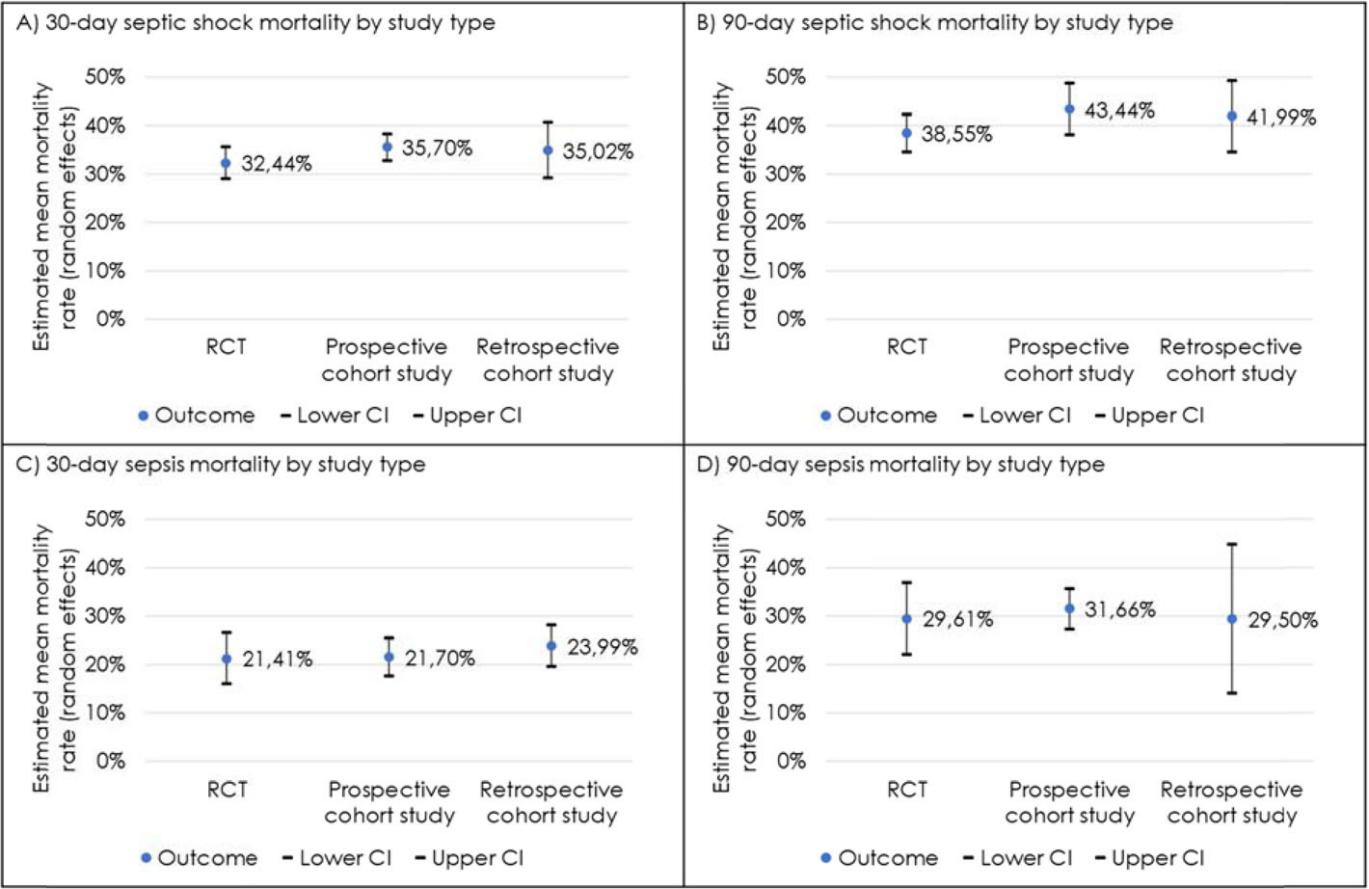
30-day/90-day septic shock/sepsis mortality stratified by study type. Comparison of pooled mortality rates derived from RCTs and prospective and retrospective cohort studies, showing rates and 95% confidence intervals. Mortality rates per study type are reported for **a** 30-day septic shock, **b** 90-day septic shock, **c** 30-day sepsis and **d** 90-day sepsis

RCTs (*N* = 25 studies with 18,312 participants) reported an average 30-day septic shock mortality rate of 32.44% (95% CI 29.19–35.69%), prospective cohort studies (*N* = 26 studies with 6372 participants) of 35.70% (95% CI 32.94–38.46%) and retrospective cohort studies (*N* = 15 studies with 2593 participants) of 35.02% (95% CI 29.28–40.76%) (Fig. 3). The remaining 14 studies were other study types and not reported in this sub analysis. The pooled mortality rate from RCTs was slightly lower than from prospective and retrospective cohort studies.

**Fig. 4:**
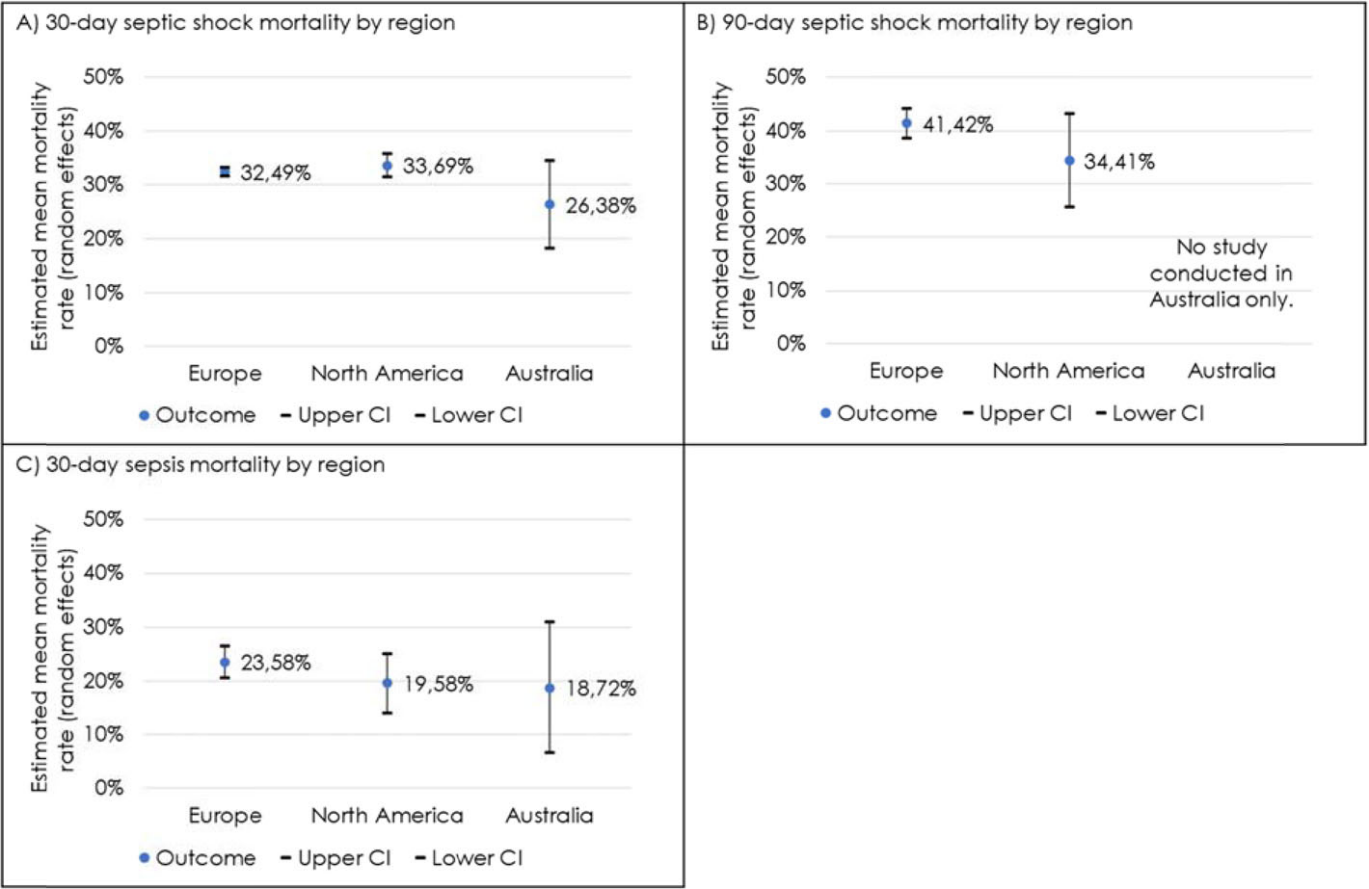
30-day/90-day septic shock/sepsis mortality stratified by geographic region. Comparison of pooled mortality rates per region, showing rates and 95% confidence intervals. Studies were assigned to the regions Europe, North America or Australia based on primary study site. Mortality rates per region are reported for **a** 30-day septic shock, **b** 90-day septic shock, and **c** 30-day sepsis

*N* = 46 studies conducted in Europe with 19,667 participants reported an average 30-day septic shock mortality rate of 32.49% (95% CI 31.70–33.29%), studies conducted in North America (*N* = 12 studies with 2724 participants) of 33.69% (95% CI 31.51–35.86%) and studies conducted in Australia (*N* = 2 studies with 149 participants) of 26.38% (95% CI 18.14–34.63%) (Fig. 4).

#### 90-day septic shock mortality

Observed 90-day mortality of septic shock patients was 38.47% (95% CI 35.44–41.51%). Mortality rates ranged between 18.70 and 61.29%. An *I*^2^ for heterogeneity of 95.55 indicated high heterogeneity. This analysis covered 36,359 participants from *N* = 37 studies. Because of a lack of studies published in 2009 and 2011, Joinpoint analysis was not feasible.

Data from RCTs (*N* = 23 studies with 19,814 participants) report an average 90-day septic shock mortality rate of 38.55% (95% CI 34.68–42.41%), compared to prospective cohort studies (*N* = 8 studies with 4981 participants) of 43.44% (95% CI 38.18–48.69%) and retrospective cohort studies (*N* = 2 studies with 2011 participants) of 41.99% (95% CI 34.63–49.35%) (Fig. 3). The remaining 4 studies were other study types and not reported in this sub analysis.

*N* = 25 studies conducted in Europe with 18,046 participants reported an average 90-day septic shock mortality rate of 41.42% (95% CI 38.73–44.12%), compared to studies conducted in North America (*N* = 3 studies with 1493 participants) of 34.41% (95% CI 25.66–43.16%), no studies conducted in Australia could be included (Fig. 4).

#### 30-day sepsis mortality

**Fig. 5:**
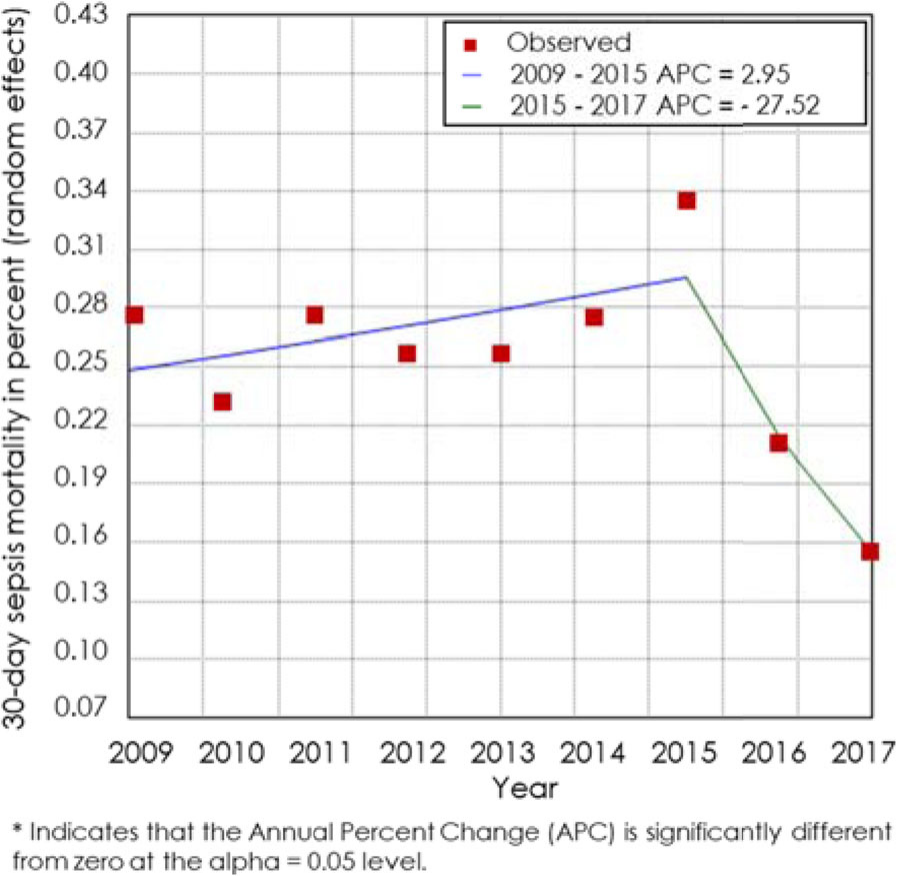
Joinpoint estimation of 30-day sepsis mortality. Results from the estimation of pooled 30-day sepsis mortality rates per year. Joinpoint analysis shows the time point when trends in mortality rates change. One trend change of 30-day sepsis mortality was found in 2015

Observed 30-day mortality rates of sepsis patients were 24.39% (95% CI 21.54–27.24%). Mortality rates ranged between 3.26 and 46.71%. An *I*^2^ for heterogeneity of 98.81 indicates high heterogeneity. This analysis covered 170,629 participants from *N* = 72 studies. Mortality rate trends from 2009 to 2018 are reported, and a statistically significant trend in change of mortality rate was found in 2015 (Fig. 5).

Data from RCTs (*N* = 13 studies with 4045 participants) reported an average 30-day sepsis mortality rate of 21.41% (95% CI 16.06–26.77%), compared to prospective cohort studies (*N* = 31 studies with 8827 participants) of 21.70% (95% CI 17.74–25.66%) and retrospective cohort studies (*N* = 16 studies with 27,766 participants) of 23.99% (95% CI 19.67–28.30%) (Fig. 3). The remaining 15 studies were other study types and not reported in this sub analysis.

*N* = 39 studies conducted in Europe with 19,343 participants reported an average 30-day sepsis mortality rate of 23.58% (95% CI 20.61–26.56%), compared to studies conducted in North America (*N* = 16 studies with 19,268 participants) of 19.58% (95% CI 14.03–25.14%) and studies conducted in Australia (*N* = 4 studies with 1661 participants) of 18.72% (95% CI 6.58–30.86%) (Fig. 4).

#### 90-day sepsis mortality

The observed 90-day mortality of sepsis patients was 32.24% (95% CI 27.02–37.46%). Mortality rates ranged between 13.04 and 56.67%. An *I*^2^ for heterogeneity of 98.48 indicates high heterogeneity. This analysis covered 125,717 participants from *N* = 25 studies. From 2010 to 2011, a statistically significant trend towards higher mortality was found, but no change in time since 2012 (Additional file 4: Figure S1).

Data obtained from RCTs (*N* = 9 studies with 3109 participants) reported an average 90-day sepsis mortality of 29.61% (95% CI 22.23–37.00%), compared to prospective cohort studies (*N* = 9 studies with 3408 participants) of 31.66% (95% CI 27.44–35.87%) and retrospective cohort studies (*N* = 3 studies with 110,252 participants) of 29.50% (95% CI 14.10–44.90%) (Fig. 3). The remaining 4 studies were other study types and not reported in this sub analysis.

Due to the low number of studies conducted in North America (*N* = 2) and Australia (*N* = 1), stratification by region was not feasible and therefore not done.

#### Mortality rates for different SOFA scores

**Fig. 6:**
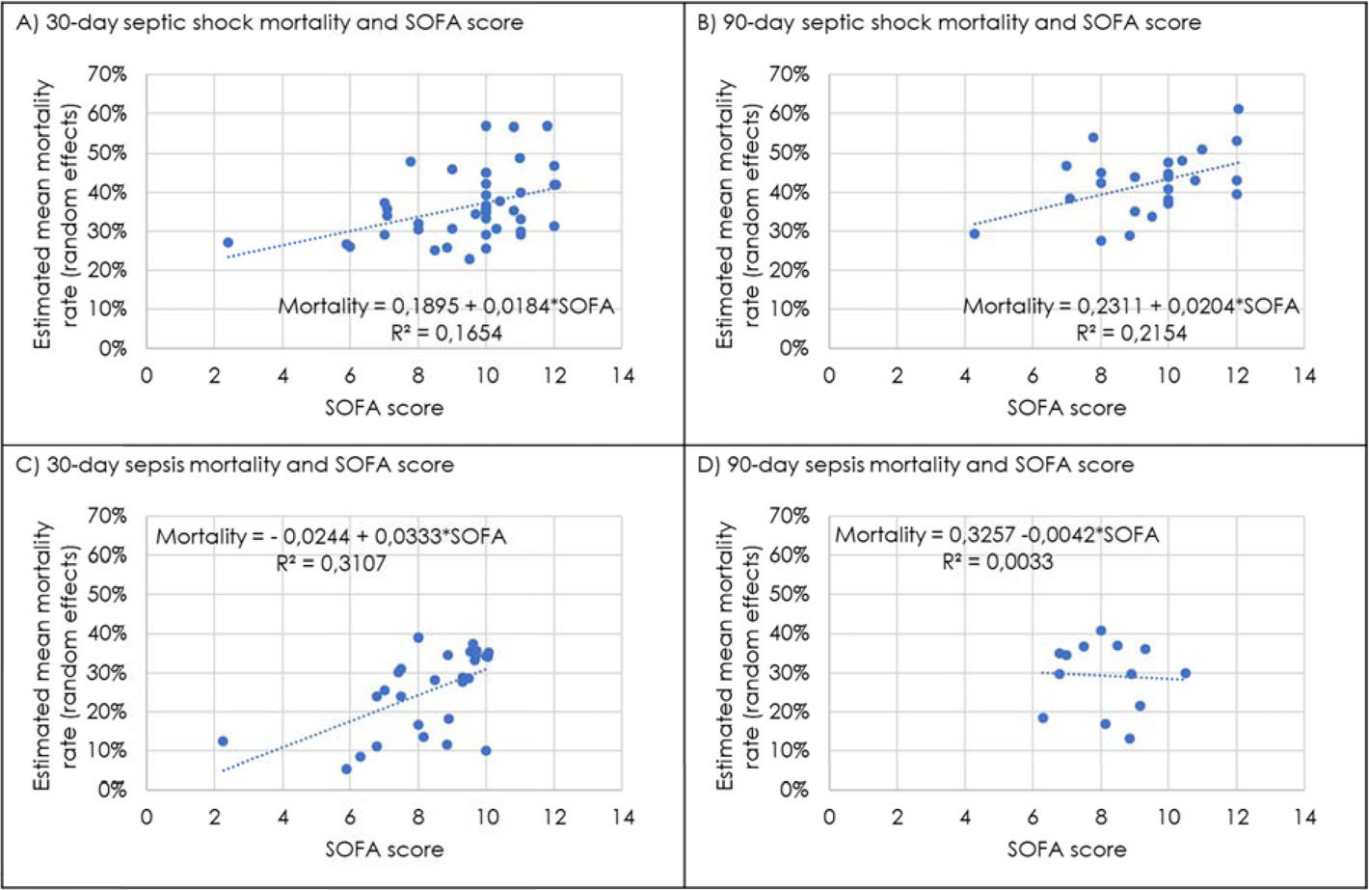
Linear regression of SOFA score and mortality rates. A change in average mortality rates with increasing SOFA scores was found for **a** 30-day septic shock, **b** 90-day septic shock, **c** 30-day sepsis and **d** 90-day sepsis. Graphs show positive correlations of mortality rates and SOFA scores for **a** 30-day septic shock, **b** 90-day septic shock and **c** 30-day sepsis

As expected, a positive correlation between mortality rate and SOFA score was found. A higher average mortality rate with increasing SOFA score was in particular found for mortality 90 days after septic shock, with an increased mortality of 2.4 percentage points per 1-point SOFA score increase (*r*^2^ = 0.2154). Average mortality also increases in 30-day septic shock and 30-day sepsis mortality in study populations with higher SOFA scores (*r*^2^ = 0.1654 and *r*^2^ = 0.3107, respectively). The correlation between 90-day sepsis mortality and SOFA score was slightly negative, but the analysis is based on a low number of studies (Fig. 6). The correlation of participants’ age and mortality rates was positive for septic shock (30-day and 90-day mortality), while correlation of age and sepsis mortality was negative (30-day and 90-day mortality) (Additional file 4: Figure S2).

## Discussion

In this systematic review and meta-analysis, we described estimates of mortality rates for sepsis and septic shock for 30 and 90 days. Our results present a comprehensive view of the mortality in sepsis and septic shock, showing that average 30-day mortality in septic shock was 34.7% (95% CI 32.6–36.9%) and 90-day mortality in septic shock was 38.5% (95% CI 35.4–41.5%).

Well-recognized, previous meta-analyses from Stevenson et al. [8] and Shankar-Hari et al. [21] focused on reporting annual changes in 28-day severe sepsis mortality and estimated crude mortality for septic shock. Stevenson and colleagues included RCTs only and split results for patients inside and outside of the US, without any differences in trends. Furthermore, Stevenson et al. compared results from the meta-analysis with results from a nationwide inpatient sample, showing that mortality rates from clinical studies and administrative sources were similar for the US in the given time period from 1991 to 2009 [8]. Shankar-Hari et al. estimated crude septic shock mortality. In contrast to Stevenson and colleagues, the authors included observational studies only [21] while the specific time periods (dating back into the 1980s), in which mortality was monitored, were not taken into consideration as a confounder. Recently, two meta-analyses were published by Luhr et al. in 2019 [9], which included RCTs up to 2016, and by Vincent et al. in 2019 [22], which included observational studies up to 2018. Luhr et al. reported annual 28-day sepsis mortality over time. Additionally, annual mortality rates were adjusted for the Acute Physiologic Assessment and Chronic Health Evaluation (APACHE) II, simplified acute physiology SAPS II and sequential organ failure assessment (SOFA) scores to estimate the change in mortality rate as a function of time. Vincent et al. reported septic shock mortality rates for several short-term time points (intensive care unit (ICU), hospital and 28/30 days) and compared rates in Europe and North America.

Our numbers are in line with existing literature, e.g. obtained in a large cohort study including 153,257 patients with septic shock reporting a hospital mortality of 35.1% [6] or data obtained by other meta-analysis reporting 33.2% 28-day (severe) sepsis mortality and 28-/30-day septic shock mortality of 36.7%, respectively [9].

Substantial differences appear, however, when comparing our mortality rates for septic shock to the crude mortality rate reported by Shankar-Hari with 46.5%. This probably reflects inclusion of long-term outcome data in this meta-analysis. In addition, Shankar-Hari only included observational studies [21].

Mortality trends over time indicate decreasing rates for patients with sepsis. In contrast, (30-day) mortality rates for septic shock patients dropped from 2009 to 2011 as well but have not continued to do so since 2012. Thus, in contrast to previous publications, we did not find a continuous decline in mortality rates over the last 5–7 years. Luhr et al. also reported an annual decline in mortality rates, but when adjusting mortality rates for APACHE II, SAPS II and SOFA scores, this difference disappeared, indicative of a trend towards higher disease severity over time [9]. This confirms our findings, as there was no continuous decline in mortality rates visible for septic shock patients either.

Our analyses indicated heterogeneity in mortality rates per region. However, no trend showing that one region had consistently higher mortality rates than others across all analyses (30-/90-day sepsis/septic shock mortality) was apparent. One possible reason therefore could be anchored in the partly small number of studies per region. The analysis per region with the highest number of studies was the 30-day septic shock mortality analysis, with *n* = 46 studies from Europe, *n* = 12 studies from North America and *n* = 2 studies from Australia, resulting in mortality rates for studies conducted in North America of 33.69%, in Europe of 32.49% and in Australia of 26.38%. These results are in line with the meta-analysis of Vincent et al., who also reported no statistically significant differences in mortality rates between Europe and North America (28-/30-day mortality in Europe, 38.5%; in North America, 33.2%). Vincent et al. included observational studies only [22].

This meta-analysis has several limitations. First, search was carried out in only PubMed and Cochrane Library for systematic reviews and studies in languages other than English were excluded. Second, inclusion criteria in included studies were heterogenous, which led to a wide range of reported mortality rates. This limitation of heterogeneity also applied to and was reported in Luhr’s meta-analysis, which used narrower inclusion criteria that included RCTs only. Third, in 2016, a new sepsis definition was published [23]. Our meta-analysis comprised of both studies that applied the sepsis-1/2 [14] definition and studies that used the sepsis-3 definition [15]. Since the new definition was published in 2016, our meta-analysis included 6 [24–29] studies (less than 5% of all included studies) that applied the updated definition. We classified risk of bias due to multiple sepsis definitions as low, because mortality rates of half of the corresponding studies lied in confidence intervals for sepsis/septic shock 30-/90-day mortality estimated in our analysis. Fourth, the high amount of multi-country studies that were conducted in more than one geographic region could not be assigned to one region and therefore were not considered for stratification by geographic region. Last, studies from countries outside of Europe, North America and Australia (incl. New Zealand) were not included in the current analysis.

This meta-analysis has several strengths. The broad inclusion criteria regarding the inclusion of interventional and observational studies set this meta-analysis apart from previous publications, which included either RCTs [8, 9] or observational studies [21, 22] only. As patients in RCTs were often recruited by narrow criteria, studies based on prospective or retrospective cohorts, represent a more realistic range of patients with sepsis and septic shock. Our results indicate that mortality rates for 30 days, as well as 90 days for septic shock, derived from RCTs range slightly below the rates of other study types, underlying the importance of the inclusion of several study types to obtain comprehensive estimates of sepsis mortality. By also including a broad range of studies, the total number of included patients (*n* = 371,937) is roughly 25 times higher than that of previous meta-analyses [8, 9].

## Conclusion

Despite some evidence of a trend towards lower mortality rates in sepsis patients over the past decade, a continuous reduction in mortality was not observed among sepsis or septic shock patients. This trend, and the regional disparities, may indicate an ongoing unmet need for improving sepsis management. The analysis of pure mortality rates should be accompanied by consideration of the burden of disease over time. As expected, the average mortality rates are higher in study populations with higher baseline SOFA scores, and an increasing burden of disease could potentially moderate mortality rate trends. Therefore, further investigation is needed to address this correlation over time.

## Supplementary information

**Additional file 1: ** Systematic Review – Search Term. **Table S1.** Search Term/Filter. **Table S2.** Search Term/Filter (PubMed hits for each part of the Search Term). Presentation of Search Term and hits on PubMed for each part of the Search term.

**Additional file 2.** Reference list. List of references included in the meta-analysis.

**Additional file 3.** Table of studies per endpoint and Risk of bias assessment. Table of studies shows which study was used for which endpoint in the meta-analysis.

**Additional file 4: **Further results. **Figure S1.** Joinpoint estimation of 90-day sepsis mortality. Results from the estimation of pooled 90-day sepsis mortality rates per year. The Joinpoint analysis shows the timepoint when trends in mortality rates change. A trend change of 90-day sepsis mortality was found in 2011. **Figure S2.** Linear regression of age and mortality rates. Correlation of mortality rates and age were estimated for A) 30-day septic shock B) 90-day septic shock C) 30-day sepsis and D) 90-day sepsis.

**Additional file 5.** List of excluded studies after full-text screening. Table includes all studies, that were excluded after full-text review with reason for exclusion.

## Data Availability

The datasets generated and/or analysed during the current study are not publicly available but are available from the corresponding author on reasonable request.
