# Supplementary material for: Mortality in sepsis and septic shock in Europe, North America and Australia between 2009 and 2019— results from a systematic review and meta-analysis
Source: Crit Care. 2020 May 19;24:239. doi: 10.1186/s13054-020-02950-2 (PMC7236499; doi:10.1186/s13054-020-02950-2)
Supplement: Supplementary file 1 — Additional file 1: Systematic Review – Search Term. Table S1. Search Term/Filter. Table S2. Search Term/Filter (PubMed hits for each part of the Search Term). Presentation of Search Term and hits on PubMed for each part of the Search term. [file 13054_2020_2950_MOESM1_ESM.docx]

# Additional file 1: Systematic Review – Search Term

Table S1: Search Term/Filter (PubMed hits for each part of the Search Term)

| Criteria | Attributes to be included | Search term |
| --- | --- | --- |
| Patient | Adult patients with sepsis or septic shock | “Sepsis” / “septic shock” |
| Intervention | Standard of Care | -- |
| Comparison | -- | -- |
| Outcomes I | Length of:   - State: septic shock - State: hemodynamic stable sepsis - State: multi-organ failure | “length of stay”, “hospital stay” “hospital stays” “ICU stay”, “ICU stays”, “second hit” |
| Outcomes II | Probability of death:   - State: septic shock - State: hemodynamic stable sepsis - State: multi-organ failure - State: Post multi organ failure - State: Post sepsis | “mortality”, “survival”, “death”, “fatal”, “fatality” |
| Outcomes III | Probability of multi-organ failure:   - State: septic shock - State: hemodynamic stable sepsis   Probability for death for state:   - State: multi-organ failure - Post-multi organ failure   Length of:   - Multi organ failure | “multi-organ failure”, “organ failure”, “organ dysfunction”, “organ insufficiency”  **Kidney**  “kidney failure”, “kidney dysfunction”, “kidney insufficiency”, “acute kidney injury”, “renal failure”, “renal dysfunction”, “renal insufficiency”, “acute renal injury”  **Liver**  “liver failure”, “liver dysfunction”, “liver insufficiency”, “acute liver injury”  **Lung**  “lung failure”, “lung dysfunction”, “acute liver injury”, “lung insufficiency” “pulmonary failure”, “pulmonary dysfunction”, “acute pulmonary injury”, “pulmonary insufficiency”, “respiratory failure”, “respiratory dysfunction”, “respiratory insufficiency” |
| Design | All Designs |  |
| Limits | Humans  English language  Year 2008 onwards |  |

**Search Term (overall)**

(Sepsis[MeSH] OR Sepsis[Title/Abstract] OR “septic shock”[Title/Abstract])

**AND**

((“length of stay”[MeSH] OR “length of stay”[Title/Abstract] OR “hospital stay”[Title/Abstract] OR “hospital stays”[Title/Abstract] OR “ICU stay”[Title/Abstract] OR “intensive care unit stay”[Title/Abstract] OR “ICU stays”[Title/Abstract] OR “intensive care unit stays”[Title/Abstract] OR “second hit”[Title/Abstract] )

**OR**

(“mortality”[MeSH] OR “mortality”[Title/Abstract] OR “survival”[Title/Abstract] OR “survival”[MeSH] OR “fatal”[Title/Abstract] OR “fatality”[Title/Abstract])

**OR**

(“Multiple organ failure”[MeSH] OR “multiple organ dysfunction”[Title/Abstract] OR “MODS”[Title/Abstract] OR “organ failure”[Title/Abstract] OR “organ dysfunction”[Title/Abstract] OR “organ insufficiency”[Title/Abstract])

**OR**

(“acute kidney injury”[MeSH] OR “kidney dysfunction”[Title/Abstract] OR “kidney insufficiency”[Title/Abstract] OR “acute kidney injury”[Title/Abstract] OR “renal failure”[Title/Abstract] OR “renal dysfunction”[Title/Abstract] OR “renal insufficiency”[Title/Abstract] OR “acute renal injury”[Title/Abstract])

**OR**

("liver failure, acute”[MeSH] OR “acute liver failure”[Title/Abstract] OR “liver dysfunction”[Title/Abstract] OR “liver insufficiency”[Title/Abstract] OR “acute liver injury”[Title/Abstract] OR “hepatic failure”[Title/Abstract])

**OR**

(“acute lung injury”[MeSH] OR “lung failure”[Title/Abstract] OR “lung dysfunction”[Title/Abstract] OR “acute liver injury”[Title/Abstract] OR “lung insufficiency”[Title/Abstract] OR “pulmonary failure”[Title/Abstract] OR “pulmonary dysfunction”[Title/Abstract] OR “acute pulmonary injury”[Title/Abstract] OR “pulmonary insufficiency”[Title/Abstract] OR “respiratory failure”[Title/Abstract] OR “respiratory dysfunction”[Title/Abstract] OR “respiratory insufficiency”[Title/Abstract]))

**Search Term (simplified)**

#43 AND (#44 OR #46 OR #48 OR #76 OR #82)) AND ((Clinical Study[ptyp] OR Clinical Trial[ptyp] OR Clinical Trial, Phase I[ptyp] OR Clinical Trial, Phase III[ptyp] OR Clinical Trial, Phase II[ptyp] OR Clinical Trial, Phase IV[ptyp] OR Comparative Study[ptyp] OR Controlled Clinical Trial[ptyp] OR Meta-Analysis[ptyp] OR Observational Study[ptyp] OR Pragmatic Clinical Trial[ptyp] OR Randomized Controlled Trial[ptyp] OR systematic[sb]) AND hasabstract[text] AND "2009/01/01"[PDat] : "2019/03/27"[PDat] AND English[lang]) Sort by: PublicationDate

Table S2: Search Term/Filter (PubMed hits for each part of the Search Term)

| #1 | Sepsis[MeSH] OR Sepsis[Title/Abstract] OR “septic shock”[Title/Abstract] | 174,072 |
| --- | --- | --- |
| #2 | “length of stay”[MeSH] OR “length of stay”[Title/Abstract] OR “hospital stay”[Title/Abstract] OR “hospital stays”[Title/Abstract] OR “ICU stay”[Title/Abstract] OR “intensive care unit stay”[Title/Abstract] OR “ICU stays”[Title/Abstract] OR “intensive care unit stays”[Title/Abstract] OR “second hit”[Title/Abstract] | 159,860 |
| #3 | “mortality”[MeSH] OR “mortality”[Title/Abstract] OR “survival”[Title/Abstract] OR “survival”[MeSH] OR “fatal”[Title/Abstract] OR “fatality”[Title/Abstract] | 1,659,653 |
| #4 | “Multiple organ failure”[MeSH] OR “multiple organ dysfunction”[Title/Abstract] OR “MODS”[Title/Abstract] OR “organ failure”[Title/Abstract] OR “organ dysfunction”[Title/Abstract] OR “organ insufficiency”[Title/Abstract] | 31,684 |
| #5 | “acute kidney injury”[MeSH] OR “kidney dysfunction”[Title/Abstract] OR “kidney insufficiency”[Title/Abstract] OR “acute kidney injury”[Title/Abstract] OR “renal failure”[Title/Abstract] OR “renal dysfunction”[Title/Abstract] OR “renal insufficiency”[Title/Abstract] OR “acute renal injury”[Title/Abstract] | 149,164 |
| #6 | "liver failure, acute”[MeSH] OR “acute liver failure”[Title/Abstract] OR “liver dysfunction”[Title/Abstract] OR “liver insufficiency”[Title/Abstract] OR “acute liver injury”[Title/Abstract] OR “hepatic failure”[Title/Abstract] | 25,406 |
| #7 | “acute lung injury”[MeSH] OR “lung failure”[Title/Abstract] OR “lung dysfunction”[Title/Abstract] OR “acute liver injury”[Title/Abstract] OR “lung insufficiency”[Title/Abstract] OR “pulmonary failure”[Title/Abstract] OR “pulmonary dysfunction”[Title/Abstract] OR “acute pulmonary injury”[Title/Abstract] OR “pulmonary insufficiency”[Title/Abstract] OR “respiratory failure”[Title/Abstract] OR “respiratory dysfunction”[Title/Abstract] OR “respiratory insufficiency”[Title/Abstract] | 49,637 |
| #8 | (#4 OR #5 OR #6 OR #7) | 244,078 |
| #9 | #2 OR #3 OR #8 | 1,938,787 |
| #10 | #1 AND #9 | 63,689 |
| #11 | +Filter: Study type: Clinical Study, Clinical Trial, Clinical Trial, Phase I, Clinical Trial, Phase II, Clinical Trial, Phase III, Clinical Trial, Phase IV, Comparative Study, Controlled Clinical Trial, Meta-Analysis, Observational Study, Pragmatic Clinical Trial, Randomized Controlled Trial, Systematic Reviews. | 9,771 |
| #12 | +Filter: 01.01.2009 – 27.03.2019 | 4,938 |
| #13 | +Filter: Text availability: Abstract | 4,918 |
| #14 | +Filter: Species Humans | 4,596 |
| #15 | +Filter: English language | 4,494 |
