# Supplementary material for: Mortality in sepsis and septic shock in Europe, North America and Australia between 2009 and 2019— results from a systematic review and meta-analysis
Source: Crit Care. 2020 May 19;24:239. doi: 10.1186/s13054-020-02950-2 (PMC7236499; doi:10.1186/s13054-020-02950-2)
Supplement: Supplementary file 2 — Additional file 2. Reference list. List of references included in the meta-analysis. [file 13054_2020_2950_MOESM2_ESM.docx]

# Additional file 2: Reference list

Alvarez J, Mar J, Varela-Ledo E, Garea M, Matinez-Lamas L, Rodriguez J, Regueiro B. Anaesth Intensive Care. 2012;40(6):958-63.

Alhazzani W, Jacobi J, Sindi A, Hartog C, Reinhart K, Kokkoris S, et al. The effect of selenium therapy on mortality in patients with sepsis syndrome: a systematic review and meta-analysis of randomized controlled trials. Crit Care Med. 2013;41(6):1555-64.

Angus DC, Barnato AE, Bell D, Bellomo R, Chong CR, Coats TJ, et al. A systematic review and meta-analysis of early goal-directed therapy for septic shock: the ARISE, ProCESS and ProMISe Investigators. Intensive Care Med. 2015;41(9):1549-60.

Annane D, Bellissant E, Bollaert PE, Briegel J, Keh D, Kupfer Y. Corticosteroids for treating sepsis. Cochrane Database Syst Rev. 2015;(12):CD002243.

Annane D, Bellissant E, Bollaert PE, Briegel J, Confalonieri M, De Gaudio R, et al. Corticosteroids in the treatment of severe sepsis and septic shock in adults: a systematic review. JAMA. 2009;301(22):2362-75.

COIITSS Study Investigators, Annane D, Cariou A, Maxime V, Azoulay E, D'honneur G, et al. Corticosteroid treatment and intensive insulin therapy for septic shock in adults: a randomized controlled trial. JAMA. 2010;303(4):341-8.

Annane D, Renault A, Brun-Buisson C, Megarbane B, Quenot JP, Siami S, et al. Hydrocortisone plus Fludrocortisone for Adults with Septic Shock. N Engl J Med. 2018;378(9):809-818.

Annane D, Timsit JF, Megarbane B, Martin C, Misset B, Mourvillier B, et al. Recombinant human activated protein C for adults with septic shock: a randomized controlled trial. Am J Respir Crit Care Med. 2013;187(10):1091-7.

Asfar P, Meziani F, Hamel JF, Grelon F, Megarbane B, Anguel N, et al. High versus low blood-pressure target in patients with septic shock. N Engl J Med. 2014;370(17):1583-93.

Asfar P, Schortgen F, Boisramé-Helms J, Charpentier J, Guérot E, Megarbane B, et al. Hyperoxia and hypertonic saline in patients with septic shock (HYPERS2S): a two-by-two factorial, multicentre, randomised, clinical trial. Lancet Respir Med. 2017;5(3):180-190.

Askim Å, Moser F, Gustad LT, Stene H, Gundersen M, Åsvold BO, et al. Poor performance of quick-SOFA (qSOFA) score in predicting severe sepsis and mortality - a prospective study of patients admitted with infection to the emergency department. Scand J Trauma Resusc Emerg Med. 2017;25(1):56.

Avni T, Lador A, Lev S, Leibovici L, Paul M, Grossman A. Vasopressors for the Treatment of Septic Shock: Systematic Review and Meta-Analysis. PLoS One. 2015;10(8):e0129305.

Balik M, Kolnikova I, Maly M, Waldauf P, Tavazzi G, Kristof J. Propafenone for supraventricular arrhythmias in septic shock-Comparison to amiodarone and metoprolol. J Crit Care. 2017;41:16-23.

Behnes M, Bertsch T, Lepiorz D, Lang S, Trinkmann F, Brueckmann M, et al. Diagnostic and prognostic utility of soluble CD 14 subtype (presepsin) for severe sepsis and septic shock during the first week of intensive care treatment. Crit Care. 2014;18(5):507.

Bernard GR, Francois B, Mira JP, Vincent JL, Dellinger RP, Russell JA, et al. Evaluating the efficacy and safety of two doses of the polyclonal anti-tumor necrosis factor-α fragment antibody AZD9773 in adult patients with severe sepsis and/or septic shock: randomized, double-blind, placebo-controlled phase IIb study*. Crit Care Med. 2014;42(3):504-11.

Biyikli E, Kayipmaz AE, Kavalci C. Effect of platelet-lymphocyte ratio and lactate levels obtained on mortality with sepsis and septic shock. Am J Emerg Med. 2018;36(4):647-650.

Bloos F, Rüddel H, Thomas-Rüddel D, Schwarzkopf D, Pausch C, Harbarth S, et al. Effect of a multifaceted educational intervention for anti-infectious measures on sepsis mortality: a cluster randomized trial. Intensive Care Med. 2017;43(11):1602-1612.

Bloos F, Thomas-Rüddel D, Rüddel H, Engel C, Schwarzkopf D, Marshall JC, et al. Impact of compliance with infection management guidelines on outcome in patients with severe sepsis: a prospective observational multi-center study. Crit Care. 2014;18(2):R42

Bloos F, Trips E, Nierhaus A, Briegel J, Heyland DK, Jaschinski U, et al. Effect of Sodium Selenite Administration and Procalcitonin-Guided Therapy on Mortality in Patients With Severe Sepsis or Septic Shock: A Randomized Clinical Trial. JAMA Intern Med. 2016;176(9):1266-76.

Borthwick EM, Hill CJ, Rabindranath KS, Maxwell AP, McAuley DF, Blackwood B. High-volume haemofiltration for sepsis in adults. Cochrane Database Syst Rev. 2017;1:CD008075.

Boulain T, Garot D, Vignon P, Lascarrou JB, Desachy A, Botoc V, et al. Prevalence of low central venous oxygen saturation in the first hours of intensive care unit admission and associated mortality in septic shock patients: a prospective multicentre study. Crit Care. 2014;18(6):609.

Boulain T, Runge I, Bercault N, Benzekri-Lefevre D, Wolf M, Fleury C. Dopamine therapy in septic shock: detrimental effect on survival? J Crit Care. 2009;24(4):575-82.

Boyd JH, Fjell CD, Russell JA, Sirounis D, Cirstea MS, Walley KR. Increased Plasma PCSK9 Levels Are Associated with Reduced Endotoxin Clearance and the Development of Acute Organ Failures during Sepsis. J Innate Immun. 2016;8(2):211-20.

Brown SM, Tate MQ, Jones JP, Kuttler KG, Lanspa MJ, Rondina MT, et al. Coefficient of Variation of Coarsely Sampled Heart Rate is Associated With Early Vasopressor Independence in Severe Sepsis and Septic Shock. J Intensive Care Med. 2015;30(7):420-5.

Brunkhorst FM, Oppert M, Marx G, Bloos F, Ludewig K, Putensen C, et al. Effect of empirical treatment with moxifloxacin and meropenem vs meropenem on sepsis-related organ dysfunction in patients with severe sepsis: a randomized trial. JAMA. 2012;307(22):2390-9.

Caironi P, Tognoni G, Masson S, Fumagalli R, Pesenti A, Romero M, et al. Albumin replacement in patients with severe sepsis or septic shock. N Engl J Med. 2014;370(15):1412-21.

Castegren M, Jonasson M, Castegren S, Lipcsey M, Sjölin J. Initial levels of organ failure, microbial findings and mortality in intensive care-treated primary, secondary and tertiary sepsis. Crit Care Resusc. 2015;17(3):174-81.

Chan CM, Mitchell AL, Shorr AF. Etomidate is associated with mortality and adrenal insufficiency in sepsis: a meta-analysis*. Crit Care Med. 2012;40(11):2945-53.

Clark E, Molnar AO, Joannes-Boyau O, Honoré PM, Sikora L, Bagshaw SM. High-volume hemofiltration for septic acute kidney injury: a systematic review and meta-analysis. Crit Care. 2014;18(1):R7.

Coccolini F, Sartelli M, Catena F, Ceresoli M, Montori G, Ansaloni L. Early goal-directed treatment versus standard care in management of early septic shock: Meta-analysis of randomized trials. J Trauma Acute Care Surg. 2016;81(5):971-978.

Coen D, Cortellaro F, Pasini S, Tombini V, Vaccaro A, Montalbetti L, et al. Towards a less invasive approach to the early goal-directed treatment of septic shock in the ED. Am J Emerg Med. 2014;32(6):563-8.

Contenti J, Corraze H, Lemoël F, Levraut J. Effectiveness of arterial, venous, and capillary blood lactate as a sepsis triage tool in ED patients. Am J Emerg Med. 2015;33(2):167-72.

Contou D, Roux D, Jochmans S, Coudroy R, Guérot E, Grimaldi D, et al. Septic shock with no diagnosis at 24 hours: a pragmatic multicenter prospective cohort study. Crit Care. 2016;20(1):360.

Cruz DN, Antonelli M, Fumagalli R, Foltran F, Brienza N, Donati A, et al. Early use of polymyxin B hemoperfusion in abdominal septic shock: the EUPHAS randomized controlled trial. JAMA. 2009;301(23):2445-52.

Dahl RM, Grønlykke L, Haase N, Holst LB, Perner A, Wetterslev J, et al. Variability in targeted arterial oxygenation levels in patients with severe sepsis or septic shock. Acta Anaesthesiol Scand. 2015;59(7):859-69.

Davies GR, Lawrence M, Pillai S, Mills GM, Aubrey R, Thomas D, et al. The effect of sepsis and septic shock on the viscoelastic properties of clot quality and mass using rotational thromboelastometry: A prospective observational study. J Crit Care. 2018;44:7-11.

DE LA Torre-Prados MV, Garcia-DE LA Torre A, Enguix A, Mayor-Reyes M, Nieto-González M, Garcia-Alcantara A. Mid-regional pro-adrenomedullin as prognostic biomarker in septic shock. Minerva Anestesiol. 2016;82(7):760-6.

de Pablo R, Monserrat J, Reyes E, Díaz D, Rodríguez-Zapata M, de la Hera A, et al. Circulating sICAM-1 and sE-Selectin as biomarker of infection and prognosis in patients with systemic inflammatory response syndrome. Eur J Intern Med. 2013;24(2):132-8.

Dellinger RP, Bagshaw SM, Antonelli M, Foster DM, Klein DJ, Marshall JC, et al. Effect of Targeted Polymyxin B Hemoperfusion on 28-Day Mortality in Patients With Septic Shock and Elevated Endotoxin Level: The EUPHRATES Randomized Clinical Trial. JAMA. 2018;320(14):1455-1463.

Dettmer M, Holthaus CV, Fuller BM. The impact of serial lactate monitoring on emergency department resuscitation interventions and clinical outcomes in severe sepsis and septic shock: an observational cohort study. Shock. 2015;43(1):55-61.

Drewry AM, Ablordeppey EA, Murray ET, Beiter ER, Walton AH, Hall MW, et al. Comparison of monocyte human leukocyte antigen-DR expression and stimulated tumor necrosis factor alpha production as outcome predictors in severe sepsis: a prospective observational study. Crit Care. 2016;20(1):334.

Dulhunty JM, Roberts JA, Davis JS, Webb SA, Bellomo R, Gomersall C, et al. A Multicenter Randomized Trial of Continuous versus Intermittent β-Lactam Infusion in Severe Sepsis. Am J Respir Crit Care Med. 2015;192(11):1298-305.

Dupuis C, Garrouste-Orgeas M, Bailly S, Adrie C, Goldgran-Toledano D, Azoulay E, et al. Effect of Transfusion on Mortality and Other Adverse Events Among Critically Ill Septic Patients: An Observational Study Using a Marginal Structural Cox Model. Crit Care Med. 2017;45(12):1972-1980.

Dwivedi DJ, Toltl LJ, Swystun LL, Pogue J, Liaw KL, Weitz JI, et al. Prognostic utility and characterization of cell-free DNA in patients with severe sepsis. Crit Care. 2012;16(4):R151.

Elke G, Kuhnt E, Ragaller M, Schädler D, Frerichs I, Brunkhorst FM, et al. Enteral nutrition is associated with improved outcome in patients with severe sepsis. A secondary analysis of the VISEP trial. Med Klin Intensivmed Notfmed. 2013;108(3):223-33.

Ferraris A, Bouisse C, Mottard N, Thiollière F, Anselin S, Piriou V, et al. Mottling score and skin temperature in septic shock: Relation and impact on prognosis in ICU. PLoS One. 2018;13(8):e0202329.

SAFE Study Investigators, Finfer S, McEvoy S, Bellomo R, McArthur C, Myburgh J, et al. Impact of albumin compared to saline on organ function and mortality of patients with severe sepsis. Intensive Care Med. 2011;37(1):86-96.

Fisher J, Douglas JJ, Linder A, Boyd JH, Walley KR, Russell JA. Elevated Plasma Angiopoietin-2 Levels Are Associated With Fluid Overload, Organ Dysfunction, and Mortality in Human Septic Shock. Crit Care Med. 2016;44(11):2018-2027.

Fjell CD, Thair S, Hsu JL, Walley KR, Russell JA, Boyd J. Cytokines and signaling molecules predict clinical outcomes in sepsis. PLoS One. 2013;8(11):e79207.

García-López L, Grau-Cerrato S, de Frutos-Soto A, Bobillo-De Lamo F, Cítores-Gónzalez R, Diez-Gutierrez F, et al. Impact of the implementation of a Sepsis Code hospital protocol in antibiotic prescription and clinical outcomes in an intensive care unit. Med Intensiva. 2017;41(1):12-20.

Gaulton TG, Weiner MG, Morales KH, Gaieski DF, Mehta J, Lautenbach E. The effect of obesity on clinical outcomes in presumed sepsis: a retrospective cohort study. Intern Emerg Med. 2014;9(2):213-21.

Giamarellos-Bourboulis EJ, Mylona V, Antonopoulou A, Tsangaris I, Koutelidakis I, Marioli A, et al. Effect of clarithromycin in patients with suspected Gram-negative sepsis: results of a randomized controlled trial. J Antimicrob Chemother. 2014;69(4):1111-8.

Gonzalez-Padilla M, Torre-Cisneros J, Rivera-Espinar F, Pontes-Moreno A, López-Cerero L, Pascual A, et al. Gentamicin therapy for sepsis due to carbapenem-resistant and colistin-resistant Klebsiella pneumoniae. J Antimicrob Chemother. 2015;70(3):905-13.

Gordon AC, Mason AJ, Thirunavukkarasu N, Perkins GD, Cecconi M, Cepkova M, et al. Effect of Early Vasopressin vs Norepinephrine on Kidney Failure in Patients With Septic Shock: The VANISH Randomized Clinical Trial. JAMA. 2016;316(5):509-18.

Gordon AC, Russell JA, Walley KR, Singer J, Ayers D, Storms MM, et al. The effects of vasopressin on acute kidney injury in septic shock. Intensive Care Med. 2010;36(1):83-91.

Guirgis FW, Dodani S, Leeuwenburgh C, Moldawer L, Bowman J, Kalynych C, et al. HDL inflammatory index correlates with and predicts severity of organ failure in patients with sepsis and septic shock. PLoS One. 2018;13(9):e0203813.

Hammond DA, Ficek OA, Painter JT, McCain K, Cullen J, Brotherton AL, et al. Prospective Open-label Trial of Early Concomitant Vasopressin and Norepinephrine Therapy versus Initial Norepinephrine Monotherapy in Septic Shock. Pharmacotherapy. 2018;38(5):531-538.

Hanzelka KM, Yeung SC, Chisholm G, Merriman KW, Gaeta S, Malik I, et al. Implementation of modified early-goal directed therapy for sepsis in the emergency center of a comprehensive cancer center. Support Care Cancer. 2013;21(3):727-34.

Hazzard I, Jones S, Quinn T. Coupled plasma haemofiltration filtration in severe sepsis: systematic review and meta-analysis. J R Army Med Corps. 2015;161 Suppl 1:i17-i22.

Heemskerk S, Masereeuw R, Moesker O, Bouw MP, van der Hoeven JG, Peters WH, et al. Alkaline phosphatase treatment improves renal function in severe sepsis or septic shock patients. Crit Care Med. 2009;37(2):417-23, e1.

Herrán-Monge R, Muriel-Bombín A, García-García MM, Merino-García PA, Cítores-González R, Fernández-Ratero JA, et al. Mortality Reduction and Long-Term Compliance with Surviving Sepsis Campaign: A Nationwide Multicenter Study. Shock. 2016;45(6):598-606.

Hjortrup PB, Haase N, Bundgaard H, Thomsen SL, Winding R, Pettilä V, et al. Restricting volumes of resuscitation fluid in adults with septic shock after initial management: the CLASSIC randomised, parallel-group, multicentre feasibility trial. Intensive Care Med. 2016;42(11):1695-1705.

Holst LB, Haase N, Wetterslev J, Wernerman J, Guttormsen AB, Karlsson S, et al. Lower versus higher hemoglobin threshold for transfusion in septic shock. N Engl J Med. 2014;371(15):1381-91.

Igonin AA, Protsenko DN, Galstyan GM, Vlasenko AV, Khachatryan NN, Nekhaev IV, et al. C1-esterase inhibitor infusion increases survival rates for patients with sepsis*. Crit Care Med. 2012;40(3):770-7.

Jerwood S, Hankins M, Cohen J. A pilot clinical trial to evaluate a novel time-to-positivity assay to measure the effectiveness of antibiotic therapy for septic patients in intensive care. J Crit Care. 2012;27(3):320.e1-5.

Joannes-Boyau O, Honoré PM, Perez P, Bagshaw SM, Grand H, Canivet JL, et al. High-volume versus standard-volume haemofiltration for septic shock patients with acute kidney injury (IVOIRE study): a multicentre randomized controlled trial. Intensive Care Med. 2013 Sep;39(9):1535-46.

Johansen ME, Johansson PI, Ostrowski SR, Bestle MH, Hein L, Jensen AL, et al. Profound endothelial damage predicts impending organ failure and death in sepsis. Semin Thromb Hemost. 2015;41(1):16-25.

Jouffroy R, Saade A, Carpentier A, Ellouze S, Philippe P, Idialisoa R, et al. Triage of Septic Patients Using qSOFA Criteria at the SAMU Regulation: A Retrospective Analysis. Prehosp Emerg Care. 2018;22(1):84-90.

Jung B, Clavieras N, Nougaret S, Molinari N, Roquilly A, Cisse M, et al. Effects of etomidate on complications related to intubation and on mortality in septic shock patients treated with hydrocortisone: a propensity score analysis. Crit Care. 2012;16(6):R224.

Kaffarnik MF, Lock JF, Vetter H, Ahmadi N, Lojewski C, Malinowski M, et al. Early diagnosis of sepsis-related hepatic dysfunction and its prognostic impact on survival: a prospective study with the LiMAx test. Crit Care. 2013;17(5):R259.

Keep JW, Messmer AS, Sladden R, Burrell N, Pinate R, Tunnicliff M, et al. National early warning score at Emergency Department triage may allow earlier identification of patients with severe sepsis and septic shock: a retrospective observational study. Emerg Med J. 2016;33(1):37-41.

Keh D, Trips E, Marx G, Wirtz SP, Abduljawwad E, Bercker S, et al. Effect of Hydrocortisone on Development of Shock Among Patients With Severe Sepsis: The HYPRESS Randomized Clinical Trial. JAMA. 2016;316(17):1775-1785.

Khoury J, Arow M, Elias A, Makhoul BF, Berger G, Kaplan M, et al. The prognostic value of brain natriuretic peptide (BNP) in non-cardiac patients with sepsis, ultra-long follow-up. J Crit Care. 2017;42:117-122.

Koch A, Sanson E, Helm A, Voigt S, Trautwein C, Tacke F. Regulation and prognostic relevance of serum ghrelin concentrations in critical illness and sepsis. Crit Care. 2010;14(3):R94.

Kristof K, Büttner B, Grimm A, Mewes C, Schmack B, Popov AF, et al. Anaemia requiring red blood cell transfusion is associated with unfavourable 90-day survival in surgical patients with sepsis. BMC Res Notes. 2018;11(1):879.

Kruger P, Bailey M, Bellomo R, Cooper DJ, Harward M, Higgins A, et al. A multicenter randomized trial of atorvastatin therapy in intensive care patients with severe sepsis. Am J Respir Crit Care Med. 2013;187(7):743-50.

Kuipers S, Klein Klouwenberg PM, Cremer OL. Incidence, risk factors and outcomes of new-onset atrial fibrillation in patients with sepsis: a systematic review. Crit Care. 2014;18(6):688.

Kumar A, Zarychanski R, Light B, Parrillo J, Maki D, Simon D, et al. Early combination antibiotic therapy yields improved survival compared with monotherapy in septic shock: a propensity-matched analysis. Crit Care Med. 2010;38(9):1773-85.

Lai PS, Matteau A, Iddriss A, Hawes JC, Ranieri V, Thompson BT. An updated meta-analysis to understand the variable efficacy of drotrecogin alfa (activated) in severe sepsis and septic shock. Minerva Anestesiol. 2013;79(1):33-43.

Laribi S, Lienart D, Castanares-Zapatero D, Collienne C, Wittebole X, Laterre PF. CT-proAVP IS NOT A GOOD PREDICTOR OF VASOPRESSOR NEED IN SEPTIC SHOCK. Shock. 2015;44(4):330-5.

Leaf DE, Raed A, Donnino MW, Ginde AA, Waikar SS. Randomized controlled trial of calcitriol in severe sepsis. Am J Respir Crit Care Med. 2014;190(5):533-41.

Li C, Bo L, Liu Q, Jin F. Thymosin alpha1 based immunomodulatory therapy for sepsis: a systematic review and meta-analysis. Int J Infect Dis. 2015;33:90-6.

López-Mestanza C, Andaluz-Ojeda D, Gómez-López JR, Bermejo-Martín JF. Clinical factors influencing mortality risk in hospital-acquired sepsis. J Hosp Infect. 2018;98(2):194-201.

Lorente L, Martín MM, Abreu-González P, de la Cruz T, Ferreres J, Solé-Violán J, et al. Serum melatonin levels are associated with mortality in severe septic patients. J Crit Care. 2015;30(4):860.e1-6.

Lorente L, Martín MM, Abreu-González P, Domínguez-Rodriguez A, Labarta L, Díaz C, et al. Sustained high serum malondialdehyde levels are associated with severity and mortality in septic patients. Crit Care. 2013;17(6):R290.

Lorente L, Martín MM, Borreguero-León JM, Solé-Violán J, Ferreres J, Labarta L, et al. Sustained high plasma plasminogen activator inhibitor-1 levels are associated with severity and mortality in septic patients. Thromb Res. 2014;134(1):182-6.

Lorente L, Martín MM, González-Rivero AF, Ferreres J, Solé-Violán J, Labarta L, et al. Serum levels of caspase-cleaved cytokeratin-18 and mortality are associated in severe septic patients: pilot study. PLoS One. 2014;9(10):e109618.

Lorente L, Martín MM, López-Gallardo E, Ferreres J, Solé-Violán J, Labarta L, et al. Septic patients with mitochondrial DNA haplogroup JT have higher respiratory complex IV activity and survival rate. J Crit Care. 2016;33:95-9.

Lorente L, Martín MM, López-Gallardo E, Iceta R, Blanquer J, Solé-Violán J, et al. Higher platelet cytochrome oxidase specific activity in surviving than in non-surviving septic patients. Crit Care. 2014;18(3):R136.

Lorente L, Martín MM, Solé-Violán J, Blanquer J, Labarta L, Díaz C, et al. Association of sepsis-related mortality with early increase of TIMP-1/MMP-9 ratio. PLoS One. 2014;9(4):e94318.

Lorente L, Martín MM, Varo N, Borreguero-León JM, Solé-Violán J, Blanquer J, et al. Association between serum soluble CD40 ligand levels and mortality in patients with severe sepsis. Crit Care. 2011;15(2):R97.

Lv S, Han M, Yi R, Kwon S, Dai C, Wang R. Anti-TNF-α therapy for patients with sepsis: a systematic meta-analysis. Int J Clin Pract. 2014;68(4):520-8.

Mansur A, Steinau M, Popov AF, Ghadimi M, Beissbarth T, Bauer M, et al. Impact of statin therapy on mortality in patients with sepsis-associated acute respiratory distress syndrome (ARDS) depends on ARDS severity: a prospective observational cohort study. BMC Med. 2015;13:128.

Mansur A, Klee Y, Popov AF, Erlenwein J, Ghadimi M, Beissbarth T, et al. Primary bacteraemia is associated with a higher mortality risk compared with pulmonary and intra-abdominal infections in patients with sepsis: a prospective observational cohort study. BMJ Open. 2015;5(1):e006616.

Monti G, Terzi V, Calini A, Di Marco F, Cruz D, Pulici M, et al. Rescue therapy with polymyxin B hemoperfusion in high-dose vasopressor therapy refractory septic shock. Minerva Anestesiol. 2015;81(5):516-25.

Mouncey PR, Osborn TM, Power GS, Harrison DA, Sadique MZ, Grieve RD, et al. Protocolised Management In Sepsis (ProMISe): a multicentre randomised controlled trial of the clinical effectiveness and cost-effectiveness of early, goal-directed, protocolised resuscitation for emerging septic shock. Health Technol Assess. 2015;19(97):i-xxv, 1-150.

Nardi O, Zavala E, Martin C, Nanas S, Scheeren T, Polito A, et al. Targeting skeletal muscle tissue oxygenation (StO2) in adults with severe sepsis and septic shock: a randomised controlled trial (OTO-StS Study). BMJ Open. 2018;8(3):e017581.

Nejat M, Pickering JW, Walker RJ, Westhuyzen J, Shaw GM, Frampton CM, et al. Urinary cystatin C is diagnostic of acute kidney injury and sepsis, and predicts mortality in the intensive care unit. Crit Care. 2010;14(3):R85.

Nikitas N, Kopterides P, Ilias I, Theodorakopoulou M, Vassiliadi DA, Armaganidis A, et al. Elevated adipose tissue lactate to pyruvate (L/P) ratio predicts poor outcome in critically ill patients with septic shock: a microdialysis study. Minerva Anestesiol. 2013;79(11):1229-37.

Nowak RM, Nanayakkara P, DiSomma S, Levy P, Schrijver E, Huyghe R, et al. Noninvasive hemodynamic monitoring in emergency patients with suspected heart failure, sepsis and stroke: the PREMIUM registry.West J Emerg Med. 2014;15(7):786-94.

Pandharipande PP, Sanders RD, Girard TD, McGrane S, Thompson JL, Shintani AK, et al. Effect of dexmedetomidine versus lorazepam on outcome in patients with sepsis: an a priori-designed analysis of the MENDS randomized controlled trial. Crit Care. 2010;14(2):R38.

Parsons EC, Hough CL, Seymour CW, Cooke CR, Rubenfeld GD, Watkins TR; et al. Red blood cell transfusion and outcomes in patients with acute lung injury, sepsis and shock. Crit Care. 2011;15(5):R221.

Patel GP, Grahe JS, Sperry M, Singla S, Elpern E, Lateef O, et al. Efficacy and safety of dopamine versus norepinephrine in the management of septic shock. Shock. 2010;33(4):375-80

Payen D, Luengo C, Heyer L, Resche-Rigon M, Kerever S, Damoisel C, et al. Is thenar tissue hemoglobin oxygen saturation in septic shock related to macrohemodynamic variables and outcome? Crit Care. 2009;13 Suppl 5:S6.

Payen D, Mateo J, Cavaillon JM, Fraisse F, Floriot C, Vicaut E; Hemofiltration and Sepsis Group of the Collège National de Réanimation et de Médecine d'Urgence des Hôpitaux extra-Universitaires. Impact of continuous venovenous hemofiltration on organ failure during the early phase of severe sepsis: a randomized controlled trial. Crit Care Med. 2009;37(3):803-10.

Perner A, Haase N, Guttormsen AB, Tenhunen J, Klemenzson G, Åneman A, et al. Hydroxyethyl starch 130/0.42 versus Ringer's acetate in severe sepsis. N Engl J Med. 2012;367(2):124-34.

Poukkanen M, Koskenkari J, Vaara ST, Pettilä V, Karlsson S, Korhonen AM, et al. Variation in the use of renal replacement therapy in patients with septic shock: a substudy of the prospective multicenter observational FINNAKI study. Crit Care. 2014;18(1):R26.

Prescott HC. Variation in Postsepsis Readmission Patterns: A Cohort Study of Veterans Affairs Beneficiaries. Ann Am Thorac Soc. 2017;14(2):230-237.

Prescott HC, Osterholzer JJ, Langa KM, Angus DC, Iwashyna TJ. Late mortality after sepsis: propensity matched cohort study. BMJ. 2016;353:i2375.

Puskarich MA, Kline JA, Krabill V, Claremont H, Jones AE. Preliminary safety and efficacy of L-carnitine infusion for the treatment of vasopressor-dependent septic shock: a randomized control trial. JPEN J Parenter Enteral Nutr. 2014;38(6):736-43.

Puskarich MA, Kline JA, Watts JA, Shirey K, Hosler J,  Jones AE. Early alterations in platelet mitochondrial function are associated with survival and organ failure in patients with septic shock. J Crit Care. 2016;31(1):63-7.

Rasmussen AM, Jakobsen R, Strøm T, Carlsson M, Dahler-Eriksen B, Toft P. More complications in patients with septic shock treated with dextran compared with crystalloids. Dan Med J. 2015;62(2).

Warmerdam M, Stolwijk F, Boogert A, Sharma M, Tetteroo L, Lucke J, et al. Initial disease severity and quality of care of emergency department sepsis patients who are older or younger than 70 years of age. PLoS One. 2017;12(9):e0185214.

Wiewel MA, de Stoppelaar SF, van Vught LA, Frencken JF, Hoogendijk AJ, Klein Klouwenberg PMC, et al. Chronic antiplatelet therapy is not associated with alterations in the presentation, outcome, or host response biomarkers during sepsis: a propensity-matched analysis. Intensive Care Med. 2016;42(3):352-360.

ProCESS Investigators, Yealy DM, Kellum JA, Huang DT, Barnato AE, Weissfeld LA, et al. A randomized trial of protocol-based care for early septic shock. N Engl J Med. 2014;370(18):1683-93.

Orde SR, Pulido JN, Masaki M, Gillespie S, Spoon JN, Kane GC, et al. Outcome prediction in sepsis: speckle tracking echocardiography based assessment of myocardial function. Crit Care. 2014;18(4):R149.

Osawa R, Wagener M, Singh N. Cytomegalovirus infection in patients with sepsis due to bloodstream infections: lower risk and better outcomes in new versus already hospitalised intensive care unit admissions. Anaesth Intensive Care. 2016;44(5):571-80.

Ostrowski SR, Haase N, Müller RB, Møller MH, Pott FC, Perner A, et al. Association between biomarkers of endothelial injury and hypocoagulability in patients with severe sepsis: a prospective study. Crit Care. 2015;19:191.

Sturgess DJ, Marwick TH, Joyce C, Jenkins C, Jones M, Masci P, et al. Prediction of hospital outcome in septic shock: a prospective comparison of tissue Doppler and cardiac biomarkers. Crit Care. 2010;14(2):R44.

Stupica D, Lusa L, Klevišar MN, Terzić S, Pirš M, Premru MM, et al. Should we consider faecal colonisation with extended-spectrum β-lactamase-producing Enterobacteriaceae in empirical therapy of community-onset sepsis? Int J Antimicrob Agents. 2017;50(4):564-571.

Stortz JA, Murphy TJ, Raymond SL, Mira JC, Ungaro R, Dirain ML, et al. Evidence for Persistent Immune Suppression in Patients Who Develop Chronic Critical Illness After Sepsis. Shock. 2018;49(3):249-258.

Stevenson EK, Rubenstein AR, Radin GT, Wiener RS, Walkey AJ. Two decades of mortality trends among patients with severe sepsis: a comparative meta-analysis*. Crit Care Med. 2014;42(3):625-31.

Sponholz C, Huse K, Kramer M, Giamarellos-Bourboulis EJ, Claus RA, Kern A, et al. Gene polymorphisms in the heme degradation pathway and outcome of severe human sepsis. Shock. 2012;38(5):459-65.

Smith SH, Perner A. Higher vs. lower fluid volume for septic shock: clinical characteristics and outcome in unselected patients in a prospective, multicenter cohort. Crit Care. 2012;16(3):R76.

Sligl WI, Milner DA Jr, Sundar S, Mphatswe W, Majumdar SR. Safety and efficacy of corticosteroids for the treatment of septic shock: A systematic review and meta-analysis. Clin Infect Dis. 2009;49(1):93-101.

Sjövall F, Alobaid AS, Wallis SC, Perner A, Lipman J, Roberts JA. Maximally effective dosing regimens of meropenem in patients with septic shock. J Antimicrob Chemother. 2018;73(1):191-198.

Sirvent JM, Ferri C, Baró A, Murcia C, Lorencio C. Fluid balance in sepsis and septic shock as a determining factor of mortality. Am J Emerg Med. 2015;33(2):186-9.

Shakoory B, Carcillo JA, Chatham WW, Amdur RL, Zhao H, Dinarello CA, et al. Interleukin-1 Receptor Blockade Is Associated With Reduced Mortality in Sepsis Patients With Features of Macrophage Activation Syndrome: Reanalysis of a Prior Phase III Trial. Crit Care Med. 2016 Feb;44(2):275-81.

Schuetz P, Birkhahn R, Sherwin R, Jones AE, Singer A, Kline JA, et al. Serial Procalcitonin Predicts Mortality in Severe Sepsis Patients: Results From the Multicenter Procalcitonin MOnitoring SEpsis (MOSES) Study. Crit Care Med. 2017;45(5):781-789.

Schnell D, Besset S, Lengliné E, Maziers N, Zafrani L, Reuter D, et al. Impact of a recent chemotherapy on the duration and intensity of the norepinephrine support during septic shock. Shock. 2013;39(2):138-43.

Scheer CS, Fuchs C, Kuhn SO, Vollmer M, Rehberg S, Friesecke S, et al. Quality Improvement Initiative for Severe Sepsis and Septic Shock Reduces 90-Day Mortality: A 7.5-Year Observational Study. Crit Care Med. 2017;45(2):241-252.

Schädler D, Pausch C, Heise D, Meier-Hellmann A, Brederlau J, Weiler N, et al. The effect of a novel extracorporeal cytokine hemoadsorption device on IL-6 elimination in septic patients: A randomized controlled trial. PLoS One. 2017;12(10):e0187015.

Savioli M, Cugno M, Polli F, Taccone P, Bellani G, Spanu P, et al. Tight glycemic control may favor fibrinolysis in patients with sepsis. Crit Care Med. 2009;37(2):424-31.

Sánchez B, Ferrer R, Suarez D, Romay E, Piacentini E, Gomà G, et al. Declining mortality due to severe sepsis and septic shock in Spanish intensive care units: A two-cohort study in 2005 and 2011. Med Intensiva. 2017;41(1):28-37.

Green JP, Berger T, Garg N, Horeczko T, Suarez A, Radeos MS, et al. Hyperlactatemia affects the association of hyperglycemia with mortality in nondiabetic adults with sepsis. Acad Emerg Med. 2012;19(11):1268-75.

Martí-Carvajal AJ, Solà I, Lathyris D, Cardona AF. Human recombinant activated protein C for severe sepsis. Cochrane Database Syst Rev. 2011 ;(4):CD004388.

Tsaganos T, Raftogiannis M, Pratikaki M, Christodoulou S, Kotanidou A, Papadomichelakis E, et al. Clarithromycin Leads to Long-Term Survival and Cost Benefit in Ventilator-Associated Pneumonia and Sepsis. Antimicrob Agents Chemother. 2016;60(6):3640-6.

Tulloch LG, Chan JD, Carlbom DJ, Kelly MJ, Dellit TH, Lynch JB. Epidemiology and Microbiology of Sepsis Syndromes in a University-Affiliated Urban Teaching Hospital and Level-1 Trauma and Burn Center. J Intensive Care Med. 2017;32(4):264-272.

Uvizl R, Adamus M, Cerny V, Dusek L, Jarkovsky J, Sramek V, et al. Patient survival, predictive factors and disease course of severe sepsis in Czech intensive care units: A multicentre, retrospective, observational study. Biomed Pap Med Fac Univ Palacky Olomouc Czech Repub. 2016 ;160(2):287-97.

Vaara ST, Hollmén M, Korhonen AM, Maksimow M, Ala-Kokko T, Salmi M, et al. Soluble CD73 in Critically Ill Septic Patients - Data from the Prospective FINNAKI Study. PLoS One. 2016;11(10):e0164420.

van Vught LA, Klein Klouwenberg PM, Spitoni C, Scicluna BP, Wiewel MA, Horn J, et al. Incidence, Risk Factors, and Attributable Mortality of Secondary Infections in the Intensive Care Unit After Admission for Sepsis. JAMA. 2016;315(14):1469-79.

Vasu TS, Cavallazzi R, Hirani A, Kaplan G, Leiby B, Marik PE. Norepinephrine or dopamine for septic shock: systematic review of randomized clinical trials. J Intensive Care Med. 2012;27(3):172-8.

Venkatesh B, Finfer S, Cohen J, Rajbhandari D, Arabi Y, Bellomo R, et al. Adjunctive Glucocorticoid Therapy in Patients with Septic Shock. N Engl J Med. 2018;378(9):797-808.

Villa G, Chelazzi C, Morettini E, Zamidei L, Valente S, Caldini AL, et al. Organ dysfunction during continuous veno-venous high cut-off hemodialysis in patients with septic acute kidney injury: A prospective observational study. PLoS One. 2017;12(2):e0172039.

Vincent JL, Marshall JC, Dellinger RP, Simonson SG, Guntupalli K, Levy MM, et al. Talactoferrin in Severe Sepsis: Results From the Phase II/III Oral tAlactoferrin in Severe sepsIS Trial. Crit Care Med. 2015;43(9):1832-8.

Vincent JL, Nelson DR, Williams MD. Is worsening multiple organ failure the cause of death in patients with severe sepsis? Crit Care Med. 2011;39(5):1050-5.

Vincent JL, Privalle CT, Singer M, Lorente JA, Boehm E, Meier-Hellmann A, et al. Multicenter, randomized, placebo-controlled phase III study of pyridoxalated hemoglobin polyoxyethylene in distributive shock (PHOENIX). Crit Care Med. 2015;43(1):57-64.

Volbeda M, Wetterslev J, Gluud C, Zijlstra JG, van der Horst IC, Keus F. Glucocorticosteroids for sepsis: systematic review with meta-analysis and trial sequential analysis. Intensive Care Med. 2015;41(7):1220-34.

Ríos-Toro JJ, Márquez-Coello M, García-Álvarez JM, Martín-Aspas A, Rivera-Fernández R, Sáez de Benito A, et al. Soluble membrane receptors, interleukin 6, procalcitonin and C reactive protein as prognostic markers in patients with severe sepsis and septic shock. PLoS One. 2017;12(4):e0175254.

Rosenqvist M, Fagerstrand E, Lanbeck P, Melander O, Åkesson P. Sepsis Alert - a triage model that reduces time to antibiotics and length of hospital stay. Infect Dis (Lond). 2017;49(7):507-513.

Rosland RG, Hagen MU, Haase N, Holst LB, Plambech M, Madsen KR, et al. Red blood cell transfusion in septic shock - clinical characteristics and outcome of unselected patients in a prospective, multicentre cohort. Scand J Trauma Resusc Emerg Med. 2014;22:14.

Guidet B, Martinet O, Boulain T, Philippart F, Poussel JF, Maizel J, et al. Assessment of hemodynamic efficacy and safety of 6% hydroxyethylstarch 130/0.4 vs. 0.9% NaCl fluid replacement in patients with severe sepsis: the CRYSTMAS study. Crit Care. 2012;16(3):R94.

Guntupalli K, Dean N, Morris PE, Bandi V, Margolis B, Rivers E, et al. A phase 2 randomized, double-blind, placebo-controlled study of the safety and efficacy of talactoferrin in patients with severe sepsis. Crit Care Med. 2013;41(3):706-16.

Han D, Shang W, Wang G, Sun L, Zhang Y, Wen H, et al. Ulinastatin- and thymosin α1-based immunomodulatory strategy for sepsis: A meta-analysis. Int Immunopharmacol. 2015 Dec;29(2):377-382.

Karampela I, Kandri E, Antonakos G, Vogiatzakis E, Christodoulatos GS, Nikolaidou A, et al. Kinetics of circulating fetuin-A may predict mortality independently from adiponectin, high molecular weight adiponectin and prognostic factors in critically ill patients with sepsis: A prospective study. J Crit Care. 2017;41:78-85.

Askim Å, Mehl A, Paulsen J, DeWan AT, Vestrheim DF, Åsvold BO et al. Epidemiology and outcome of sepsis in adult patients with Streptococcus pneumoniae infection in a Norwegian county 1993-2011: an observational study. BMC Infect Dis. 2016;16:223.

Macdonald SP, Arendts G, Fatovich DM, Brown SG. Comparison of PIRO, SOFA, and MEDS scores for predicting mortality in emergency department patients with severe sepsis and septic shock. Acad Emerg Med. 2014;21(11):1257-63.

Nakada TA, Russell JA, Boyd JH, Aguirre-Hernandez R, Thain KR, Thair SA, et al. beta2-Adrenergic receptor gene polymorphism is associated with mortality in septic shock. Am J Respir Crit Care Med. 2010;181(2):143-9.

Papanikolaou J, Makris D, Mpaka M, Palli E, Zygoulis P, Zakynthinos E. New insights into the mechanisms involved in B-type natriuretic peptide elevation and its prognostic value in septic patients. Crit Care. 2014;18(3):R94.

ARISE Investigators; ANZICS Clinical Trials Group, Peake SL, Delaney A, Bailey M, Bellomo R, et al. Goal-directed resuscitation for patients with early septic shock. N Engl J Med. 2014;371(16):1496-506.

Pedersen PB, Henriksen DP, Mikkelsen S, Lassen AT. Dispatch and prehospital transport for acute septic patients: an observational study. Scand J Trauma Resusc Emerg Med. 2017;25(1):51.

Pettilä V, Hjortrup PB, Jakob SM, Wilkman E, Perner A, Takala J. Control groups in recent septic shock trials: a systematic review. Intensive Care Med. 2016;42(12):1912-1921.

Průcha M, Zazula R, Herold I, Dostál M, Hyánek T, Bellingan G. Presence of hypogammaglobulinemia - a risk factor of mortality in patients with severe sepsis, septic shock, and SIRS. Prague Med Rep. 2013;114(4):246-57.

Quinten VM, van Meurs M, Wolffensperger AE, Ter Maaten JC, Ligtenberg JJM. Sepsis patients in the emergency department: stratification using the Clinical Impression Score, Predisposition, Infection, Response and Organ dysfunction score or quick Sequential Organ Failure Assessment score? Eur J Emerg Med. 2018;25(5):328-334.

Ranieri VM, Thompson BT, Barie PS, Dhainaut JF, Douglas IS, Finfer S, et al. Drotrecogin alfa (activated) in adults with septic shock. N Engl J Med. 2012;366(22):2055-64.

Stevenson M, Pandor A, Martyn-St James M, Rafia R, Uttley L, Stevens J, et al. Sepsis: the LightCycler SeptiFast Test MGRADE®, SepsiTest™ and IRIDICA BAC BSI assay for rapidly identifying bloodstream bacteria and fungi - a systematic review and economic evaluation. Health Technol Assess. 2016;20(46):1-246.

Statz S, Sabal G, Walborn A, Williams M, Hoppensteadt D, Mosier M, et al. Angiopoietin 2 Levels in the Risk Stratification and Mortality Outcome Prediction of Sepsis-Associated Coagulopathy. Clin Appl Thromb Hemost. 2018;24(8):1223-1233.

Simon TP, Martin L, Doemming S, Humbs A, Bruells C, Kopp R, et al. Plasma adrenomedullin in critically ill patients with sepsis after major surgery: A pilot study. J Crit Care. 2017;38:68-72.

Serpa Neto A, Veelo DP, Peireira VG, de Assunção MS, Manetta JA, Espósito DC, et al. Fluid resuscitation with hydroxyethyl starches in patients with sepsis is associated with an increased incidence of acute kidney injury and use of renal replacement therapy: a systematic review and meta-analysis of the literature. J Crit Care. 2014;29(1):185.e1-7.

Scicluna BP, van Vught LA, Zwinderman AH, Wiewel MA, Davenport EE, Burnham KL, et al. Classification of patients with sepsis according to blood genomic endotype: a prospective cohort study. Lancet Respir Med. 2017;5(10):816-826.
