# Supplementary material for: Mortality in sepsis and septic shock in Europe, North America and Australia between 2009 and 2019— results from a systematic review and meta-analysis
Source: Crit Care. 2020 May 19;24:239. doi: 10.1186/s13054-020-02950-2 (PMC7236499; doi:10.1186/s13054-020-02950-2)
Supplement: Supplementary file 3 — Additional file 3. Table of studies per endpoint and Risk of bias assessment. Table of studies shows which study was used for which endpoint in the meta-analysis. [file 13054_2020_2950_MOESM3_ESM.docx]

# Additional file 3: Table of studies per endpoint and Risk of Bias

Table S1: Septic shock 30-day mortality

| **First author** | **Titel** | **Year** | **Method for ROB Assessment** | **Risk of Bias** | **Study type** | **Country** |
| --- | --- | --- | --- | --- | --- | --- |
| Alvarez | Cost analysis of real-time polymerase chain reaction microbiological diagnosis in patients with septic shock | 2012 | ROBINS | critical | retrospective cohort study | Spain |
| Annane | Corticosteroidsin the Treatment of Severe Sepsis and Septic Shock in Adults | 2009 |  | Low | Meta-analysis | - |
| Annane | Corticosteroid treatment and intensive insulin therapy for septic shock in adults: a randomized controlled trial. | 2010 | ROB2 | low | RCT | France |
| Annane | Recombinant human activated protein C for adults with septic shock: a randomized controlled trial. | 2013 | ROB2 | low | RCT | France |
| Asfar | High versus low blood-pressure target in patients with septic shock. | 2014 | ROB2 | Some concern | RCT | France |
| Asfar | Hyperoxia and hypertonic saline in patients with septic shock (HYPERS2S): a two-by-two factorial, multicentre, randomised, clinical trial. | 2017 | ROB2 | low | RCT | France |
| Askim | Epidemiology and outcome of sepsis in adult patients with Streptococcus pneumoniae infection in a Norwegian county 1993–2011: an observational study | 2016 | ROBINS | Low | prosp. cohort study | Norway |
| Avni | Vasopressors for the Treatment of Septic Shock: Systematic Review and Meta-Analysis. | 2015 |  | Low | Meta-analysis | - |
| Balik | Propafenone for supraventricular arrhythmias in septic shock-Comparison to amiodarone and metoprolol. | 2017 | ROBINS | Moderate | retrospective cohort study | Czech Republic |
| Behnes | Diagnostic and prognostic utility of soluble CD 14 subtype (presepsin) for severe sepsis and septic shock during the first week of intensive care treatment. | 2014 | ROBINS | Moderate | retrospective cohort study | Germany |
| Bernard | Evaluating the efficacy and safety of two doses of the polyclonal anti-tumor necrosis factor-alpha fragment antibody AZD9773 in adult patients with severe sepsis and/or septic shock: randomized, double-blind, placebo-controlled phase IIb study*. | 2014 | ROB2 | low | RCT | Multi-country |
| Biyikli | Effect of platelet-lymphocyte ratio and lactate levels obtained on mortality with sepsis and septic shock. | 2018 | ROBINS | Moderate | retrospective cohort study | Turkey |
| Bloos | Effect of a multifaceted educational intervention for anti-infectious measures on sepsis mortality: a cluster randomized trial. | 2017 | ROB2 | Some concern | RCT | Germany |
| Bloos | Impact of compliance with infection management guidelines on outcome in patients with severe sepsis: a prospective observational multi-center study. | 2014 | ROBINS | Low | prosp. cohort study | Germany |
| Bloos | Effect of Sodium Selenite Administration and Procalcitonin-Guided Therapy on Mortality in Patients With Severe Sepsis or Septic Shock: A Randomized Clinical Trial. | 2016 | ROB2 | Low | RCT | Germany |
| Borthwick | High-volume haemofiltration for sepsis in adults. | 2017 |  | Low | Meta-analysis | - |
| Boulain | Prevalence of low central venous oxygen saturation in the first hours of intensive care unit admission and associated mortality in septic shock patients: a prospective multicentre study. | 2014 | ROBINS | Moderate | prosp. cohort study | France |
| Boulain | Dopamine therapy in septic shock: detrimental effect on survival? | 2009 | ROBINS | Moderate | retrospective cohort study | France |
| Brown | Coefficient of Variation of Coarsely Sampled Heart Rate is Associated With Early Vasopressor Independence in Severe Sepsis and Septic Shock. | 2015 | ROBINS | Moderate | retrospective cohort study | US |
| Brunkhorst | Effect of empirical treatment with moxifloxacin and meropenem vs meropenem on sepsis-related organ dysfunction in patients with severe sepsis: a randomized trial. | 2012 | ROB2 | low | RCT | Germany |
| Caironi | Albumin replacement in patients with severe sepsis or septic shock. | 2014 | ROB2 | Low | RCT | Italy |
| Castegren | Initial levels of organ failure, microbial findings and mortality in intensive care-treated primary, secondary and tertiary sepsis. | 2015 | ROBINS | Moderate | retrospective cohort study | Sweden |
| Chan | Etomidate is associated with mortality and adrenal insufficiency in sepsis: a meta-analysis*. | 2012 |  | Low | meta-analysis | - |
| Clark | High-volume hemofiltration for septic acute kidney injury: a systematic review and meta-analysis | 2014 |  | Low | Meta-analysis | - |
| Coccolini | Early goal-directed treatment versus standard care in management of early septic shock: Meta-analysis of randomized trials. | 2016 |  | Low | Meta-analysis | - |
| Coen | Towards a less invasive approach to the early goal-directed treatment of septic shock in the ED. | 2014 | ROBINS | Moderate | prosp. cohort study | Italy |
| Contou | Septic shock with no diagnosis at 24 hours: a pragmatic multicenter prospective cohort study. | 2016 | ROBINS | Moderate | prosp. cohort study | France |
| Cruz | Early use of polymyxin B hemoperfusion in abdominal septic shock: the EUPHAS randomized controlled trial. | 2009 | ROB2 | low | RCT | Italy |
| De La Torre-Prados | Mid-regional pro-adrenomedullin as prognostic biomarker in septic shock. | 2016 | ROBINS | Moderate | prosp. cohort study | Spain |
| Dellinger | Effect of Targeted Polymyxin B Hemoperfusion on 28-Day Mortality in Patients With Septic Shock and Elevated Endotoxin Level: The EUPHRATES Randomized Clinical Trial. | 2018 | ROB2 | Some concern | RCT | North America |
| Ferraris | Mottling score and skin temperature in septic shock: Relation and impact on prognosis in ICU. | 2018 | ROBINS | Moderate | prosp. cohort study | France |
| Fisher | Elevated Plasma Angiopoietin-2 Levels Are Associated With Fluid Overload, Organ Dysfunction, and Mortality in Human Septic Shock. | 2016 | ROBINS | Moderate | RCT | Multi-country |
| Fjell | Cytokines and signaling molecules predict clinical outcomes in sepsis. | 2013 | ROBINS | Serious | RCT | Multi-country |
| Garcia-Lopez | Impact of the implementation of a Sepsis Code hospital protocol in antibiotic prescription and clinical outcomes in an intensive care unit. | 2017 | ROBINS | Moderate | retrospective cohort study | Spain |
| Gordon | The effects of vasopressin on acute kidney injury in septic shock. | 2010 | ROB2 | Low | RCT | Multi-country |
| Guntupalli | A phase 2 randomized, double-blind, placebo-controlled study of the safety and efficacy of talactoferrin in patients with severe sepsis. | 2013 | ROB2 | Some concern | RCT | US |
| Hammond | Prospective Open-label Trial of Early Concomitant Vasopressin and Norepinephrine Therapy versus Initial Norepinephrine Monotherapy in Septic Shock. | 2018 | ROBINS | Modereate | prosp. cohort study | US |
| Jung | Effects of etomidate on complications related to intubation and on mortality in septic shock patients treated with hydrocortisone: a propensity score analysis. | 2012 | ROBINS | Serious | retrospective cohort study | France |
| Karampela | Kinetics of circulating fetuin-A may predict mortality independently from adiponectin, high molecular weight adiponectin and prognostic factors in critically ill patients with sepsis: A prospective study. | 2017 | ROBINS | Moderate | prosp. cohort study | Greece |
| Kumar | Early combination antibiotic therapy yields improved survival compared with monotherapy in septic shock: a propensity-matched analysis. | 2010 | ROBINS | Moderate | Meta-analysis | Multi-country |
| Lai | An updated meta-analysis to understand the variable efficacy of drotrecogin alfa (activated) in severe sepsis and septic shock. | 2013 |  | Low | Meta-analysis | - |
| Laribi | CT-proAVP is not a good predictor of a vasopressor need in septic shock. | 2015 | ROBINS | Critical | prosp. cohort study | Belgium |
| Lorente | Higher platelet cytochrome oxidase specific activity in surviving than in non-surviving septic patients. | 2014 | ROBINS | Serious | prosp. cohort study | Spain |
| Lorente | Association between serum soluble CD40 ligand levels and mortality in patients with severe sepsis. | 2011 | ROBINS | Moderate | prosp. cohort study | Spain |
| Macdonald | ﻿Comparison of PIRO, SOFA, and MEDS scores for predicting mortality in emergency department patients with severe sepsis and septic shock. | 2014 | ROBINS | Serious | prosp. cohort study | Australia |
| Monti | Rescue therapy with polymyxin B hemoperfusion in high-dose vasopressor therapy refractory septic shock. | 2015 | ROBINS | Serious | retrospective cohort study | Italy |
| Nakada | ﻿beta2-Adrenergic receptor gene polymorphism is associated with mortality in septic shock. | 2010 | ROBINS | Serious | other | Canada |
| Nardi | Targeting skeletal muscle tissue oxygenation (StO2) in adults with severe sepsis and septic shock: a randomised controlled trial (OTO-StS Study). | 2018 | ROB2 | Low | RCT | Multi-country |
| Nikitas | ﻿Elevated adipose tissue lactate to pyruvate (L/P) ratio predicts poor outcome in critically ill patients with septic shock: a microdialysis study. | 2013 | ROBINS | Serious | prosp. cohort study | Greece |
| Papanikolaou | New insights into the mechanisms involved in B-type natriuretic peptide elevation and its prognostic value in septic patients | 2014 | ROBINS | Moderate | prosp. cohort study | Greece |
| Parsons | Red blood cell transfusion and outcomes in patients with acute lung injury, sepsis and shock. | 2011 | ROBINS | Moderate | retrospective cohort study | North America |
| Patel | Efficacy and safety of dopamine versus norepinephrine in the management of septic shock. | 2010 | ROB2 | Some concern | RCT | US |
| Payen | Is thenar tissue hemoglobin oxygen saturation in septic shock related to macrohemodynamic variables and outcome? | 2009 | ROBINS | Moderate | prosp. cohort study | France |
| Payen | Impact of continuous venovenous hemofiltration on organ failure during the early phase of severe sepsis: a randomized controlled trial. | 2009 | ROB2 | Some concern | RCT | France |
| Peake | Goal-directed resuscitation for patients with early septic shock. | 2014 | ROB2 | Low | RCT | Multi-country |
| Pedersen | Dispatch and prehospital transport for acute septic patients: an observational study. | 2017 | ROBINS | Low | retrospective cohort study | Denmark |
| Perner | Hydroxyethyl starch 130/0.42 versus Ringer's acetate in severe sepsis. | 2012 | ROB2 | Low | RCT | Skandinavia |
| Pettila | Control groups in recent septic shock trials: a systematic review. | 2016 |  | Low | Meta-analysis | - |
| Prucha | Presence of hypogammaglobulinemia - a risk factor of mortality in patients with severe sepsis, septic shock, and SIRS. | 2013 | ROBINS | Serious | retrospective cohort study | Czech Republic |
| Puskarich | Preliminary safety and efficacy of L-carnitine infusion for the treatment of vasopressor-dependent septic shock: a randomized control trial. | 2014 | ROB2 | Low | RCT | US |
| Puskarich | Early alterations in platelet mitochondrial function are associated with survival and organ failure in patients with septic shock. | 2016 | ROBINS | Low | prosp. cohort study | US |
| Ranieri | Drotrecogin alfa (activated) in adults with septic shock. | 2012 | ROB2 | Low | RCT | Europe, North and South America, Australia, New Zealand, India |
| Orde | ﻿Outcome prediction in sepsis: speckle tracking echocardiography based assessment of myocardial function. | 2014 | ROBINS | Low | prosp. cohort study | US |
| Sturgess | Prediction of hospital outcome in septic shock: a prospective comparison of tissue Doppler and cardiac biomarkers. | 2010 | ROBINS | Moderate | prosp. cohort study | Australia |
| Stupica | Should we consider faecal colonisation with extended-spectrum beta-lactamase-producing Enterobacteriaceae in empirical therapy of community-onset sepsis? | 2017 | ROBINS | Moderate | retrospective cohort study | Slovenia |
| Statz | Angiopoietin 2 Levels in the Risk Stratification and Mortality Outcome Prediction of Sepsis-Associated Coagulopathy. | 2018 | ROBINS | Serious | prosp. cohort study | US |
| Smith | Higher vs. lower fluid volume for septic shock: clinical characteristics and outcome in unselected patients in a prospective, multicenter cohort. | 2012 | ROBINS | Serious | prosp. cohort study | Denmark |
| Sligl | Safety and efficacy of corticosteroids for the treatment of septic shock: A systematic review and meta-analysis. | 2009 |  | Low | Meta-analysis | - |
| Sirvent | Fluid balance in sepsis and septic shock as a determining factor of mortality. | 2015 | ROBINS | Moderate | prosp. cohort study | Spain |
| Shakoory | Interleukin-1 Receptor Blockade Is Associated With Reduced Mortality in Sepsis Patients With Features of Macrophage Activation Syndrome: Reanalysis of a Prior Phase III Trial. | 2016 | ROB2 | Low | RCT | North America, Europe |
| Schnell | Impact of a recent chemotherapy on the duration and intensity of the norepinephrine support during septic shock. | 2013 | ROBINS | Serious | retrospective cohort study | France |
| Scheer | Quality Improvement Initiative for Severe Sepsis and Septic Shock Reduces 90-Day Mortality: A 7.5-Year Observational Study. | 2017 | ROBINS | Moderate | prosp. cohort study | Germany |
| Sanchez | Declining mortality due to severe sepsis and septic shock in Spanish intensive care units: A two-cohort study in 2005 and 2011. | 2017 | ROBINS | Moderate | prosp. cohort study | Spain |
| Uvizl | Patient survival, predictive factors and disease course of severe sepsis in Czech intensive care units: A multicentre, retrospective, observational study. | 2015 | ROBINS | Moderate | RCT | Czech Republic |
| Vasu | Norepinephrine or dopamine for septic shock: systematic review of randomized clinical trials. | 2012 |  | Low | other | - |
| Vincent | Is worsening multiple organ failure the cause of death in patients with severe sepsis? | 2011 | ROBINS | Moderate | other | Multi-country |
| Vincent | Multicenter, randomized, placebo-controlled phase III study of pyridoxalated hemoglobin polyoxyethylene in distributive shock (PHOENIX). | 2015 | ROB2 | Low | RCT | Multi-country |
| Rios-Toro | Soluble membrane receptors, interleukin 6, procalcitonin and C reactive protein as prognostic markers in patients with severe sepsis and septic shock. | 2017 | ROBINS | Low | prosp. cohort study | Spain |
| Rosland | Red blood cell transfusion in septic shock - clinical characteristics and outcome of unselected patients in a prospective, multicentre cohort. | 2014 | ROBINS | Low | prosp. cohort study | Denmark |

Table S2: Septic shock 90-day mortality

| Author | Titel | Year | Method for ROB Assessment | Risk of Bias | Study type | Country |
| --- | --- | --- | --- | --- | --- | --- |
| Angus | A systematic review and meta-analysis of early goal-directed therapy for septic shock: the ARISE, ProCESS and ProMISe Investigators | 2015 |  | Low | Meta-analysis | - |
| Annane | Corticosteroid treatment and intensive insulin therapy for septic shock in adults: a randomized controlled trial. | 2010 | ROB2 | low | RCT | France |
| Annane | Hydrocortisone plus Fludrocortisone for Adults with Septic Shock. | 2018 | ROB2 | Low | RCT | France |
| Annane | Recombinant human activated protein C for adults with septic shock: a randomized controlled trial. | 2013 | ROB2 | Low | RCT | France |
| Asfar | High versus low blood-pressure target in patients with septic shock. | 2014 | ROB2 | Some concern | RCT | France |
| Asfar | Hyperoxia and hypertonic saline in patients with septic shock (HYPERS2S): a two-by-two factorial, multicentre, randomised, clinical trial. | 2017 | ROB2 | low | RCT | France |
| Bloos | Effect of Sodium Selenite Administration and Procalcitonin-Guided Therapy on Mortality in Patients With Severe Sepsis or Septic Shock: A Randomized Clinical Trial. | 2016 | ROB2 | Low | RCT | Germany |
| Brunkhorst | Effect of empirical treatment with moxifloxacin and meropenem vs meropenem on sepsis-related organ dysfunction in patients with severe sepsis: a randomized trial. | 2012 | ROB2 | low | RCT | Germany |
| Caironi | Albumin replacement in patients with severe sepsis or septic shock. | 2014 | ROB2 | Low | RCT | Italy |
| Coccolini | Early goal-directed treatment versus standard care in management of early septic shock: Meta-analysis of randomized trials. | 2016 |  | Low | Meta-analysis | - |
| Coen | Towards a less invasive approach to the early goal-directed treatment of septic shock in the ED. | 2014 | ROBINS | Moderate | prosp. cohort study | Italy |
| Dahl | Variability in targeted arterial oxygenation levels in patients with severe sepsis or septic shock | 2015 | ROBINS | Low | retrospective cohort study | Scandinavia |
| Fisher | Elevated Plasma Angiopoietin-2 Levels Are Associated With Fluid Overload, Organ Dysfunction, and Mortality in Human Septic Shock. | 2016 | ROBINS | Moderate | RCT | - |
| Fjell | Cytokines and signaling molecules predict clinical outcomes in sepsis. | 2013 | ROBINS | Serious | RCT | - |
| Guntupalli | A phase 2 randomized, double-blind, placebo-controlled study of the safety and efficacy of talactoferrin in patients with severe sepsis. | 2013 | ROB2 | Some concern | RCT | US |
| Hjortrup | Restricting volumes of resuscitation fluid in adults with septic shock after initial management: the CLASSIC randomised, parallel-group, multicentre feasibility trial. | 2016 | ROB2 | Low | RCT | Scandinavia |
| Holst | Lower versus higher hemoglobin threshold for transfusion in septic shock. | 2014 | ROB2 | Low | RCT | Scandinavia |
| Joannes-Boyau | High-volume versus standard-volume haemofiltration for septic shock patients with acute kidney injury (IVOIRE study): a multicentre randomized controlled trial. | 2013 | ROB2 | Low | RCT | - |
| Mouncey | Protocolised Management In Sepsis (ProMISe): a multicentre, randomised controlled trial of the clinical and cost-effectiveness of early protocolised resuscitation for emerging septic shock | 2015 | ROB2 | Low | RCT | UK |
| Peake | Goal-directed resuscitation for patients with early septic shock. | 2014 | ROB2 | Low | RCT | Mulit-country |
| Perner | Hydroxyethyl starch 130/0.42 versus Ringer's acetate in severe sepsis. | 2012 | ROB2 | Low | RCT | Skandinavia |
| Pettila | Control groups in recent septic shock trials: a systematic review. | 2016 |  | Low | Meta-analysis | - |
| Poukkanen | Variation in the use of renal replacement therapy in patients with septic shock: a substudy of the prospective multicenter observational FINNAKI study. | 2014 | ROBINS | Low | prosp. cohort study | Finland |
| Puskarich | Preliminary safety and efficacy of L-carnitine infusion for the treatment of vasopressor-dependent septic shock: a randomized control trial. | 2014 | ROB2 | Low | RCT | US |
| Ranieri | Drotrecogin alfa (activated) in adults with septic shock. | 2012 | ROB2 | low | RCT | Multi-country |
| Rasmussen | More complications in patients with septic shock treated with dextran compared with crystalloids. | 2015 | ROBINS | Moderate | retrospective cohort study | Not reported |
| Warmerdam | Initial disease severity and quality of care of emergency department sepsis patients who are older or younger than 70 years of age | 2017 | ROBINS | Moderate | prosp. cohort study | Netherlands |
| Yealy | A Randomized Trial of Protocol-Based Care for Early Septic Shock | 2014 | ROB2 | Low | RCT | US |
| Smith | Higher vs. lower fluid volume for septic shock: clinical characteristics and outcome in unselected patients in a prospective, multicenter cohort. | 2012 | ROBINS | Serious | prosp. cohort study | Denmark |
| Sjovall | Maximally effective dosing regimens of meropenem in patients with septic shock. | 2018 | ROBINS | Moderate | prosp. cohort study | Denmark |
| Scheer | Quality Improvement Initiative for Severe Sepsis and Septic Shock Reduces 90-Day Mortality: A 7.5-Year Observational Study. | 2017 | ROBINS | Moderate | prosp. cohort study | Germany |
| Schadler | The effect of a novel extracorporeal cytokine hemoadsorption device on IL-6 elimination in septic patients: A randomized controlled trial. | 2017 | ROB2 | Some concern | RCT | Germany |
| Uvizl | Patient survival, predictive factors and disease course of severe sepsis in Czech intensive care units: A multicentre, retrospective, observational study. | 2015 | ROBINS | Moderate | RCT | Czech Republic |
| Vaara | Soluble CD73 in Critically Ill Septic Patients - Data from the Prospective FINNAKI Study. | 2016 | ROBINS | Moderate | other | Finland |
| Venkatesh | Adjunctive Glucocorticoid Therapy in Patients with Septic Shock. | 2018 | ROB2 | Low | RCT | Australia, United Kingdom, New Zealand, Saudi Arabia, Denmark |
| Villa | Organ dysfunction during continuous veno-venous high cut-off hemodialysis in patients with septic acute kidney injury: A prospective observational study. | 2017 | ROBINS | Moderate | prosp. cohort study | Italy |
| Rosland | Red blood cell transfusion in septic shock - clinical characteristics and outcome of unselected patients in a prospective, multicentre cohort. | 2014 | ROBINS | Low | prosp. cohort study | Denmark |

Table S3: Sepsis 30-day mortality

| Author | Titel | Year | Method for ROB Assessment | Risk of Bias | Study type | Country |
| --- | --- | --- | --- | --- | --- | --- |
| Alhazzani | The Effect of Selenium Therapy on Mortality ¡n Patients With Sepsis Syndrome: A Systematic Review and Meta-Analysis of Randomized Controlled Trials | 2013 |  | Low | Meta-analysis | - |
| Annane | Corticosteroids for treating sepsis (Review) | 2018 |  | Low | Meta-analysis | - |
| Askim | Epidemiology and outcome of sepsis in adult patients with Streptococcus pneumoniae infection in a Norwegian county 1993–2011: an observational study | 2016 | ROBINS | Low | prosp. cohort study | Norway |
| Askim | Poor performance of quick-SOFA (qSOFA) score in predicting severe sepsis and mortality - a prospective study of patients admitted with infection to the emergency department. | 2017 | ROBINS | Low | prosp. cohort study | Norway |
| Boyd | Increased Plasma PCSK9 Levels Are Associated with Reduced Endotoxin Clearance and the Development of Acute Organ Failures during Sepsis. | 2016 | ROBINS | Moderate | prosp. cohort study | Canada |
| Contenti | Effectiveness of arterial, venous, and capillary blood lactate as a sepsis triage tool in ED patients. | 2015 | ROBINS | Moderate | prosp. cohort study | France |
| Davies | The effect of sepsis and septic shock on the viscoelastic properties of clot quality and mass using rotational thromboelastometry: A prospective observational study | 2018 | ROBINS | Moderate | prosp. cohort study | Wales |
| De Pablo | Circulating sICAM-1 and sE-Selectin as biomarker of infection and prognosis in patients with systemic inflammatory response syndrome. | 2013 | ROBINS | Moderate | prosp. cohort study | Spain |
| Dettmer | The impact of serial lactate monitoring on emergency department resuscitation interventions and clinical outcomes in severe sepsis and septic shock: an observational cohort study. | 2015 | ROBINS | Moderate | retrospective cohort study | not reported |
| Drewry | Comparison of monocyte human leukocyte antigen-DR expression and stimulated tumor necrosis factor alpha production as outcome predictors in severe sepsis: a prospective observational study. | 2016 | ROBINS | Serious | prosp. cohort study | - |
| Dupuis | Effect of Transfusion on Mortality and Other Adverse Events Among Critically Ill Septic Patients: An Observational Study Using a Marginal Structural Cox Model. | 2017 | ROBINS | Moderate | retrospective cohort study | France |
| Dwivedi | Prognostic utility and characterization of cell-free DNA in patients with severe sepsis. | 2012 | ROBINS | Moderate | retrospective cohort study | Canada |
| Elke | Enteral nutrition is associated with improved outcome in patients with severe sepsis. A secondary analysis of the VISEP trial. | 2013 | ROBINS | Low | retrospective cohort study | Germany |
| Finfer | Impact of albumin compared to saline on organ function and mortality of patients with severe sepsis. | 2011 | ROB2 | Low | RCT | multi-country |
| Gaulton | The effect of obesity on clinical outcomes in presumed sepsis: a retrospective cohort study. | 2014 | ROBINS | Serious | retrospective cohort study | US |
| Giamarellos-Bourboulis | Effect of clarithromycin in patients with suspected Gram-negative sepsis: results of a randomized controlled trial. | 2014 | ROB2 | Low | RCT | Greece |
| Gonzalez-Padilla | Gentamicin therapy for sepsis due to carbapenem-resistant and colistin-resistant Klebsiella pneumoniae. | 2015 | ROBINS | Serious | retrospective cohort study | Spain |
| Gordon | Effect of Early Vasopressin vs Norepinephrine on Kidney Failure in Patients With Septic Shock: The VANISH Randomized Clinical Trial. | 2016 | ROB2 | Low | RCT | UK |
| Guidet | Assessment of hemodynamic efficacy and safety of 6% hydroxyethylstarch 130/0.4 vs. 0.9% NaCl fluid replacement in patients with severe sepsis: the CRYSTMAS study. | 2012 | ROB2 | Low | RCT | multi-country |
| Guirgis | HDL inflammatory index correlates with and predicts severity of organ failure in patients with sepsis and septic shock. | 2018 | ROBINS | Moderate | prosp. cohort study | US |
| Guntupalli | A phase 2 randomized, double-blind, placebo-controlled study of the safety and efficacy of talactoferrin in patients with severe sepsis. | 2013 | ROB2 | Some concern | RCT | US |
| Han | Ulinastatin- and thymosin alpha1-based immunomodulatory strategy for sepsis: A meta-analysis. | 2015 |  | Low | Meta-analysis | - |
| Hanzelka | Implementation of modified early-goal directed therapy for sepsis in the emergency center of a comprehensive cancer center. | 2013 | ROBINS | Moderate | retrospective cohort study | US |
| Hazzard | Coupled plasma haemofiltration filtration in severe sepsis: systematic review and meta-analysis. | 2015 |  | Low | Meta-analysis | - |
| Heemskerk | Alkaline phosphatase treatment improves renal function in severe sepsis or septic shock patients. | 2009 | ROB2 | Low | RCT | Netherlands |
| Herran-Monge | Mortality Reduction and Long-Term Compliance with Surviving Sepsis Campaign: A Nationwide Multicenter Study. | 2016 | ROBINS | Moderate | prosp. cohort study | Spain |
| Igonin | C1-esterase inhibitor infusion increases survival rates for patients with sepsis*. | 2012 | ROB2 | High | RCT | Russia |
| Jerwood | A pilot clinical trial to evaluate a novel time-to-positivity assay to measure the effectiveness of antibiotic therapy for septic patients in intensive care. | 2012 | ROBINS | Serious | prosp. cohort study | UK |
| Jouffroy | Triage of Septic Patients Using qSOFA Criteria at the SAMU Regulation: A Retrospective Analysis. | 2018 | ROBINS | Critical | retrospective cohort study | France |
| Karampela | Kinetics of circulating fetuin-A may predict mortality independently from adiponectin, high molecular weight adiponectin and prognostic factors in critically ill patients with sepsis: A prospective study. | 2017 | ROBINS | Moderate | prosp. cohort study | Greece |
| Keep | National early warning score at Emergency Department triage may allow earlier identification of patients with severe sepsis and septic shock: a retrospective observational study. | 2016 | ROBINS | Serious | retrospective cohort study | UK |
| Keh | Effect of Hydrocortisone on Development of Shock Among Patients With Severe Sepsis: The HYPRESS Randomized Clinical Trial. | 2016 | ROB2 | Low | RCT | Germany |
| Koch | Regulation and prognostic relevance of serum ghrelin concentrations in critical illness and sepsis. | 2010 | ROBINS | Low | prosp. cohort study | Germany |
| Kristof | Anaemia requiring red blood cell transfusion is associated with unfavourable 90-day survival in surgical patients with sepsis. | 2018 | ROBINS | Moderate | prosp. cohort study | Germany |
| Kruger | A multicenter randomized trial of atorvastatin therapy in intensive care patients with severe sepsis. | 2013 | ROB2 | Low | RCT | multi-country |
| Kuipers | Incidence, risk factors and outcomes of new-onset atrial fibrillation in patients with sepsis: a systematic review. | 2014 |  | Low | other | - |
| Leaf | Randomized controlled trial of calcitriol in severe sepsis. | 2014 | ROB2 | Low | RCT | US |
| Li | Thymosin alpha1 based immunomodulatory therapy for sepsis: a systematic review and meta-analysis. | 2015 |  | Low | Meta-analysis | - |
| Lopez-Mestanza | Clinical factors influencing mortality risk in hospital-acquired sepsis. | 2018 | ROBINS | Moderate | retrospective cohort study | Spain |
| Lorente | Serum melatonin levels are associated with mortality in severe septic patients. | 2015 | ROBINS | Moderate | prosp. cohort study | Spain |
| Lorente | Sustained high serum malondialdehyde levels are associated with severity and mortality in septic patients. | 2013 | ROBINS | Serious | prosp. cohort study | Spain |
| Lorente | Sustained high plasma plasminogen activator inhibitor-1 levels are associated with severity and mortality in septic patients. | 2014 | ROBINS | Moderate | prosp. cohort study | Spain |
| Lorente | Serum levels of caspase-cleaved cytokeratin-18 and mortality are associated in severe septic patients: pilot study. | 2014 | ROBINS | Serious | prosp. cohort study | Spain |
| Lorente | Septic patients with mitochondrial DNA haplogroup JT have higher respiratory complex IV activity and survival rate. | 2016 | ROBINS | Moderate | prosp. cohort study | Spain |
| Lorente | Association of sepsis-related mortality with early increase of TIMP-1/MMP-9 ratio. | 2014 | ROBINS | Moderate | prosp. cohort study | Spain |
| Lv | Anti-TNF-alpha therapy for patients with sepsis: a systematic meta-analysis. | 2014 |  | Low | Meta-analysis | multi-country |
| Macdonald | ﻿Comparison of PIRO, SOFA, and MEDS scores for predicting mortality in emergency department patients with severe sepsis and septic shock. | 2014 | ROBINS | Serious | prosp. cohort study | Australia |
| Mansur | Impact of statin therapy on mortality in patients with sepsis-associated acute respiratory distress syndrome (ARDS) depends on ARDS severity: a prospective observational cohort study | 2015 | ROBINS | Low | prosp. cohort study | Germany |
| Mansur | Late mortality after sepsis: propensity matched cohort study. | 2015 | ROBINS | Low | prosp. cohort study | Germany |
| Nejat | ﻿Urinary cystatin C is diagnostic of acute kidney injury and sepsis, and predicts mortality in the intensive care unit. | 2010 | ROBINS | Serious | other | New Zealand |
| Nowak | ﻿Noninvasive hemodynamic monitoring in emergency patients with suspected heart failure, sepsis and stroke: the PREMIUM registry. | 2014 | ROBINS | Moderate | prosp. cohort study | US |
| Pandharipande | Effect of dexmedetomidine versus lorazepam on outcome in patients with sepsis: an a priori-designed analysis of the MENDS randomized controlled trial. | 2014 | ROB2 | Low | RCT | US |
| Pedersen | Dispatch and prehospital transport for acute septic patients: an observational study. | 2017 | ROBINS | Low | retrospective cohort study | Denmark |
| Prescott | Late mortality after sepsis: propensity matched cohort study. | 2016 | ROBINS | Moderate | retrospective cohort study | US |
| Prucha | Presence of hypogammaglobulinemia - a risk factor of mortality in patients with severe sepsis, septic shock, and SIRS. | 2013 | ROBINS | Serious | retrospective cohort study | Czech Republic |
| Quinten | Sepsis patients in the emergency department: stratification using the Clinical Impression Score, Predisposition, Infection, Response and Organ dysfunction score or quick Sequential Organ Failure Assessment score? | 2018 | ROBINS | Moderate | prosp. cohort study | Netherlands |
| Osawa | Cytomegalovirus infection in patients with sepsis due to bloodstream infections: lower risk and better outcomes in new versus already hospitalised intensive care unit admissions. | 2016 | ROBINS | Moderate | prosp. cohort study | US |
| Ostrowski | Association between biomarkers of endothelial injury and hypocoagulability in patients with severe sepsis: a prospective study | 2015 | ROBINS | Moderate | other | Denmark |
| Stortz | Evidence for Persistent Immune Suppression in Patients Who Develop Chronic Critical Illness After Sepsis. | 2018 | ROBINS | Serious | prosp. cohort study | US |
| Stevenson | Sepsis: the LightCycler SeptiFast Test MGRADE(R), SepsiTest and IRIDICA BAC BSI assay for rapidly identifying bloodstream bacteria and fungi - a systematic review and economic evaluation. | 2016 |  | Low | retrospective cohort study | 0 |
| Stevenson | Two decades of mortality trends among patients with severe sepsis: a comparative meta-analysis*. | 2014 |  | Low | Meta-analysis | - |
| Statz | Angiopoietin 2 Levels in the Risk Stratification and Mortality Outcome Prediction of Sepsis-Associated Coagulopathy. | 2018 | ROBINS | Serious | prosp. cohort study | multi-country |
| Sponholz | Gene polymorphisms in the heme degradation pathway and outcome of severe human sepsis. | 2012 | ROBINS | Serious | RCT | Germany |
| Simon | Plasma adrenomedullin in critically ill patients with sepsis after major surgery: A pilot study. | 2017 | ROBINS | Moderate | prosp. cohort study | multi-country |
| Serpa | Fluid resuscitation with hydroxyethyl starches in patients with sepsis is associated with an increased incidence of acute kidney injury and use of renal replacement therapy: a systematic review and meta-analysis of the literature. | 2014 |  | Low | Meta-analysis | - |
| Scicluna | Classification of patients with sepsis according to blood genomic endotype: a prospective cohort study. | 2017 | ROBINS | Moderate | prosp. cohort study | multi-country |
| Schuetz | Serial Procalcitonin Predicts Mortality in Severe Sepsis Patients: Results From the Multicenter Procalcitonin MOnitoring SEpsis (MOSES) Study. | 2017 | ROBINS | Low | prosp. cohort study | US |
| Green | Hyperlactatemia affects the association of hyperglycemia with mortality in nondiabetic adults with sepsis. | 2012 | ROBINS | Moderate | retrospective cohort study | US |
| Marti-Carvajal | Human recombinant activated protein C for severe sepsis. | 2011 |  | Low | Meta-analysis | - |
| van Vught | ﻿Incidence, Risk Factors, and Attributable Mortality of Secondary Infections in the Intensive Care Unit After Admission for Sepsis. | 2016 | ROBINS | Moderate | prosp. cohort study | Netherlands |
| Vincent | Talactoferrin in Severe Sepsis: Results From the Phase II/III Oral tAlactoferrin in Severe sepsIS Trial. | 2015 | ROB2 | Low | RCT | North America, Europe group 1 [Belgium, Denmark, Germany, The Netherlands], and Europe group 2 [France, Israel, Spain, United Kingdom] |
| Rosenqvist | Sepsis Alert - a triage model that reduces time to antibiotics and length of hospital stay | 2017 | ROBINS | Modereat | retrospective cohort study | Sweden |

Table S4: Sepsis 90-day mortality

| Author | Titel | Year | Method for ROB Assessment | Risk of Bias | Study type | Country |
| --- | --- | --- | --- | --- | --- | --- |
| Dulhunty | A Multicenter Randomized Trial of Continuous versus Intermittent beta-Lactam Infusion in Severe Sepsis. | 2015 | ROB2 | Low | RCT | Australia, Asia |
| Elke | Enteral nutrition is associated with improved outcome in patients with severe sepsis. A secondary analysis of the VISEP trial. | 2013 | ROBINS | Moderate | retrospective cohort study | Germany |
| Guidet | Assessment of hemodynamic efficacy and safety of 6% hydroxyethylstarch 130/0.4 vs. 0.9% NaCl fluid replacement in patients with severe sepsis: the CRYSTMAS study. | 2012 | ROB2 | Low | RCT | multi-country |
| Guntupalli | A phase 2 randomized, double-blind, placebo-controlled study of the safety and efficacy of talactoferrin in patients with severe sepsis. | 2013 | ROB2 | Some oncern | RCT | US |
| Han | Ulinastatin- and thymosin alpha1-based immunomodulatory strategy for sepsis: A meta-analysis. | 2015 |  | Low | Meta-analysis | - |
| Johansen | Profound endothelial damage predicts impending organ failure and death in sepsis. | 2015 | ROBINS | Moderate | RCT | Denmark |
| Kaffarnik | Early diagnosis of sepsis-related hepatic dysfunction and its prognostic impact on survival: a prospective study with the LiMAx test. | 2013 | ROBINS | Serious | prosp. cohort study | Germany |
| Keh | Effect of Hydrocortisone on Development of Shock Among Patients With Severe Sepsis: The HYPRESS Randomized Clinical Trial. | 2016 | ROB2 | Low | RCT | Germany |
| Khoury | The prognostic value of brain natriuretic peptide (BNP) in non-cardiac patients with sepsis, ultra-long follow-up. | 2017 | ROBINS | Moderate | prosp. cohort study | Israel |
| Koch | Regulation and prognostic relevance of serum ghrelin concentrations in critical illness and sepsis. | 2010 | ROBINS | Low | prosp. cohort study | Germany |
| Kristof | Anaemia requiring red blood cell transfusion is associated with unfavourable 90-day survival in surgical patients with sepsis. | 2018 | ROBINS | Moderate | prosp. cohort study | Germany |
| Kruger | A multicenter randomized trial of atorvastatin therapy in intensive care patients with severe sepsis. | 2013 | ROB2 | Low | RCT | multi-country |
| Mansur | ﻿Primary bacteraemia is associated with a higher mortality risk compared with pulmonary and intra-abdominal infections in patients with sepsis: a prospective observational cohort study. | 2015 | ROBINS | Moderate | prosp. cohort study | Germany |
| Prescott | Variation in Postsepsis Readmission Patterns: A Cohort Study of Veterans Affairs Beneficiaries. | 2017 | ROBINS | Moderate | retrospective cohort study | Germany |
| Prescott | Late mortality after sepsis: propensity matched cohort study. | 2016 | ROBINS | Moderate | retrospective cohort study | US |
| Wiewel | ﻿Chronic antiplatelet therapy is not associated with alterations in the presentation, outcome, or host response biomarkers during sepsis: a propensity-matched analysis. | 2016 | ROBINS | Low | other | Netherlands |
| Sponholz | Gene polymorphisms in the heme degradation pathway and outcome of severe human sepsis. | 2012 | ROBINS | Serious | RCT | Germany |
| Simon | Plasma adrenomedullin in critically ill patients with sepsis after major surgery: A pilot study. | 2017 | ROBINS | Moderate | prosp. cohort study | multi-country |
| Serpa | Fluid resuscitation with hydroxyethyl starches in patients with sepsis is associated with an increased incidence of acute kidney injury and use of renal replacement therapy: a systematic review and meta-analysis of the literature. | 2014 |  | Low | Meta-analysis | - |
| Scicluna | Classification of patients with sepsis according to blood genomic endotype: a prospective cohort study. | 2017 | ROBINS | Moderate | prosp. cohort study | multi-country |
| Savioli | Tight glycemic control may favor fibrinolysis in patients with sepsis. | 2009 | ROB2 | Some concern | RCT | Italy |
| Tsaganos | Clarithromycin Leads to Long-Term Survival and Cost Benefit in Ventilator-Associated Pneumonia and Sepsis. | 2016 | ROB2 | Low | RCT | Greece |
| Tulloch | ﻿Epidemiology and Microbiology of Sepsis Syndromes in a University-Affiliated Urban Teaching Hospital and Level-1 Trauma and Burn Center. | 2017 | ROBINS | Low | prosp. cohort study | US |
| van Vught | ﻿Incidence, Risk Factors, and Attributable Mortality of Secondary Infections in the Intensive Care Unit After Admission for Sepsis. | 2016 | ROBINS | Moderate | prosp. cohort study | Netherlands |
| Volbeda | Glucocorticosteroids for sepsis: systematic review with meta-analysis and trial sequential analysis. | 2015 |  | Low | Meta-analysis | Meta-analysis |

**Detailed risk of bias assessments for each study (RoB 2 for RCTs, ROBIN-I for observational studies) is available via the corresponding author for reasonable request.**
