# Supplementary material for: Mortality in sepsis and septic shock in Europe, North America and Australia between 2009 and 2019— results from a systematic review and meta-analysis
Source: Crit Care. 2020 May 19;24:239. doi: 10.1186/s13054-020-02950-2 (PMC7236499; doi:10.1186/s13054-020-02950-2)

# Additional file 4: Further results

Figure S1: Joinpoint estimation of 90-day sepsis mortality. Results from the estimation of pooled 90-day sepsis mortality rates per year. The Joinpoint analysis shows the timepoint when trends in mortality rates change. A trend change of 90-day sepsis mortality was found in 2011.


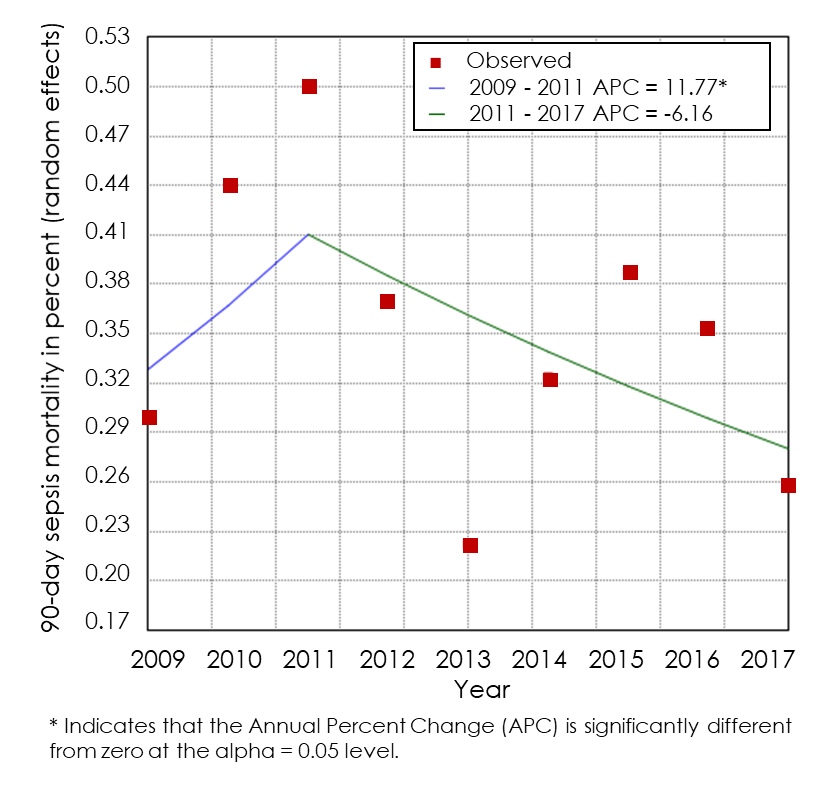


Figure S2: Linear regression of age and mortality rates. Correlation of mortality rates and age were estimated for A) 30-day septic shock B) 90-day septic shock C) 30-day sepsis and D) 90-day sepsis.


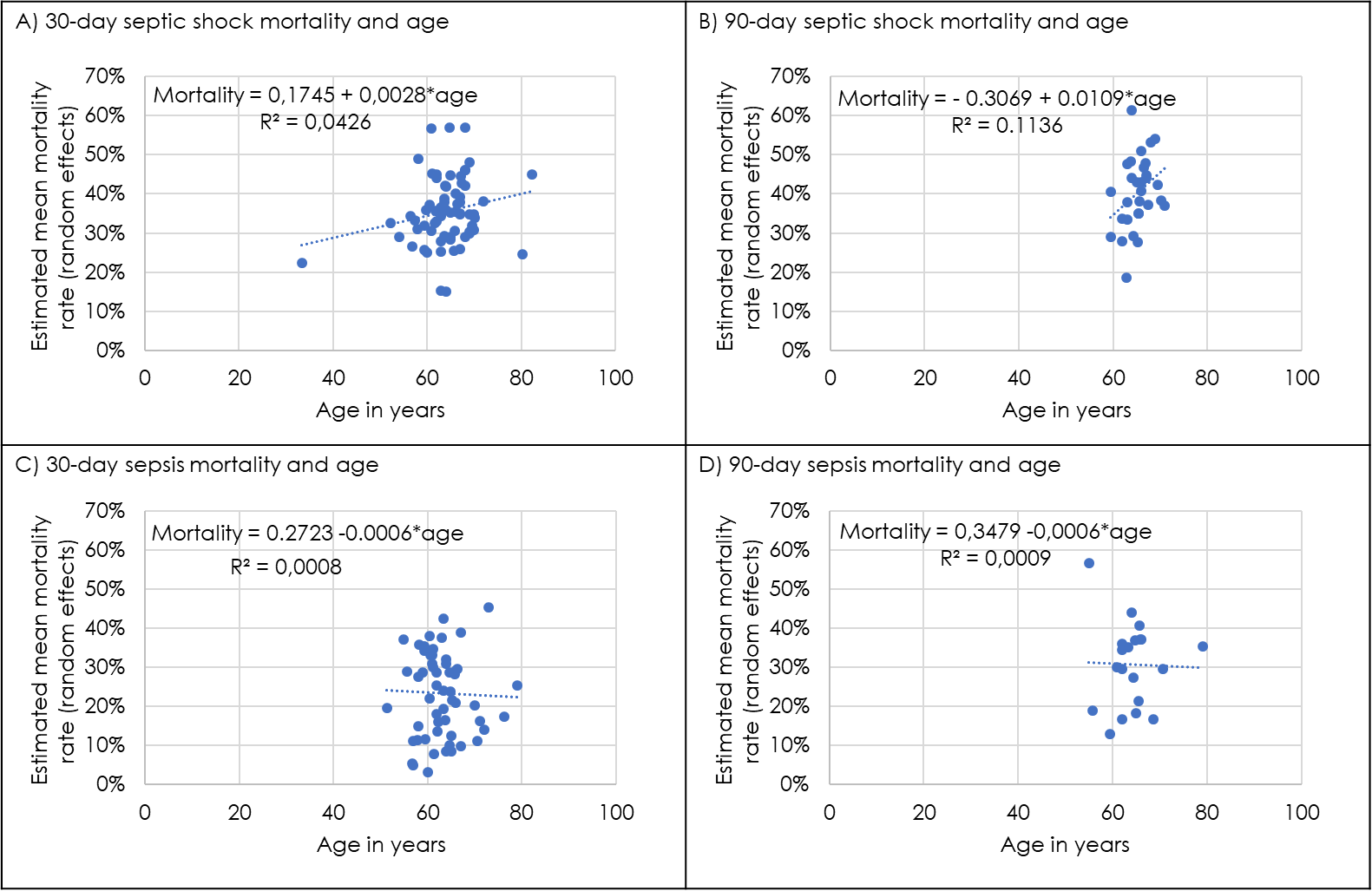

Supplement: Supplementary file 4 — Additional file 4: Further results. Figure S1. Joinpoint estimation of 90-day sepsis mortality. Results from the estimation of pooled 90-day sepsis mortality rates per year. The Joinpoint analysis shows the timepoint when trends in mortality rates change. A trend change of 90-day sepsis mortality was found in 2011. Figure S2. Linear regression of age and mortality rates. Correlation of mortality rates and age were estimated for A) 30-day septic shock B) 90-day septic shock C) 30-day sepsis and D) 90-day sepsis. [file 13054_2020_2950_MOESM4_ESM.docx]
