# Supplementary material for: Mortality in sepsis and septic shock in Europe, North America and Australia between 2009 and 2019— results from a systematic review and meta-analysis
Source: Crit Care. 2020 May 19;24:239. doi: 10.1186/s13054-020-02950-2 (PMC7236499; doi:10.1186/s13054-020-02950-2)
Supplement: Supplementary file 5 — Additional file 5. List of excluded studies after full-text screening. Table includes all studies, that were excluded after full-text review with reason for exclusion. [file 13054_2020_2950_MOESM5_ESM.docx]

# Additional file 5: List of excluded studies after full-text screening

Table S1: List of excluded studies after full-text screening

| First author | Title | Citation | Exclusion reason |
| --- | --- | --- | --- |
| Acheampong | A positive fluid balance is an independent prognostic factor in patients with sepsis | Critical Care (2015) 19:251 | No outcome of interest |
| Adamik | Endotoxin Elimination in Patients with Septic Shock: An Observation Study. | Arch Immunol Ther Exp (Warsz). 2015; 63(6): 475–483. | No outcome of interest |
| Agarwal | Continuous renal replacement therapy (CRRT) in patients with liver disease: Is circuit life different? | Journal of Hepatology 51 (2009) 504–509 | No outcome of interest |
| Ait-Oufella | Knee area tissue oxygen saturation is predictive of 14-day mortality in septic shock. | Intensive Care Med (2012) 38:976–983 | No outcome of interest |
| Ait-Oufella | Capillary refill time exploration during septic shock. | Intensive Care Med. 2014 Jul;40(7):958-64 | No outcome of interest |
| Akahoshi | Comparison of recombinant human thrombomodulin and gabexate mesylate for treatment of disseminated intravascular coagulation (DIC) with sepsis following emergent gastrointestinal surgery: a retrospective study. | Eur J Trauma Emerg Surg. 2015 Oct;41(5):531-8. | No outcome of interest |
| Akili | Prognostic importance of neutrophil-lymphocyte ratio in critically ill patients: short- and long-term outcomes | Am J Emerg Med. 2014 Dec;32(12):1476-80 | Indication |
| Al Harbi | Association between aspirin therapy and the outcome in critically ill patients: a nested cohort study. | BMC Pharmacol Toxicol. 2016 Feb 5;17:5. | No outcome of interest |
| Al Harbi | Association between statin therapy and outcomes in critically ill patients: a nested cohort study. | BMC Clin Pharmacol. 2011 Aug 6;11:12. | No outcome of interest |
| Al Harbi | Association between beta-blocker use and mortality in critically ill patients: a nested cohort study. | BMC Pharmacol Toxicol. 2018; 19: 22. | No outcome of interest |
| Al-Dorzi | Serum procalcitonin in cirrhotic patients with septic shock: relationship with adrenal insufficiency and clinical outcomes | Clin Lab. 2014;60(7):1105-14 | Population / region |
| Alam | Epidemiology, recognition and documentation of sepsis in the pre-hospital setting and associated clinical outcomes: a prospective multicenter study. | Acute Med. 2016;15(4):168-175 | No outcome of interest |
| Albino | Dialysis Complications in Acute Kidney Injury Patients Treated With Prolonged Intermittent Renal Replacement Therapy Sessions Lasting 10 Versus 6 Hours: Results of a Randomized Clinical Trial | Artificial Organs 2015, 39(5):423–431 | No outcome of interest |
| Albino | Dialysis complications in AKI patients treated with extended daily dialysis: is the duration of therapy important? | Biomed Res Int. 2014; 2014: 153626. | No outcome of interest |
| Alejandria | Intravenous immunoglobulin for treating sepsis, severe sepsis and septic shock. | Cochrane Database Syst Rev. 2013 Sep 16;(9):CD001090 | No outcome of interest |
| Aliberti | Phenotyping community-acquired pneumonia according to the presence of acute respiratory failure and severe sepsis | Respiratory Research 2014, 15:27 | No outcome of interest |
| Almeida | Differences in compliance with Surviving Sepsis Campaign recommendations according to hospital entrance time: day versus night | Critical Care 2013, 17:R79 | No outcome of interest |
| Amland | A Multidisciplinary Sepsis Program Enabled by a Two-Stage Clinical Decision Support System: Factors That Influence Patient Outcomes | Am J Med Qual. 2016 Nov;31(6):501-508. | No outcome of interest |
| Amrein | Vitamin D status and its association with season, hospital and sepsis mortality in critical illness | Critical Care 2014, 18:R47 | No outcome of interest |
| Anderson | Cholecystostomy offers no survival benefit in patients with acute acalculous cholecystitis and severe sepsis and shock | The Journal of surgical research 2014, 119(2) | No outcome of interest |
| Angelousi | Association between thyroid function tests at baseline and the outcome of patients with sepsis or septic shock: a systematic review | European Journal of Endocrinology (2011) 164 147–155 | No outcome of interest |
| April | Emergency department septic shock patient mortality with refractory hypotension vs hyperlactatemia: A retrospective cohort study. | Am J Emerg Med. 2017 Oct;35(10):1474-1479 | No outcome of interest |
| Arabi | Low-dose hydrocortisone in patients with cirrhosis and septic shock: a randomized controlled trial. | CMAJ:2010; 182(18):1971-1977 | Population / region |
| Arefian | Hospital-related cost of sepsis: A systematic review. | The Journal of infection 2017, 74(2):107-117 | No outcome of interest |
| Arulkumaran | Patients with end-stage renal disease admitted to the intensive care unit: systematic review. | British Journal of Anaesthesia 110 (1): 13–20 (2013) | No outcome of interest |
| Bahloul | Use of heptaminol hydrochloride for catecholamine weaning in septic shock. | American Journal of Therapeutics 2012, 19 | Population / region |
| Bahloul | Use of heptaminol hydrochloride for catecholamine weaning in septic shock. | American Journal of Therapeutics 2012, 19 | No original data or duplicate to other study |
| Balcan | Level of adrenomedullin in cases with adrenal defficiency and its relation to mortality in patients with sepsis. | Tuberk Toraks. 2016 Sep;64(3):191-197. | No access to full-text |
| Balcan | Level of adrenomedullin in cases with adrenal defficiency and its relation to mortality in patients with sepsis. | Tuberk Toraks. 2016 Sep;64(3):191-197. | No original data or duplicate to other study |
| Balik | Propafenone for supraventricular arrhythmias in septic shock-Comparison to amiodarone and metoprolol. | Journal of Critical Care 41 (2017) 16–23 | No original data or duplicate to other study |
| Bansal | Relative Survival Benefit and Morbidity with Fluids in Severe Sepsis - A Network Meta-Analysis of Alternative Therapies | Current Drug Safety, 2013, 8, 236-245 | No outcome of interest |
| Bansal | Relative Survival Benefit and Morbidity with Fluids in Severe Sepsis - A Network Meta-Analysis of Alternative Therapies | Current Drug Safety, 2013, 8, 236-245 | No original data or duplicate to other study |
| Barbar | Timing of Renal-Replacement Therapy in Patients with Acute Kidney Injury and Sepsis. | The New England journal of medicine 2018, 379 | No outcome of interest |
| Barbar | Timing of Renal-Replacement Therapy in Patients with Acute Kidney Injury and Sepsis. | The New England journal of medicine 2018, 379 | No original data or duplicate to other study |
| Barbouch | Urinary tract infections following renal transplantation: a single-center experience. | Saudi J Kidney Dis Transpl. 2012 Nov;23(6):1311-4. | Indication |
| Barchiesi | Epidemiology, clinical characteristics, and outcome of candidemia in a tertiary referral center in Italy from 2010 to 2014. | Infection. 2016 Apr;44(2):205-13. | Indication |
| Barie | Efficacy of Therapy with Recombinant Human Activated Protein C of Critically Ill Surgical Patients with Infection Complicated by Septic Shock and Multiple Organ Dysfunction Syndrome | SURGICAL INFECTIONS Volume 12, Number 6, 2011 | No outcome of interest |
| Barnay-Verdier | Emergence of autoantibodies to HMGB1 is associated with survival in patients with septic shock. | Intensive care medicine 2011; 37(6) | No outcome of interest |
| Barochia | Bundled care for septic shock: an analysis of clinical trials. | Crit Care Med. 2010 Feb; 38(2): 668–678 | No outcome of interest |
| Barochia | Bundled care for septic shock: An analysis of clinical trials | Crit Care Med 2010 Vol. 38, No. 2 | No outcome of interest |
| Battle | Long Term Health-Related Quality of Life in Survivors of Sepsis in South West Wales: An Epidemiological Study | PLoS One. 2014 Dec 30;9(12):e116304 | No outcome of interest |
| Bauman | Racial differences in vasopressor requirements for septic shock. | Shock. 2014 Mar;41(3):188-92 | No original data or duplicate to other study |
| Bauman | Racial differences in vasopressor requirements for septic shock. | SHOCK, Vol. 41, No. 3, pp. 188Y192, 2014 | No outcome of interest |
| Baumbach | Prevalence and Characteristics of Chronic Intensive Care-Related Pain: The Role of Severe Sepsis and Septic Shock. | Crit Care Med. 2016 Jun;44(6):1129-37 | No outcome of interest |
| Bayer | Effects of fluid resuscitation with synthetic colloids or crystalloids alone on shock reversal, fluid balance, and patient outcomes in patients with severe sepsis: a prospective sequential analysis. | Crit Care Med. 2012 Sep;40(9):2543-51 | No outcome of interest |
| Belletti | The effect of vasoactive drugs on mortality in patients with severe sepsis and septic shock. A network meta-analysis of randomized trials. | J Crit Care. 2017 Feb;37:91-98 | No original data or duplicate to other study |
| Belletti | The Effect of inotropes and vasopressors on mortality: a meta-analysis of randomized clinical trials. | Br J Anaesth. 2015 Nov;115(5):656-75 | No outcome of interest |
| Belletti | The effect of vasoactive drugs on mortality in patients with severe sepsis and septic shock. A network meta-analysis of randomized trials. | J Crit Care. 2017 Feb;37:91-98 | No outcome of interest |
| Belletti | Non-Adrenergic Vasopressors in Patients with or at Risk for Vasodilatory Shock. A Systematic Review and Meta-Analysis of Randomized Trials. | PLoS One. 2015 Nov 11;10(11):e0142605 | No outcome of interest |
| Bertacco | Risk Factors for Early Mortality in Liver Transplant Patients. | Transplant Proc. 2019 Jan - Feb;51(1):179-183 | No outcome of interest |
| Bessiere | Prognostic value of troponins in sepsis: a meta-analysis. | Intensive Care Med. 2013 Jul;39(7):1181-9. | No outcome of interest |
| Bewersdorf | The SPEED (sepsis patient evaluation in the emergency department) score: a risk stratification and outcome prediction tool. | Eur J Emerg Med. 2017 Jun;24(3):170-175 | Population / region |
| Bhadade | A Prospective Study of Acute Kidney Injury According to KDIGO Definition and its Mortality Predictors. | The Journal of the Association of Physicians of India | Population / region |
| Bhattacharjee | Levosimendan does not provide mortality benefit over dobutamine in adult patients with septic shock: A meta-analysis of randomized controlled trials. | J Clin Anesth. 2017 Jun;39:67-72 | No outcome of interest |
| Bindra | Is impaired cerebrovascular autoregulation associated with outcome in patients admitted to the ICU with early septic shock? | Crit Care Resusc. 2016 Jun;18(2):95-101. | No outcome of interest |
| Boerma | Effects of nitroglycerin on sublingual microcirculatory blood flow in patients with severe sepsis/septic shock after a strict resuscitation protocol: a double-blind randomized placebo controlled trial. | Crit Care Med. 2010 Jan;38(1):93-100 | No outcome of interest |
| Borken | Chronic Critical Illness from Sepsis Is Associated with an Enhanced TCR Response. | J Immunol. 2017 Jun 15;198(12):4781-4791 | No outcome of interest |
| Bouchard | A Prospective International Multicenter Study of AKI in the Intensive Care Unit. | Clin J Am Soc Nephrol, 10 (8), 1324-31 | Indication |
| Bouza | Characteristics, incidence and temporal trends of sepsis in elderly patients undergoing surgery. | BJS 2016; 103: e73–e82 | No outcome of interest |
| Boyd | Fluid resuscitation in septic shock: a positive fluid balance and elevated central venous pressure are associated with increased mortality. | Crit Care Med. 2011 Feb;39(2):259-65 | No outcome of interest |
| Brand | Intensity of Vasopressor Therapy for Septic Shock and the Risk of In-Hospital Death. | J Pain Symptom Manage. 2017 May;53(5):938-943 | No outcome of interest |
| Brooks | Sepsis caused by bloodstream infection in patients in the intensive care unit: the impact of inactive empiric antimicrobial therapy on outcome. | J Hosp Infect. 2018 Apr;98(4):369-374 | No outcome of interest |
| Browne | Analysis of factors predicting mortality of new patients commencing renal replacement therapy 10 years of follow-up. | BMC Nephrol. 2014 Jan 20;15:20 | Indication |
| Burrell | SEPSIS KILLS: early intervention saves lives. | Med J Aust. 2016 Feb 1;204(2):73. | No outcome of interest |
| Burston | A Role for Antimicrobial Stewardship in Clinical Sepsis Pathways: a Prospective Interventional Study. | Infect Control Hosp Epidemiol 2017;1–7 | Indication |
| Busani | Intravenous immunoglobulin in septic shock: review of the mechanisms of action and meta-analysis of the clinical effectiveness. | Minerva Anestesiol. 2016 May;82(5):559-72. Epub 2015 Oct 16. | No outcome of interest |
| Calfee | Subphenotypes in acute respiratory distress syndrome: latent class analysis of data from two randomised controlled trials. | Lancet Respir Med. 2014 Aug;2(8):611-20 | Indication |
| Cartin-Ceba | Epidemiology of critical care syndromes, organ failures, and life-support interventions in a suburban US community. | Chest. 2011 Dec;140(6):1447-1455 | Indication |
| Castellanos-Ortega | Impact of the Surviving Sepsis Campaign protocols on hospital length of stay and mortality in septic shock patients: results of a three-year follow-up quasi-experimental study. | Crit Care Med. 2010 Apr;38(4):1036-43 | No outcome of interest |
| Chamberlain | The severe sepsis bundles as processes of care: a meta-analysis. | Aust Crit Care. 2011 Nov;24(4):229-43 | No outcome of interest |
| Chancharoenthana | Enhanced vascular endothelial growth factor and inflammatory cytokine removal with online hemodiafiltration over high-flux hemodialysis in sepsis-related acute kidney injury patients. | Ther Apher Dial. 2013 Oct;17(5):557-63 | Population / region |
| Chang | Effects of Polymyxin B Hemoperfusion on Mortality in Patients With Severe Sepsis and Septic Shock: A Systemic Review, Meta-Analysis Update, and Disease Severity Subgroup Meta-Analysis. | Crit Care Med. 2017 Aug;45(8):e858-e864 | No outcome of interest |
| Chang | Effect of levosimendan on mortality in severe sepsis and septic shock: a meta-analysis of randomised trials. | BMJ Open. 2018 Mar 30;8(3):e019338 | No outcome of interest |
| Chao | Association of postdischarge rehabilitation with mortality in intensive care unit survivors of sepsis. | Am J Respir Crit Care Med. 2014 Nov 1;190(9):1003-11 | Population / region |
| Charles | Mr-Proadm Elevation Upon Icu Admission Predicts the Outcome of Septic Patients and is Correlated with Upcoming Fluid Overload. | Shock. 2017 Oct;48(4):418-426 | No outcome of interest |
| Chawla | Acute respiratory distress syndrome: Predictors of noninvasive ventilation failure and intensive care unit mortality in clinical practice. | J Crit Care. 2016 Feb;31(1):26-30 | Population / region |
| Chen | The effects of statin therapy on mortality in patients with sepsis: A meta-analysis of randomized trials. | Medicine (Baltimore). 2018 Aug;97(31):e11578 | No outcome of interest |
| Chen | Risk stratification and prognostic performance of the predisposition, infection, response, and organ dysfunction (PIRO) scoring system in septic patients in the emergency department: a cohort study. | Crit Care. 2014 Apr 16;18(2):R74 | Population / region |
| Cheng | Survival of septic adults compared with nonseptic adults receiving extracorporeal membrane oxygenation for cardiopulmonary failure: a propensity-matched analysis. | J Crit Care. 2013 Aug;28(4):532.e1-10. | Population / region |
| Cherfan | Etomidate and mortality in cirrhotic patients with septic shock. | BMC Clin Pharmacol. 2011 Dec 30;11:22. | Population / region |
| Cho | Clinical characteristics of sepsis-induced acute kidney injury in patients undergoing continuous renal replacement therapy. | Ren Fail. 2018 Nov;40(1):403-409 | Population / region |
| Chon | A comparison of the time from sepsis to inception of continuous renal replacement therapy versus RIFLE criteria in patients with septic acute kidney injury. | Shock. 2012 Jul;38(1):30-6 | Population / region |
| Choudhury | A randomized trial comparing terlipressin and noradrenaline in patients with cirrhosis and septic shock. | Liver Int. 2017 Apr;37(4):552-561 | Population / region |
| Chung | High-volume hemofiltration in adult burn patients with septic shock and acute kidney injury: a multicenter randomized controlled trial. | Crit Care. 2017 Nov 25;21(1):289 | No outcome of interest |
| Clancy | Detecting impaired myocardial relaxation in sepsis with a novel tissue Doppler parameter (septal e'/s'). | Crit Care. 2017 Jul 14;21(1):175 | No outcome of interest |
| Colomina-Climent | Mortality Reduction in Septic Shock by Plasma Adsorption (ROMPA): a protocol for a randomised clinical trial. | BMJ Open. 2016 Jul 12;6(7):e011856 | No original data or duplicate to other study |
| Costa | Erythrocyte selenium concentration predicts intensive care unit and hospital mortality in patients with septic shock: a prospective observational study. | Crit Care. 2014 May 7;18(3):R9 | No outcome of interest |
| D'Aragon | Blood pressure targets for vasopressor therapy: a systematic review. | Shock (Augusta, Ga.)2015, 43;6 530-539 | No outcome of interest |
| Damiani | Effect of performance improvement programs on compliance with sepsis bundles and mortality: a systematic review and meta-analysis of observational studies. | PLoSONE10(5):e0125827 | No outcome of interest |
| Daniels | The sepsis six and the severe sepsis resuscitation bundle: a prospective observational cohort study. | Emergency medicine journal : EMJ, 2011, 28, 6, 507-512 | No outcome of interest |
| Darwish | Challenges of Anticoagulation for Atrial Fibrillation in Patients With Severe Sepsis | Annals of Pharmacotherapy 47(10) 1266 –1271 | No outcome of interest |
| De Backer | Dopamine versus norepinephrine in the treatment of septic shock: A meta-analysis | Crit Care Med 2012 , 40,3: 725-730 | No outcome of interest |
| De Castilho | Heart rate variability as predictor of mortality in sepsis: A systematic review | PLoS One. 2018 Sep 11;13(9) | No outcome of interest |
| De Groot | The most commonly used disease severity scores are inappropriate for risk stratification of older emergency department sepsis patients: an observational multi-centre study. | Scand J Trauma Resusc Emerg Med. 2017 Sep 11;25(1):91 | No outcome of interest |
| De Groot | Inclusion of emergency department patients in early stages of sepsis in a quality improvement programme has the potential to improve survival: a prospective dual-centre study | Emerg Med J 2017;0:1–8. | No outcome of interest |
| De Miguel-Yanes | Trends in sepsis incidence and outcomes among people with or without type 2 diabetes mellitus in Spain (2008-2012). | Diabetes Res Clin Pract. 2015 Dec;110(3):266-75  Diabetes Res Clin Pract. 2015 Dec;110(3):266-75 | No outcome of interest |
| De Pascale | Clinical and microbiological outcome in septic patients with extremely low 25-hydroxyvitamin D levels at initiation of critical care. | Clin Microbiol Infect. 2016 May;22(5):456.e7-456.e13 | No outcome of interest |
| De Souza | Inverse association between serum creatinine and mortality in acute kidney injury. | J Bras Nefrol. 2014 Oct-Dec;36(4):469-75 | Population / region |
| Delaney | The role of albumin as a resuscitation fluid for patients with sepsis: a systematic review and meta-analysis. | Crit Care Med 2011 Vol. 39, No. 2 | No outcome of interest |
| DeMerle | Increased healthcare facility use in veterans surviving sepsis hospitalization. | Journal of Critical Care42(2017)59–64 | No outcome of interest |
| Deshpande | Statin therapy and mortality from sepsis: a meta-analysis of randomized trials. | Am J Med. 2015 Apr;128(4):410-7.e1 | No outcome of interest |
| Dhainaut | Extended drotrecogin alfa (activated) treatment in patients with prolonged septic shock | Intensive Care Med. 2009 Jul;35(7):1187-95 | No outcome of interest |
| Dhiman | Chronic Liver Failure-Sequential Organ Failure Assessment is better than the Asia-Pacific Association for the Study of Liver criteria for defining acute-on-chronic liver failure and predicting outcome. | World J Gastroenterol. Oct 28, 2014; 20(40): 14934-14941 | Population / region |
| Dinglas | One-year outcomes of rosuvastatin versus placebo in sepsis-associated acute respiratory distress syndrome: prospective follow-up of SAILS randomised trial. | Thorax 2016;71: 401–410. | No outcome of interest |
| Dmello | Outcomes of etomidate in severe sepsis and septic shock. | CHEST 2010; 138(6):1327–1332 | No outcome of interest |
| Donnino | Randomized, Double-Blind, Placebo-Controlled Trial of Thiamine as a Metabolic Resuscitator in Septic Shock: A Pilot Study. | Crit Care Med. 2016 Feb;44(2):360-7 | No outcome of interest |
| Dos Santos | Outcomes from a cohort of patients with acute kidney injury subjected to continuous venovenous hemodiafiltration: The role of negative fluid balance. | PLoS One. 2017 Apr 20;12(4):e0175897 | Population / region |
| Drewry | Antipyretic Therapy in Critically Ill Septic Patients: A Systematic Review and Meta-Analysis. | Crit Care Med. 2017 May; 45(5): 806–813 | No outcome of interest |
| Drey | C-terminal agrin fragment (CAF) reflects renal function in patients suffering from severe sepsis or septic shock. | Clin Lab. 2015;61(1-2):69-76. | No outcome of interest |
| Driessen | The influence of a change in septic shock definitions on intensive care epidemiology and outcome: comparison of sepsis-2 and sepsis-3 definitions. | Infect Dis (Lond). 2018 Mar;50(3):207-213 | No outcome of interest |
| Drumheller | Risk factors for mortality despite early protocolized resuscitation for severe sepsis and septic shock in the emergency department. | J Crit Care. 2016 Feb;31(1):13-20 | No outcome of interest |
| Dulhunty | Continuous infusion of beta-lactam antibiotics in severe sepsis: a multicenter double-blind, randomized controlled trial. | Clin Infect Dis. 2013 Jan;56(2):236-44. | No outcome of interest |
| Dulhunty | A Multicenter Randomized Trial of Continuous versus Intermittent beta-Lactam Infusion in Severe Sepsis. | Am J Respir Crit Care Med. 2015 Dec 1;192(11) | No outcome of interest |
| Einsiedel | Two nations: racial disparities in bloodstream infections recorded at Alice Springs Hospital, central Australia, 2001-2005. | Med J Aust. 2010 May 17;192(10):567-71 | Indication |
| Elfadawy | Transient versus persistent BK viremia and long-term outcomes after kidney and kidney-pancreas transplantation. | Clinical Journal of the American Society of Nephrology Vol. 9, Issue 3 | Indication |
| Eliakim-Raz | Duration of antibiotic treatment for acute pyelonephritis and septic urinary tract infection-- 7 days or less versus longer treatment: systematic review and meta-analysis of randomized controlled trials. | J Antimicrob Chemother 2013; 68: 2183–2191 | Indication |
| Elias | Derivation and validation of the acute organ failure score to predict outcome in critically ill patients: a cohort study. | Critical care medicine 2015, 43(4): 856-864 | Indication |
| Esper | The effect of diabetes mellitus on organ dysfunction with sepsis: an epidemiological study. | Critical Care (2009) 13:R18 | No outcome of interest |
| Failla | Systematic Review of Gender Differences in Sepsis Management and Outcomes. | Journal of Nursing Scholarship, 2017; 49:3, 1–13 | No outcome of interest |
| Fan | Predictive value of acute kidney injury in medical intensive care patients with sepsis originating from different infection sites. | Am J Med Sci. 2012 Aug;344(2):83-9 | Population / region |
| Fan | Efficacy and safety of low-molecular-weight heparin in patients with sepsis: a meta-analysis of randomized controlled trials. | Sci Rep. 2016 May 16;6:25984 | Population / region |
| Fang | Immune profiles and clinical outcomes between sepsis patients with or without active cancer requiring admission to intensive care units. | PLoS One. 2017 Jul 10;12(7):e0179749 | Population / region |
| Fawzy | Practice Patterns and Outcomes Associated With Choice of Initial Vasopressor Therapy for Septic Shock. | Crit Care Med. 2015 Oct;43(10):2141-6 | No outcome of interest |
| Fede | Renal failure and cirrhosis: a systematic review of mortality and prognosis. | Journal of Hepatology. 2012 vol. 56: 810–818 | Indication |
| Feissel | Pulse Wave Transit Time Measurements of Cardiac Output in Septic Shock Patients: A Comparison of the Estimated Continuous Cardiac Output System with Transthoracic Echocardiography. | PLoS One. 2015; 10(6): e0130489 | No outcome of interest |
| Femling | EMS patients and walk-in patients presenting with severe sepsis: differences in management and outcome. | Southern Medical Journal. 2014; 107(12):751-756 | No outcome of interest |
| Feng | Ulinastatin and/or thymosin alpha1 for severe sepsis: A systematic review and meta-analysis. | Trauma Acute Care Surg. 2016; 80(2) | Population / region |
| Fink-Neuboeck | Clinical impact of interleukin 6 as a predictive biomarker in the early diagnosis of postoperative systemic inflammatory response syndrome after major thoracic surgery: A prospective clinical trial. | Surgery. 2016 Aug;160(2):443-53 | Indication |
| Finlay | Identification of risk factors associated with acute kidney injury in patients admitted to acute medical units. | Clinical Medicine 2013, Vol 13, No 3: 233–8 | No outcome of interest |
| Fleischmann | Assessment of Global Incidence and Mortality of Hospital-treated Sepsis. Current Estimates and Limitations. | Am J Respir Crit Care Med. 2016 Feb 1;193(3):259-72 | No outcome of interest |
| Fujishima | Infection site is predictive of outcome in acute lung injury associated with severe sepsis and septic shock. | Respirology. 2016 Jul;21(5):898-904 | Population / region |
| Gabriel | Continuous peritoneal dialysis compared with daily hemodialysis in patients with acute kidney injury. | Perit Dial Int. 2009 Feb;29 Suppl 2:S62-71. | Population / region |
| Gaieski | The relationship between hospital volume and mortality in severe sepsis. | Am J Respir Crit Care Med Vol 190, Iss 6, pp 665–674, Sep 15, 2014 | No outcome of interest |
| Gameiro | Obesity, acute kidney injury and mortality in patients with sepsis: a cohort analysis. | Ren Fail. 2018 Nov;40(1):120-126. | No outcome of interest |
| Gamper | Vasopressors for hypotensive shock. | Cochrane Database Syst Rev. 2016 Feb 15;2:CD003709. | No outcome of interest |
| Gao | Association between IL-6-174G/C polymorphism and the risk of sepsis and mortality: a systematic review and meta-analysis. | PLoS One. 2015 Mar 3;10(3):e0118843. | No outcome of interest |
| Garnacho-Montero | Adequate antibiotic therapy prior to ICU admission in patients with severe sepsis and septic shock reduces hospital mortality. | Crit Care. 2015 Aug 27;19:302. | No outcome of interest |
| Gerth | Changes in health-related quality of life after discharge from an intensive care unit: a systematic review. | Anaesthesia. 2019 Jan;74(1):100-108. | No outcome of interest |
| Gibbison | Corticosteroids in septic shock: a systematic review and network meta-analysis. | Crit Care. 2017 Mar 28;21(1):78. | No outcome of interest |
| Gotmaker | Mortality is Greater in Septic Patients With Hyperlactatemia Than With Refractory Hypotension. | Shock. 2017 Sep;48(3):294-300. | No outcome of interest |
| Grimaldi | Profound and persistent decrease of circulating dendritic cells is associated with ICU-acquired infection in patients with septic shock. | Intensive Care Med. 2011 Sep;37(9):1438-46. | No outcome of interest |
| Grozdanovski | Early prognosis in patients with community-acquired severe sepsis and septic shock: analysis of 184 consecutive cases. | Prilozi. 2012;33(2):105-16. | No outcome of interest |
| Gu | The effect of goal-directed therapy on mortality in patients with sepsis - earlier is better: a meta-analysis of randomized controlled trials. | [Chest. 2015 Feb;147(2):335-346.](https://www.ncbi.nlm.nih.gov/pubmed/?term=10.1378%2Fchest.14-1012) | No outcome of interest |
| Gu | Single-dose etomidate does not increase mortality in patients with sepsis: a systematic review and meta-analysis of randomized controlled trials and observational studies. | Chest. 2015 Feb;147(2):335-346. | No outcome of interest |
| Gu | Relationship between Annualized Case Volume and Mortality in Sepsis: A Dose-Response Meta-analysis. | Anesthesiology. 2016 Jul;125(1):168-79. | No outcome of interest |
| Guirgis | Persistent organ dysfunction after severe sepsis: a systematic review. | J Crit Care. 2014 Jun;29(3):320-6. | No outcome of interest |
| Guo | Combination of Biomarkers in Predicting 28-Day Mortality for Septic Patients. | J Coll Physicians Surg Pak. 2018 Sep;28(9):672-676. | Population / region |
| Gutierrez-Pizarraya | Collaborative approach of individual participant data of prospective studies of de-escalation in non-immunosuppressed critically ill patients with sepsis. | Expert Rev Clin Pharmacol. 2017 Apr;10(4):457-465. | No outcome of interest |
| Hadem | Therapeutic plasma exchange as rescue therapy in severe sepsis and septic shock: retrospective observational single-centre study of 23 patients. | BMC Anesthesiol. 2014 Apr 7;14:24. | No outcome of interest |
| Hagiwara | Can recombinant human thrombomodulin increase survival among patients with severe septic-induced disseminated intravascular coagulation: a single-centre, open-label, randomised controlled trial. | BMJ Open. 2016 Dec 30;6(12):e012850. | Population / region |
| Hall | The impact of an omega-3 fatty acid rich lipid emulsion on fatty acid profiles in critically ill septic patients. | Prostaglandins Leukot Essent Fatty Acids. 2016 Sep;112:1-11. | No outcome of interest |
| Hamzic-Mehmedbasic | Renal Function Outcome Prognosis in Septic and Non-septic Acute Kidney Injury Patients. | Med Arch. 2015 Apr;69(2):77-80. | No outcome of interest |
| Han | Red blood cell distribution width predicts long-term outcomes in sepsis patients admitted to the intensive care unit. | Clin Chim Acta. 2018 Dec;487:112-116. | No outcome of interest |
| Haque | Pentoxifylline for treatment of sepsis and necrotizing enterocolitis in neonates. | Cochrane Database Syst Rev. 2011 Oct 5;(10):CD004205. | Indication |
| Haydar | Comparison of QSOFA score and SIRS criteria as screening mechanisms for emergency department sepsis. | Am J Emerg Med. 2017 Nov;35(11):1730-1733. | No outcome of interest |
| Hayer | Infection-related mortality is higher for kidney allograft recipients with pretransplant diabetes mellitus. | Diabetologia. 2014 Mar;57(3):554-61. | Indication |
| He | Umbilical cord-derived mesenchymal stem (stromal) cells for treatment of severe sepsis: aphase 1 clinical trial. | Transl Res. 2018 Sep;199:52-61. | Population / region |
| Henning | The Absence of Fever Is Associated With Higher Mortality and Decreased Antibiotic and IV Fluid Administration in Emergency Department Patients With Suspected Septic Shock. | Crit Care Med. 2017 Jun;45(6):e575-e582. | No outcome of interest |
| Hernandez | Effect of a Resuscitation Strategy Targeting Peripheral Perfusion Status vs Serum Lactate Levels on 28-Day Mortality Among Patients With Septic Shock: The ANDROMEDA-SHOCK Randomized Clinical Trial. | JAMA. 2019 Feb 19;321(7):654-664. | Population / region |
| Hofer | Central sympatholytics prolong survival in experimental sepsis. | Crit Care. 2009;13(1):R11. | Animal/Laboratory data only |
| Holcomb | Transfusion of plasma, platelets, and red blood cells in a 1:1:1 vs a 1:1:2 ratio and mortality in patients with severe trauma: the PROPPR randomized clinical trial. | JAMA. 2015 Feb 3;313(5):471-82. | Indication |
| Holst | Benefits and harms of red blood cell transfusions in patients with septic shock in the intensive care unit. | Dan Med J. 2016 Feb;63(2). pii: B5209. | No outcome of interest |
| Hooper | Randomized trial of automated, electronic monitoring to facilitate early detection of sepsis in the intensive care unit*. | Crit Care Med. 2012 Jul;40(7):2096-101. | No outcome of interest |
| Hou | Cardiac Output Monitoring Managing Intravenous Therapy (COMMIT) to Treat Emergency Department Patients with Sepsis. | Shock. 2016 Aug;46(2):132-8. | No outcome of interest |
| Hou | Endothelial Permeability and Hemostasis in Septic Shock: Results From the ProCESS Trial. | Chest. 2017 Jul;152(1):22-31. | No original data or duplicate to other study |
| Hsu | STROBE-compliant article: Blood Transfusions within the First 24 Hours of Hospitalization Did Not Impact Mortality Among Patients with Severe Sepsis. | Medicine (Baltimore). 2016 Jan;95(4):e2601. | Population / region |
| Hu | Effects of coupled plasma filtration adsorption on septic patients with multiple organ dysfunction syndrome. | Ren Fail. 2012;34(7):834-9. | Population / region |
| Huang | Is early ventricular dysfunction or dilatation associated with lower mortality rate in adult severe sepsis and septic shock? A meta-analysis. | Crit Care. 2013 May 27;17(3):R96. | No outcome of interest |
| Huang | Removal of humoral mediators and the effect on the survival of septic patients by hemoperfusion with neutral microporous resin column. | Ther Apher Dial. 2010 Dec;14(6):596-602. | Population / region |
| Huang | Effect on extrapulmonary sepsis-induced acute lung injury by hemoperfusion with neutral microporous resin column. | Ther Apher Dial. 2013 Aug;17(4):454-61. | Population / region |
| Huh | Low-dose hydrocortisone treatment for patients with septic shock: a pilot study comparing 3days with 7days. | Respirology. 2011 Oct;16(7):1088-95. | Population / region |
| Huh | Comparison of clinical outcomes between intermittent and continuous monitoring of central venous oxygen saturation (ScvO2) in patients with severe sepsis and septic shock: a pilot study. | Emerg Med J. 2013 Nov;30(11):906-9. | Population / region |
| Hung | Comparison of the Mortality in Emergency Department Sepsis Score, Modified Early Warning Score, Rapid Emergency Medicine Score and Rapid Acute Physiology Score for predicting the outcomes of adult splenic abscess patients in the emergency department. | PLoS One. 2017 Nov 1;12(11):e0187495. | Indication |
| Hunter | Comparing Quick Sequential Organ Failure Assessment Scores to End-tidal Carbon Dioxide as Mortality Predictors in Prehospital Patients with Suspected Sepsis. | West J Emerg Med. 2018 May;19(3):446-451. | No outcome of interest |
| Hwang | Low Accuracy of Positive qSOFA Criteria for Predicting 28-Day Mortality in Critically Ill Septic Patients During the Early Period After Emergency Department Presentation. | Ann Emerg Med. 2018 Jan;71(1):1-9.e2. | Population / region |
| Hyvernat | Effects of Increasing Hydrocortisone to 300 mg Per Day in the Treatment of Septic Shock: a Pilot Study. | Shock. 2016 Nov;46(5):498-505. | No outcome of interest |
| Iba | Efficacy and Bleeding Risk of Antithrombin Supplementation in Patients With Septic Disseminated Intravascular Coagulation: A Third Survey. | Clin Appl Thromb Hemost. 2017 Jul;23(5):422-428. | Population / region |
| Ibrahim | Can the characteristics of emergency department attendances predict poor hospital outcomes in patients with sepsis? | Singapore Med J. 2013 Nov;54(11):634-8. | No outcome of interest |
| Ide | Population pharmacokinetics/pharmacodynamics of linezolid in sepsis patients with and without continuous renal replacement therapy. | Int J Antimicrob Agents. 2018 May;51(5):745-751. | Population / region |
| Ishikura | Intravenous immunoglobulin improves sepsis-induced coagulopathy: A retrospective, single-center observational study. | J Crit Care. 2015 Jun;30(3):579-83. | Population / region |
| Izawa | Early-phase cumulative hypotension duration and severe-stage progression in oliguric acute kidney injury with and without sepsis: an observational study. | Crit Care. 2016 Dec 19;20(1):405. | Population / region |
| Jafarzadeh | Sepsis surveillance from administrative data in the absence of a perfect verification. | Ann Epidemiol. 2016 Oct;26(10):717-722.e1. | No outcome of interest |
| Jaimes | Unfractioned heparin for treatment of sepsis: A randomized clinical trial (The HETRASE Study). | Crit Care Med. 2009 Apr;37(4):1185-96. | Population / region |
| Jain | Procalcitonin as a prognostic marker for sepsis: a prospective observational study. | BMC Res Notes. 2014 Jul 17;7:458. | Population / region |
| Janda | The effect of statins on mortality from severe infections and sepsis: a systematic review and meta-analysis. | J Crit Care. 2010 Dec;25(4):656.e7-22. | No outcome of interest |
| Javed | Clinical predictors of early death from sepsis. | J Crit Care. 2017 Dec;42:30-34. | No outcome of interest |
| Jeong | Comparison of Accuracy of NUTRIC and Modified NUTRIC Scores in Predicting 28-Day Mortality in Patients with Sepsis: A Single Center Retrospective Study. | Nutrients. 2018 Jul 17;10(7). pii: E911. | Population / region |
| Jeong | Risk factors and outcomes of sepsis-induced myocardial dysfunction and stress-induced cardiomyopathy in sepsis or septic shock: A comparative retrospective study. | Medicine (Baltimore). 2018 Mar;97(13):e0263. | Population / region |
| Jiang | Albumin versus other fluids for fluid resuscitation in patients with sepsis: a meta-analysis. | PLoS One. 2014 Dec 4;9(12):e114666. | No outcome of interest |
| Jiang | Early goal-directed resuscitation for patients with severe sepsis and septic shock: a meta-analysis and trial sequential analysis. | Scand J Trauma Resusc Emerg Med. 2016 Mar 5;24:23. | No outcome of interest |
| Jie | Pentraxin 3, a Predicator for 28-Day Mortality in Patients With Septic Shock. | Am J Med Sci. 2017 Mar;353(3):242-246. | Population / region |
| Johansen | Profound endothelial damage predicts impending organ failure and death in sepsis. | Semin Thromb Hemost. 2015 Feb;41(1):16-25. | No outcome of interest |
| Johnston | Effect of Immediate Administration of Antibiotics in Patients With Sepsis in Tertiary Care: A Systematic Review and Meta-analysis. | Clin Ther. 2017 Jan;39(1):190-202.e6. | No outcome of interest |
| Jones | Lactate clearance vs central venous oxygen saturation as goals of early sepsis therapy: a randomized clinical trial. | JAMA. 2010 Feb 24;303(8):739-46. | No outcome of interest |
| Judd | Clinical and economic impact of a quality improvement initiative to enhance early recognition and treatment of sepsis. | Ann Pharmacother. 2014 Oct;48(10):1269-75. | No outcome of interest |
| Kaffarnik | Early diagnosis of sepsis-related hepatic dysfunction and its prognostic impact on survival: a prospective study with the LiMAx test. | Crit Care. 2013 Oct 31;17(5):R259. | No outcome of interest |
| Kalil | Early Goal-Directed Therapy for Sepsis: A Novel Solution for Discordant Survival Outcomes in Clinical Trials. | Crit Care Med. 2017 Apr;45(4):607-614. | Population / region |
| Kalil | Effectiveness and safety of drotrecogin alfa (activated) for severe sepsis: a meta-analysis and metaregression. | Lancet Infect Dis. 2012 Sep;12(9):678-86. | No outcome of interest |
| Karnad | Intravenous administration of ulinastatin (human urinary trypsin inhibitor) in severe sepsis: a multicenter randomized controlled study. | Intensive Care Med. 2014 Jun;40(6):830-8. | Population / region |
| Karvellas | Bacteremia, acute physiology and chronic health evaluation II and modified end stage liver disease are independent predictors of mortality in critically ill nontransplanted patients with acute on chronic liver failure. | Crit Care Med. 2010 Jan;38(1):121-6. | Indication |
| Katayama | Markers of acute kidney injury in patients with sepsis: the role of soluble thrombomodulin. | Crit Care. 2017 Aug 25;21(1):229. | Population / region |
| Kaukonen | Mortality related to severe sepsis and septic shock among critically ill patients in Australia and New Zealand, 2000-2012. | JAMA. 2014 Apr 2;311(13):1308-16. | No outcome of interest |
| Kawazoe | Effect of Dexmedetomidine on Mortality and Ventilator-Free Days in Patients Requiring Mechanical Ventilation With Sepsis: A Randomized Clinical Trial. | JAMA. 2017 Apr 4;317(13):1321-1328. | Population / region |
| Kawazoe | Mortality Effects of Prolonged Hemoperfusion Therapy Using a Polymyxin B-Immobilized Fiber Column for Patients with Septic Shock: A Sub-Analysis of the DESIRE Trial. | Blood Purif. 2018;46(4):309-314. | Population / region |
| Kellum | The Effects of Alternative Resuscitation Strategies on Acute Kidney Injury in Patients with Septic Shock. | Am J Respir Crit Care Med. 2016 Feb 1;193(3):281-7. | No outcome of interest |
| Kellum | Relationship Between Alternative Resuscitation Strategies, Host Response and Injury Biomarkers, and Outcome in Septic Shock: Analysis of the Protocol-Based Care for Early Septic Shock Study. | Crit Care Med. 2017 Mar;45(3):438-445. | No outcome of interest |
| Kelm | Fluid overload in patients with severe sepsis and septic shock treated with early goal-directed therapy is associated with increased acute need for fluid-related medical interventions and hospital death. | Shock. 2015 Jan;43(1):68-73. | No outcome of interest |
| Khanal | Comparison of outcomes by modality for critically ill patients requiring renal replacement therapy: a single-centre cohort study adjusting for time-varying illness severity and modality exposure. | Anaesth Intensive Care. 2012 Mar;40(2):260-8. | No outcome of interest |
| Khor | Randomized, double blind, placebo-controlled trial of fish-oil-based lipid emulsion infusion for treatment of critically ill patients with severe sepsis. | Asian J Surg. 2011 Jan;34(1):1-10. | Population / region |
| Khoury | The prognostic value of brain natriuretic peptide (BNP) in non-cardiac patients with sepsis, ultra-long follow-up. | J Crit Care. 2017 Dec;42:117-122. | No outcome of interest |
| Khwannimit | Validation of predisposition, infection, response and organ dysfunction score compared with standard severity scores in predicting hospital outcome in septic shock patients. | Minerva Anestesiol. 2013 Mar;79(3):257-63. Epub 2012 Dec 20. | No outcome of interest |
| Khwannimit | The direct costs of intensive care management and risk factors for financial burden of patients with severe sepsis and septic shock. | J Crit Care. 2015 Oct;30(5):929-34. | Population / region |
| Kikkawa | Significance of measuring S100A12 and sRAGE in the serum of sepsis patients with postoperative acute lung injury. | Dig Surg. 2010;27(4):307-12. | Population / region |
| Kim | An increase in red blood cell distribution width from baseline predicts mortality in patients with severe sepsis or septic shock. | Crit Care. 2013 Dec 9;17(6):R282. | Population / region |
| Kim | A Sepsis-related Diagnosis Impacts Interventions and Predicts Outcomes for Emergency Patients with Severe Sepsis. | West J Emerg Med. 2017 Oct;18(6):1098-1107. | No outcome of interest |
| Kim | Antibiotic Timing and Outcomes in Sepsis. | Am J Med Sci. 2018 Jun;355(6):524-529. | No outcome of interest |
| Kim | Is plasma neutrophil gelatinase-associated lipocalin a predictive biomarker for acute kidney injury in sepsis patients? A systematic review and meta-analysis. | J Crit Care. 2016 Jun;33:213-23. | No outcome of interest |
| Kim | A comparison of acute kidney injury classifications in patients with severe sepsis and septic shock. | Am J Med Sci. 2012 Nov;344(5):350-6. | Population / region |
| Kimura | The postoperative serum interleukin-15 concentration correlates with organ dysfunction and the prognosis of septic patients following emergency gastrointestinal surgery. | J Surg Res. 2012 Jun 15;175(2):e83-8. | Population / region |
| Ko | Prognosis of patients excluded by the definition of septic shock based on their lactate levels after initial fluid resuscitation: a prospective multi-center observational study. | Crit Care. 2018 Feb 24;22(1):47. | Population / region |
| Kondo | Early rehabilitation for the prevention of postintensive care syndrome in critically ill patients: a study protocol for a systematic review and meta-analysis. | BMJ Open. 2017 Mar 1;7(3):e013828. | No original data or duplicate to other study |
| Kong | Selenium supplementation for sepsis: a meta-analysis of randomized controlled trials. | Am J Emerg Med. 2013 Aug;31(8):1170-5. | No outcome of interest |
| Kongsayreepong | Predictors of Prolonged Length of Stay in General Surgical Intensive Care Unit. | J Med Assoc Thai. 2016 Sep;99 Suppl 6:S47-S54. | Population / region |
| Koupetori | Bloodstream infections and sepsis in Greece: over-time change of epidemiology and impact of de-escalation on final outcome. | BMC Infect Dis. 2014 May 18;14:272. | No outcome of interest |
| Kraft | Predictive Value of IL-8 for Sepsis and Severe Infections After Burn Injury: A Clinical Study. | Shock. 2015 Mar;43(3):222-7. | No outcome of interest |
| Kron | Extended daily on-line high-volume haemodiafiltration in septic multiple organ failure: a well-tolerated and feasible procedure. | Nephrol Dial Transplant. 2012 Jan;27(1):146-52. | No outcome of interest |
| Kron | Relative Blood Volume Monitoring during Renal Replacement Therapy in Critically Ill Patients with Septic Shock: A Preliminary Report. | Blood Purif. 2015;40(2):133-8. | No outcome of interest |
| Krychtiuk | Predictive value of low interleukin-33 in critically ill patients. | Cytokine. 2018 Mar;103:109-113. | No outcome of interest |
| Kuan | Emergency Department Management of Sepsis Patients: A Randomized, Goal-Oriented, Noninvasive Sepsis Trial. | Ann Emerg Med. 2016 Mar;67(3):367-378.e3. | Population / region |
| Kumar | A survival benefit of combination antibiotic therapy for serious infections associated with sepsis and septic shock is contingent only on the risk of death: a meta-analytic/meta-regression study. | Crit Care Med. 2010 Aug;38(8):1651-64. | No outcome of interest |
| Kushimoto | The impact of body temperature abnormalities on the disease severity and outcome in patients with severe sepsis: an analysis from a multicenter, prospective survey of severe sepsis. | Crit Care. 2013 Nov 13;17(6):R271. | Population / region |
| Lachmann | Innate immunity recovers earlier than acquired immunity during severe postoperative immunosuppression. | Int J Med Sci. 2018 Jan 1;15(1):1-9. | Indication |
| Lahmer | Influence of volume administration on Doppler-based renal resistive index, renal hemodynamics and renal function in medical intensive care unit patients with septic-induced acute kidney injury: a pilot study. | Int Urol Nephrol. 2016 Aug;48(8):1327-1334. | No outcome of interest |
| Lamontagne | Pooled analysis of higher versus lower blood pressure targets for vasopressor therapy septic and vasodilatory shock | Intensive Care Med. 2018 Jan;44(1):12-21. | Indication |
| Latour-Perez | Intensity of continuous renal replacement therapies in patients with severe sepsis and septic shock: a systematic review and meta-analysis. | Anaesth Intensive Care. 2011 May;39(3):373-83. | No outcome of interest |
| Laviolle | Gluco- and mineralocorticoid biological effects of a 7-day treatment with low doses of hydrocortisone and fludrocortisone in septic shock. | Intensive Care Med. 2012 Aug;38(8):1306-14. | No outcome of interest |
| Lavrentieva | Determinants of Outcome in Burn ICU Patients with Septic Shock. | J Burn Care Res. 2017 Jan/Feb;38(1):e172-e179. | No outcome of interest |
| Lee | Association of body temperature and antipyretic treatments with mortality of critically ill patients with and without sepsis: multi-centered prospective observational study. | Crit Care. 2012 Feb 28;16(1):R33. | Population / region |
| Lee | Preadmission Use of Calcium Channel Blocking Agents Is Associated With Improved Outcomes in Patients With Sepsis: A Population-Based Propensity Score-Matched Cohort Study. | Crit Care Med. 2017 Sep;45(9):1500-1508. | Population / region |
| Lee | Impact of antimicrobial strategies on clinical outcomes of adults with septic shock and community-onset Enterobacteriaceae bacteremia: de-escalation is beneficial. | Diagn Microbiol Infect Dis. 2015 Jun;82(2):158-64. | Population / region |
| Lee | Lactate Clearance and Vasopressor Seem to Be Predictors for Mortality in Severe Sepsis Patients with Lactic Acidosis Supplementing Sodium Bicarbonate: A Retrospective Analysis. | PLoS One. 2015 Dec 21;10(12):e0145181. | Population / region |
| Lee | Protocol-Based Resuscitation for Septic Shock: A Meta-Analysis of Randomized Trials and Observational Studies. | Yonsei Med J. 2016 Sep;57(5):1260-70. | No outcome of interest |
| Leedahl | Predictors of mortality among bacteremic patients with septic shock receiving appropriate antimicrobial therapy. | BMC Anesthesiol. 2014 Mar 25;14:21. | No outcome of interest |
| Leisman | Survival Benefit and Cost Savings From Compliance With a Simplified 3-Hour Sepsis Bundle in a Series of Prospective, Multisite, Observational Cohorts. | Crit Care Med. 2017 Mar;45(3):395-406. | No outcome of interest |
| Leisman | Association of Fluid Resuscitation Initiation Within 30 Minutes of Severe Sepsis and Septic Shock Recognition With Reduced Mortality and Length of Stay. | Ann Emerg Med. 2016 Sep;68(3):298-311. | No outcome of interest |
| Leligdowicz | Association between source of infection and hospital mortality in patients who have septic shock. | Am J Respir Crit Care Med. 2014 May 15;189(10):1204-13. | No outcome of interest |
| Levy | Outcomes of the Surviving Sepsis Campaign in intensive care units in the USA and Europe: a prospective cohort study. | Lancet Infect Dis. 2012 Dec;12(12):919-24. | No outcome of interest |
| Li | A systematic review of antibiotic dosing regimens for septic patients receiving continuous renal replacement therapy: do current studies supply sufficient data? | J Antimicrob Chemother. 2009 Nov;64(5):929-37. | No outcome of interest |
| Li | Prompt admission to intensive care is associated with improved survival in patients with severe sepsis and/or septic shock. | J Int Med Res. 2018 Oct;46(10):4071-4081. | No outcome of interest |
| Li | A meta-analysis of randomized controlled trials: Efficacy of selenium treatment for sepsis. | Medicine (Baltimore). 2019 Mar;98(9):e14733 | No outcome of interest |
| Lin | The outcomes and prognostic factors of the patients with unplanned intensive care unit readmissions. | Medicine (Baltimore). 2018 Jun;97(26):e11124. | Population / region |
| Liu | Effect of early goal directed therapy in the treatment of severe sepsis and/or septic shock. | Curr Med Res Opin. 2016 Nov;32(11):1773-1782 | No outcome of interest |
| Liu | Effect of ulinastatin combined with thymosin alpha1 on sepsis: A systematic review and meta-analysis of Chinese and Indian patients. | J Crit Care. 2017 Jun;39:259-266. | Population / region |
| Liu | Prognostic Value of Procalcitonin in Adult Patients with Sepsis: A Systematic Review and Meta-Analysis. | PLoS One. 2015 Jun 15;10(6):e0129450 | No outcome of interest |
| Liu | The efficacy of thymosin alpha1 as immunomodulatory treatment for sepsis: a systematic review of randomized controlled trials. | BMC Infect Dis. 2016 Sep 15;16:488 | Population / region |
| Liu | The influence of esmolol on septic shock and sepsis: A meta-analysis of randomized controlled studies. | Am J Emerg Med. 2018 Mar;36(3):470-474 | No outcome of interest |
| Liu | Prognostic impact of restored sinus rhythm in patients with sepsis and new-onset atrial fibrillation. | Crit Care. 2016 Nov 18;20(1):373. | Population / region |
| Livigni | Efficacy of coupled plasma filtration adsorption (CPFA) in patients with septic shock: a multicenter randomised controlled clinical trial. | BMJ Open. 2014 Jan 8;4(1):e003536 | No outcome of interest |
| Lock | The cost of graft failure and other severe complications after liver transplantation - experience from a German Transplant Center. | Ann Transplant. 2010 Jul-Sep;15(3):11-8. | No outcome of interest |
| Lopes | Long-term risk of mortality after acute kidney injury in patients with sepsis: a contemporary analysis. | BMC Nephrol. 2010 Jun 2;11:9. | No outcome of interest |
| Lorente | Red blood cell distribution width during the first week is associated with severity and mortality in septic patients. | PLoS One. 2014 Aug 25;9(8):e105436. | No original data or duplicate to other study |
| Lorente | Association between serum total antioxidant capacity and mortality in severe septic patients. | J Crit Care. 2015 Feb;30(1):217.e7-12 | No original data or duplicate to other study |
| Lorente | Association between serum substance P levels and mortality in patients with severe sepsis. | J Crit Care. 2015 Oct;30(5):924-8 | No original data or duplicate to other study |
| Lorente | Serum caspase 3 levels are associated with early mortality in severe septic patients. | J Crit Care. 2016 Aug;34:103-6. | No original data or duplicate to other study |
| Lorente | Association between Interleukin-6 Promoter Polymorphism (-174 G/C), Serum Interleukin-6 Levels and Mortality in Severe Septic Patients. | Int J Mol Sci. 2016 Nov 8;17(11) | No original data or duplicate to other study |
| Lorente | Non-survivor septic patients have persistently higher serum sCD40L levels than survivors. | J Crit Care. 2017 Oct;41:177-182. | No original data or duplicate to other study |
| Lorente | Sustained Low Serum Substance P Levels in Non-Surviving Septic Patients. | Int J Mol Sci. 2017 Jul; 18(7): 1531. | No original data or duplicate to other study |
| Lu | The effect of early goal-directed therapy on mortality in patients with severe sepsis and septic shock: a meta-analysis. | J Surg Res. 2016 May 15;202(2):389-97 | No outcome of interest |
| Lu | Early Goal-Directed Therapy in Severe Sepsis and Septic Shock: A Meta-Analysis and Trial Sequential Analysis of Randomized Controlled Trials. | J Intensive Care Med. 2018 May;33(5):296-309. | No outcome of interest |
| Luo | Increased cardiac index attenuates septic acute kidney injury: a prospective observational study. | BMC Anesthesiol. 2015 Mar 1;15:22 | Population / region |
| Lupei | Changes in vasopressin use and outcomes in surgical intensive care unit patients with septic shock. | Chirurgia (Bucur). 2009 Sep-Oct;104(5):575-81. | No outcome of interest |
| Lv | Early initiation of low-dose hydrocortisone treatment for septic shock in adults: A randomized clinical trial. | Am J Emerg Med. 2017 Dec;35(12):1810-1814 | Population / region |
| Lv | Comparative study of single/combination use of Huang-Lian-Jie-Du decoction and berberine on their protection on sepsis induced acute liver injury by NMR metabolic profiling. | J Pharm Biomed Anal. 2017 Oct 25;145:794-804. | Animal/Laboratory data only |
| Ma | Sources of Heterogeneity in Trials Reporting Hydroxyethyl Starch 130/0.4 or 0.42 Associated Excess Mortality in Septic Patients: A Systematic Review and Meta-regression. | Chin Med J. 2015 Sep 5; 128(17): 2374-2382 | No outcome of interest |
| Machado | Late recognition and illness severity are determinants of early death in severe septic patients. | Clinics (Sao Paulo). 2013 May; 68(5): 586-591 | No outcome of interest |
| Madsen | The DISPARITY Study: do gender differences exist in Surviving Sepsis Campaign resuscitation bundle completion, completion of individual bundle elements, or sepsis mortality? | Journal of Critical Care Volume 29, Issue 3, June 2014, Pages 473.e7-473.e11 | No outcome of interest |
| Mahmoodpoor | Which one is a better predictor of ICU mortality in septic patients? Comparison between serial serum lactate concentrations and its removal rate. | Journal of Critical Care Volume 44, April 2018, Pages 51-56 | No outcome of interest |
| Mahmoudi | Influence of sepsis on higher daily dose of amikacin pharmacokinetics in critically ill patients. | Eur. Rev. Med. Pharmacol. Sci. 2013 Feb; 17(3): 285-91 | No outcome of interest |
| Maitra | Accuracy of quick Sequential Organ Failure Assessment (qSOFA) score and systemic inflammatory response syndrome (SIRS) criteria for predicting mortality in hospitalized patients with suspected infection: a meta-analysis of observational studies. | Clinical Microbiology and Infection, Vol. 24, Issue 11, Pages 1123–1129 | No outcome of interest |
| Mallat | Central venous-to-arterial carbon dioxide partial pressure difference in early resuscitation from septic shock: a prospective observational study. | European Journal of Anaesthesiology. 31(7):371–380, JULY 2014 | No outcome of interest |
| Manaktala | Evaluating the impact of a computerized surveillance algorithm and decision support system on sepsis mortality. | J Am Med Inform Assoc. 2017 Jan;24(1):88-95. | No outcome of interest |
| Mann | Comparison of mortality associated with sepsis in the burn, trauma, and general intensive care unit patient: a systematic review of the literature. | Shock. 37(1):4–16, JANUARY 2012 | No outcome of interest |
| Mao | Effects of coupled plasma filtration adsorption on immune function of patients with multiple organ dysfunction syndrome. | Int J Artif Organs. 2009 Jan;32(1):31-38 | No outcome of interest |
| Mark | In-hospital mortality following treatment with red blood cell transfusion or inotropic therapy during early goal-directed therapy for septic shock: a retrospective propensity-adjusted analysis. | Crit. Care. 2014; 18(5): 496 | No outcome of interest |
| Marti-Carvajal | Human recombinant activated protein C for severe sepsis. | N Engl J Med. 2001 Mar 8;344(10):699-709. | No outcome of interest |
| Martin | NOREPINEPHRINE: NOT TOO MUCH, TOO LONG. | Shock: October 2015 - Volume 44 - Issue 4 - p 305-309 | No outcome of interest |
| Martinez | Impact of Source Control in Patients With Severe Sepsis and Septic Shock. | Crit Care Med. 2017 Jan;45(1):11-19. | No outcome of interest |
| Masson | Presepsin (soluble CD14 subtype) and procalcitonin levels for mortality prediction in sepsis: data from the Albumin Italian Outcome Sepsis trial. | Crit. Care 2014; 18(1) | No outcome of interest |
| Masson | Circulating presepsin (soluble CD14 subtype) as a marker of host response in patients with severe sepsis or septic shock: data from the multicenter, randomized ALBIOS trial. | Intensive Care Med 2015 41: 12-20 | No outcome of interest |
| Matera | Impact of interleukin-10, soluble CD25 and interferon-gamma on the prognosis and early diagnosis of bacteremic systemic inflammatory response syndrome: a prospective observational study. | Crit. Care. 2013; 17(2) | No outcome of interest |
| Mathias | A Review of GM-CSF Therapy in Sepsis. | Medicine. 2015 Dec; 94(50) | No outcome of interest |
| Mathias | Human Myeloid-derived Suppressor Cells are Associated With Chronic Immune Suppression After Severe Sepsis/Septic Shock. | Ann Surg. 2017 Apr; 265(4): 827-834 | No outcome of interest |
| Mauri | Persisting high levels of plasma pentraxin 3 over the first days after severe sepsis and septic shock onset are associated with mortality. | Intensive Care Medicine volume 36, pages621–629(2010) | No outcome of interest |
| Maybauer | Recombinant human activated protein C attenuates cardiovascular and microcirculatory dysfunction in acute lung injury and septic shock. | Crit. Care. 2010; 14(6) | No outcome of interest |
| McIntyre | The PRECISE RCT: evolution of an early septic shock fluid resuscitation trial. | Transfusion Medicine Reviews Volume 26, Issue 4, October 2012, Pages 333-341 | No outcome of interest |
| McKinley | Computer protocol facilitates evidence-based care of sepsis in the surgical intensive care unit. | The Journal of Trauma: Injury, Infection, and Critical Care: May 2011 - Volume 70 - Issue 5 - p 1153-1167 | No outcome of interest |
| Md Ralib | Plasma Neutrophil Gelatinase-Associated Lipocalin diagnosed acute kidney injury in patients with systemic inflammatory disease and sepsis | Nephrology (Carlton). 2017 May;22(5):412-419. | No outcome of interest |
| Medam | Risk factors for death in septic shock: A retrospective cohort study comparing trauma and non-trauma patients. | Medicine. 2017 Dec; 96(50) | No outcome of interest |
| Mehta | Sepsis as a cause and consequence of acute kidney injury: Program to Improve Care in Acute Renal Disease. | Intensive Care Med. 2011 Feb; 37(2): 241-248 | No outcome of interest |
| Melamed | The burden of sepsis-associated mortality in the United States from 1999 to 2005: an analysis of multiple-cause-of-death data. | Crit Care. 2009; 13(1) | No outcome of interest |
| Meng | Levosimendan Versus Dobutamine in Myocardial Injury Patients with Septic Shock: A Randomized Controlled Trial. | Med. Sci. Monit. 2016; 22: 1486-1496 | No outcome of interest |
| Meng | Effects of Early Continuous Venovenous Hemofiltration on E-Selectin, Hemodynamic Stability, and Ventilatory Function in Patients with Septic-Shock-Induced Acute Respiratory Distress Syndrome | Biomed Res Int. 2016;2016:7463130 | No outcome of interest |
| Meyer | Mortality Benefit of Recombinant Human Interleukin-1 Receptor Antagonist (rhIL1RA) for Sepsis Varies by Initial IL1RA Plasma Concentration | Crit. Care Med. 2018 Jan; 46(1): 21-28 | No outcome of interest |
| Meyer | A functional synonymous coding variant in the IL1RN gene is associated with survival in septic shock. | Am J. Respir. Crit. Care Med. 2014 Sep 15; 190(6): 656-664 | No outcome of interest |
| Mihajlovic | Endocan is useful biomarker of survival and severity in sepsis. | Microvasc Res. 2014 May;93:92-7. | No outcome of interest |
| Mikic | Clinical significance of soluble Fas plasma levels in patients with sepsis. | Vojnosanit Pregl. 2015 Jul;72(7):608-13 | No outcome of interest |
| Mikkelsen | Serum lactate is associated with mortality in severe sepsis independent of organ failure and shock. | Crit Care Med. 2009 May;37(5):1670-7 | No outcome of interest |
| Mikkelsen | The epidemiology of acute respiratory distress syndrome in patients presenting to the emergency department with severe sepsis. | The epidemiology of acute respiratory distress syndrome in patients presenting to the emergency department with severe sepsis. | No outcome of interest |
| Milano | Sepsis Bundle Adherence Is Associated with Improved Survival in Severe Sepsis or Septic Shock. | West J. Emerg. Med 2018 Sep; 19(5): 774-781 | No outcome of interest |
| Minneci | The effects of steroids during sepsis depend on dose and severity of illness: an updated meta-analysis. | Clin Microbiol Infect. 2009 Apr; 15(4):308-18 | No outcome of interest |
| Mitaka | A longer duration of polymyxin B-immobilized fiber column hemoperfusion improves pulmonary oxygenation in patients with septic shock. | Shock. 2009 Nov;32(5):478-83. | No outcome of interest |
| Mohr | Inter-hospital transfer is associated with increased mortality and costs in severe sepsis and septic shock: An instrumental variables approach | J Crit Care. 2016 Dec;36:187-194 | No outcome of interest |
| Mohr | Rural Patients With Severe Sepsis or Septic Shock Who Bypass Rural Hospitals Have Increased Mortality: An Instrumental Variables Approach. | Crit Care Med. 2017 Jan; 45(1): 85-93 | No outcome of interest |
| Moller | Scandinavian SSAI clinical practice guideline on choice of first-line vasopressor for patients with acute circulatory failure. | Acta Anaesthesiol Scand 2016 Nov; 60(10): 1347-1366 | No outcome of interest |
| Montini | Prognostic value of the reactive oxygen species in severe sepsis and septic shock patients: a pilot study. | Minerva Anestesiol. 2016 Dec;82(12):1306-1313 | No outcome of interest |
| Moraes | De-escalation, adequacy of antibiotic therapy and culture positivity in septic patients: an observational study. | Rev Bras Ter Intensiva. 2016 Jul-Sep; 28(3): 315-322 | No outcome of interest |
| Morales | Manati Medical Center Sepsis Management Epidemiological Study. | Bol Asoc Med P R. 2015 Apr-Jun;107(2):34-9 | No outcome of interest |
| Moran | Updating the evidence for the role of corticosteroids in severe sepsis and septic shock: a Bayesian meta-analytic perspective. | Critical Care 2010; 14(4): R134 | No outcome of interest |
| Morelli | Effect of heart rate control with esmolol on hemodynamic and clinical outcomes in patients with septic shock: a randomized clinical trial. | JAMA. 2013; 310(16):1683-1691 | No outcome of interest |
| Moreno | Time course of organ failure in patients with septic shock treated with hydrocortisone: results of the Corticus study. | Intensive Care Med. 2011 Nov;37(11) | No outcome of interest |
| Morris | Point-of-care lactate testing for sepsis at presentation to health care: a systematic review of patient outcomes | British Journal of General Practice. 2017 Dec; 67(665) | No outcome of interest |
| Moskowitz | Reasons for death in patients with sepsis and septic shock | J Crit Care. 2017 Apri; 38: 284-288 | No outcome of interest |
| Moskowitz | Thiamine as a Renal Protective Agent in Septic Shock. A Secondary Analysis of a Randomized, Double-Blind, Placebo-controlled Trial. | Annals of the American Thoracic Society. 2017 May; 14(5) 737-741 | No outcome of interest |
| Motzkus | ICU Admission Source as a Predictor of Mortality for Patients With Sepsis. | J Intensive Care Med. 2018 Sep;33(9):510-516. | No outcome of interest |
| Motzkus | Does Infection Site Matter? A Systematic Review of Infection Site Mortality in Sepsis. | Chest. October 2015 Volume 148, Issue 4, Supplement, Page 344A | No outcome of interest |
| Mouncey | The clinical and cost-effectiveness of early, goal-directed, protocolised resuscitation for emerging septic shock | The New England Journal of Medicine 2015; 372: 1301-1311 | No outcome of interest |
| Mousavi | New horizon in the treatment of sepsis: a systematic review of alternative medicine. | Journal of Complementary and Integrative Medicine, 2016 Dec 1; 13(4): 317-332 | No outcome of interest |
| Muady | Hemoglobin levels and blood transfusion in patients with sepsis in Internal Medicine Departments | BMC Infect Dis v.16, 2016 | No outcome of interest |
| Muller | Acute kidney injury with hydroxyethyl starch 130/0.42 in severe sepsis. | Acta Anaesthesiol Scand. 2015 Mar;59(3):329-36. | No outcome of interest |
| Muller | Effects of hydroxyethyl starch in subgroups of patients with severe sepsis: exploratory post-hoc analyses of a randomised trial. | Intensive Care Med. 2013 Nov;39(11):1963-71 | No outcome of interest |
| Müller | Utility of thromboelastography and/or thromboelastometry in adults with sepsis: a systematic review | Critical Care 2014, 18: R30 | No outcome of interest |
| Muronoi | Immature platelet fraction predicts coagulopathy-related platelet consumption and mortality in patients with sepsis. | Thromb Res. 2016 Aug;144:169-75. | No outcome of interest |
| Murugan | Plasma inflammatory and apoptosis markers are associated with dialysis dependence and death among critically ill patients receiving renal replacement therapy. | Nephrol Dial Transplant. 2014 Oct;29(10):1854-64 | No outcome of interest |
| Musikatavorn | Venous lactate in predicting the need for intensive care unit and mortality among nonelderly sepsis patients with stable hemodynamic. | Am J Emerg Med. 2015 Jul;33(7):925-30 | No outcome of interest |
| Nachtigall | Gender-related outcome difference is related to course of sepsis on mixed ICUs: a prospective, observational clinical study | Crit. Care. 2011; 15(3) | No outcome of interest |
| Nagata | Sepsis may not be a risk factor for mortality in patients with acute kidney injury treated with continuous renal replacement therapy. | Journal of Critical Care Volume 30, Issue 5, October 2015, Pages 998-1002 | No outcome of interest |
| Nakada | VPS13D Gene Variant Is Associated with Altered IL-6 Production and Mortality in Septic Shock. | Journal of Innate Immunity. 2015; 7:545-553 | No outcome of interest |
| Nakamura | Potential survival benefit of polymyxin B hemoperfusion in patients with septic shock: a propensity-matched cohort study. | Critical Care. 2017; 21:134 | No outcome of interest |
| Nardi | StO(2) guided early resuscitation in subjects with severe sepsis or septic shock: a pilot randomised trial. | Journal of Clinical Monitoring and Computing volume 27, pages215–221(2013 | No outcome of interest |
| Needham | Rosuvastatin versus placebo for delirium in intensive care and subsequent cognitive impairment in patients with sepsis-associated acute respiratory distress syndrome: an ancillary study to a randomised controlled trial. | Lacet Respir Med. 2016 March; 4(3): 203-212 | No outcome of interest |
| Nesseler | Long-term mortality and quality of life after septic shock: a follow-up observational study. | Intensive Care Med. 2013 May;39(5):881-8. | No outcome of interest |
| Neyra | Dipstick albuminuria and acute kidney injury recovery in critically ill septic patients. | Nephrology (Carlton). 2016 Jun;21(6):512-8 | No outcome of interest |
| Neyra | Cumulative Fluid Balance and Mortality in Septic Patients With or Without Acute Kidney Injury and Chronic Kidney Disease. | Crit. Care Med. 2016 Oct; 44(10): 1891-1900 | No outcome of interest |
| Neyra | Association of de novo dipstick albuminuria with severe acute kidney injury in critically ill septic patients. | Nephron Clin Pract. 2014;128(3-4):373-80 | No outcome of interest |
| Nguyen | Comparative Effectiveness of Second Vasoactive Agents in Septic Shock Refractory to Norepinephrine. | J Intensive Care Med. 2017 Aug;32(7):451-459 | No outcome of interest |
| Nguyen | Comparison of Predisposition, Insult/Infection, Response, and Organ dysfunction, Acute Physiology And Chronic Health Evaluation II, and Mortality in Emergency Department Sepsis in patients meeting criteria for early goal-directed therapy and the severe se | J Crit Care. 2012 Aug;27(4):362-9 | No outcome of interest |
| Nizamuddin | Interval Changes in Myocardial Performance Index Predict Outcome in Severe Sepsis. | J Cardiothorac Vasc Anesth. 2017 Jun;31(3):957-964 | No outcome of interest |
| Nouriel | Blood pressure variability as an indicator of sepsis severity in adult emergency department patients. | Am J Emerg Med. 2018 Apr;36(4):560-566. | No outcome of interest |
| Nygard | Aetiology, antimicrobial therapy and outcome of patients with community acquired severe sepsis: a prospective study in a Norwegian university hospital. | BMC Infec. Dis. 2014; 14:121 | No outcome of interest |
| Nygard | An observational study of community-acquired severe sepsis comparing intensive care and non-intensive care patients. | Acta Anaesthesiol Scand. 2017 Feb;61(2):194-204. | No outcome of interest |
| O'Dywer | The detection of microbial DNA but not cultured bacteria is associated with increased mortality in patients with suspected sepsis-a prospective multi-centre European observational study. | Clinical Microbiology and Infection. 2017. Vol. 23, Issue 3 | No outcome of interest |
| Oba | Mortality benefit of vasopressor and inotropic agents in septic shock: a Bayesian network meta-analysis of randomized controlled trials. | J Crit Care. 2014 Oct;29(5):706-10. doi: | No outcome of interest |
| Oeyen | Quality of life after intensive care: a systematic review of the literature. | Crit Care Med. 2010 Dec;38(12):2386-400. | No outcome of interest |
| Ogawa | Recombinant human soluble thrombomodulin improves mortality and respiratory dysfunction in patients with severe sepsis. | J Trauma Acute Care Surg. 2012 May;72(5):1150-7 | Population / region |
| Oliveira | Procalcitonin versus C-reactive protein for guiding antibiotic therapy in sepsis: a randomized trial. | Crit Care Med. 2013 Oct;41(10):2336-43 | Population / region |
| Ong | Short-Course Adjunctive Gentamicin as Empirical Therapy in Patients With Severe Sepsis and Septic Shock: A Prospective Observational Cohort Study | Clinical Infectious Diseases. 2017. Vol 64, Issue 12 | No outcome of interest |
| Ono | Removal of increased circulating CD4+CD25+Foxp3+ regulatory T cells in patients with septic shock using hemoperfusion with polymyxin B-immobilized fibers. | Surgery. 2013 Feb;153(2):262-71 | Population / region |
| Opal | Effect of eritoran, an antagonist of MD2-TLR4, on mortality in patients with severe sepsis: the ACCESS randomized trial. | JAMA. 2013 Mar 20;309(11):1154-62 | Population / region |
| Orwelius | Sepsis patients do not differ in health-related quality of life compared with other ICU patients. | Acta Anaesthesiol Scand. 2013 Oct;57(9):1201-5 | No outcome of interest |
| Osborn | Sepsis severity score: an internationally derived scoring system from the surviving sepsis campaign database*. | Crit Care Med. 2014 Sep;42(9):1969-76 | No outcome of interest |
| Ospina-Tascón | Combination of arterial lactate levels and venous-arterial CO2 to arterial-venous O 2 content difference ratio as markers of resuscitation in patients with septic shock. | Intensive Care Med. 2015 May; 41(5) | Population / region |
| Öz | Risk factors for multiorgan failure and mortality in severe sepsis patients who need intensive care unit follow-up | Tuberk Toraks. 2015 Sep;63(3): 147-57 | No outcome of interest |
| Palizas | Gastric tonometry versus cardiac index as resuscitation goals in septic shock: a multicenter, randomized, controlled trial. | Crit Care. 2009; 13(2): R44. | Population / region |
| Palomba | Comparative analysis of survival between elderly and non-elderly severe sepsis and septic shock resuscitated patients. | Einstein (Sao Paulo). 2015 Jul-Sep;13(3):357-63 | Population / region |
| Pan | Relative efficacy and safety of early lactate clearance-guided therapy resuscitation in patients with sepsis: A meta-analysis. | Medicine (Baltimore). 2019 Feb; 98(8): e14453. | No outcome of interest |
| Pappalardo | Protein C zymogen in severe sepsis: a double-blinded, placebo-controlled, randomized study. | Intensive Care Med. 2016 Nov;42(11):1706-1714 | No outcome of interest |
| Paratz | IMPOSE (IMProving Outcomes after Sepsis)-the effect of a multidisciplinary follow-up service on health-related quality of life in patients postsepsis syndromes-a double-blinded randomised controlled trial: protocol. | BMJ Open. 2014 May 26;4(5):e004966 | No original data or duplicate to other study |
| Park | Impact of serial measurements of lysophosphatidylcholine on 28-day mortality prediction in patients admitted to the intensive care unit with severe sepsis or septic shock. | J Crit Care. 2014 Oct;29(5):882.e5-11. | Population / region |
| Park | Quick sequential organ failure assessment compared to systemic inflammatory response syndrome for predicting sepsis in emergency department. | J Crit Care. 2017 Dec;42:12-17 | Population / region |
| Park | High-Dose Versus Conventional-Dose Continuous Venovenous Hemodiafiltration and Patient and Kidney Survival and Cytokine Removal in Sepsis-Associated Acute Kidney Injury: A Randomized Controlled Trial. | Am J Kidney Dis. 2016 Oct;68(4):599-608 | Population / region |
| Park | Mild hypoglycemia is independently associated with increased risk of mortality in patients with sepsis: a 3-year retrospective observational study. | Crit Care. 2012 Oct 12;16(5):R189 | Population / region |
| Park | The effect of early goal-directed therapy for treatment of severe sepsis or septic shock: A systemic review and meta-analysis. | J Crit Care. 2017 Apr;38:115-122. | No outcome of interest |
| Passos | Lactate clearance is associated with mortality in septic patients with acute kidney injury requiring continuous renal replacement therapy: A cohort study. | Medicine (Baltimore). 2016 Oct;95(40):e5112 | Population / region |
| Patel | Randomised trials of human albumin for adults with sepsis: systematic review and meta-analysis with trial sequential analysis of all-cause mortality. | BMJ. 2014 Jul 22;349:g4561 | No outcome of interest |
| Patel | Randomised trials of 6% tetrastarch (hydroxyethyl starch 130/0.4 or 0.42) for severe sepsis reporting mortality: systematic review and meta-analysis. | Intensive Care Med. 2013 May;39(5):811-22 | No outcome of interest |
| Patel | Assessing the effect of the Surviving Sepsis Campaign treatment guidelines on clinical outcomes in a community hospital. | Ann Pharmacother. 2010 Nov;44(11):1733-8. | No outcome of interest |
| Patel | Impact of duration of hypotension prior to norepinephrine initiation in medical intensive care unit patients with septic shock: A prospective observational study. | J Crit Care. 2017 Aug;40:178-183. | No outcome of interest |
| Paul | Systematic review and meta-analysis of the efficacy of appropriate empiric antibiotic therapy for sepsis. | Antimicrob Agents Chemother. 2010 Nov;54(11):4851-63 | No outcome of interest |
| Payen | Expression of monocyte human leukocyte antigen-DR in relation with sepsis severity and plasma mediators. | Minerva Anestesiol. 2009 Sep;75(9):484-93 | No outcome of interest |
| Penuelas | Prediction and Outcome of Intensive Care Unit-Acquired Paresis. | J Intensive Care Med. 2018 Jan;33(1):16-28. | Indication |
| Pepper | Increased body mass index and adjusted mortality in ICU patients with sepsis or septic shock: a systematic review and meta-analysis. | Crit Care. 2016 Jun 15;20(1):181 | No outcome of interest |
| Pereira | Risk factors for the progression of chronic kidney disease after acute kidney injury. | J Bras Nefrol. 2017 Jul-Sep;39(3):239-245 | Population / region |
| Perner | Comparing the effect of hydroxyethyl starch 130/0.4 with balanced crystalloid solution on mortality and kidney failure in patients with severe sepsis (6S--Scandinavian Starch for Severe Sepsis/Septic Shock trial): study protocol, design and rationale for | Trials. 2011 Jan 27;12:24. | No original data or duplicate to other study |
| Perner | Long-term outcomes in patients with severe sepsis randomised to resuscitation with hydroxyethyl starch 130/0.42 or Ringer's acetate. | Intensive Care Med. 2014 Jul;40(7):927-34 | No outcome of interest |
| Pestana | Compliance with a sepsis bundle and its effect on intensive care unit mortality in surgical septic shock patients. | J Trauma. 2010 Nov;69(5):1282-7 | No outcome of interest |
| Philippart | Decreased Risk of Ventilator-Associated Pneumonia in Sepsis Due to Intra-Abdominal Infection. | PLoS One. 2015 Sep 4;10(9):e0137262. | No outcome of interest |
| Phua | Characteristics and outcomes of culture-negative versus culture-positive severe sepsis. | Crit Care. 2013 Sep 12;17(5):R202 | Population / region |
| Pickkers | Alkaline phosphatase for treatment of sepsis-induced acute kidney injury: a prospective randomized double-blind placebo-controlled trial. | Crit Care. 2012 Jan 23;16(1):R14 | No outcome of interest |
| Pliszczynski | Better actual 10-year renal transplant outcomes of 80% reduced cyclosporine exposure with sirolimus base therapy compared with full cyclosporine exposure without or with concomittant sirolimus treatment. | Transplant Proc. 2011 Dec;43(10):3657-68. | Indication |
| Plurad | The association of race and survival from sepsis after injury. | Am Surg. 2010 Jan;76(1):43-7 | No outcome of interest |
| Poddar | Reduction in procalcitonin level and outcome in critically ill children with severe sepsis/septic shock-A pilot study. | J Crit Care. 2016 Dec;36:230-233 | Population / region |
| Poukkanen | Hemodynamic variables and progression of acute kidney injury in critically ill patients with severe sepsis: data from the prospective observational FINNAKI study. | Crit Care. 2013 Dec 13;17(6):R295. | No outcome of interest |
| Povoa | Clinical impact of stress dose steroids in patients with septic shock: insights from the PROWESS-Shock trial. | Crit Care. 2015 Apr 28;19:193 | No outcome of interest |
| Prakash | Changes in fibrinolysis and severity of organ failure in sepsis: a prospective observational study using point-of-care test--ROTEM. | J Crit Care. 2015 Apr;30(2):264-70 | No outcome of interest |
| Premuzic | Differences in CVVH vs. CVVHDF in the management of sepsis-induced acute kidney injury in critically ill patients. | J Artif Organs. 2017 Dec;20(4):326-334 | No outcome of interest |
| Prescott | Obesity and 1-year outcomes in older Americans with severe sepsis. | Crit Care Med. 2014 Aug;42(8):1766-74 | No outcome of interest |
| Prkno | Procalcitonin-guided therapy in intensive care unit patients with severe sepsis and septic shock--a systematic review and meta-analysis. | Crit Care. 2013 Dec 11;17(6):R291. | No outcome of interest |
| Puskarich | Lactate Clearance in Septic Shock Is Not a Surrogate for Improved Microcirculatory Flow. | Acad Emerg Med. 2016 Jun;23(6):690-3 | No outcome of interest |
| Puskarich | Prognostic value and agreement of achieving lactate clearance or central venous oxygen saturation goals during early sepsis resuscitation. | Acad Emerg Med. 2012 Mar;19(3):252-8. | No outcome of interest |
| Puskarich | Association between timing of antibiotic administration and mortality from septic shock in patients treated with a quantitative resuscitation protocol. | Crit Care Med. 2011 Sep;39(9):2066-71 | No outcome of interest |
| Puskarich | Outcomes of patients undergoing early sepsis resuscitation for cryptic shock compared with overt shock. | Resuscitation. 2011 Oct;82(10):1289-93 | No outcome of interest |
| Qi | Incidence and risk of severe infections associated with anti-epidermal growth factor receptor monoclonal antibodies in cancer patients: a systematic review and meta-analysis. | BMC Med. 2014 Nov 5;12:203 | No outcome of interest |
| Qiu | Antitumor necrosis factor therapy is associated with improved survival in clinical sepsis trials: a meta-analysis. | Crit Care Med. 2013 Oct;41(10):2419-29 | No outcome of interest |
| Rathour | PIRO concept: staging of sepsis. | J Postgrad Med. 2015 Oct-Dec;61(4):235-42 | Population / region |
| Rattan, R | Patients with Complicated Intra-Abdominal Infection Presenting with Sepsis Do Not Require Longer Duration of Antimicrobial Therapy. | J Am Coll Surg. 2016 Jul;223(1):206-7 | No outcome of interest |
| Ratzinger, Franz | Sepsis in standard care: patients' characteristics, effectiveness of antimicrobial therapy and patient outcome--a cohort study. | Infection. 2015 Jun;43(3):345-52 | No outcome of interest |
| Reed, Harrell Lester | Dependence of All-Cause Standardized In-Hospital Mortality on Sepsis Mortality Between 2005 and 2010. | Am J Med Qual. 2014 Jul-Aug;29(4):315-22 | No outcome of interest |
| Rhee, Chanu | Incidence and Trends of Sepsis in US Hospitals Using Clinical vs Claims Data, 2009-2014. | JAMA. 2017 Oct 3;318(13):1241-1249 | No outcome of interest |
| Rhee, Chanu | Objective Sepsis Surveillance Using Electronic Clinical Data. | Infect Control Hosp Epidemiol. 2016 Feb;37(2):163-7 | No outcome of interest |
| Rhee, Harin | Short- and Long-Term Mortality Rates of Elderly Acute Kidney Injury Patients Who Underwent Continuous Renal Replacement Therapy. | PLoS One. 2016 Nov 22;11(11):e0167067 | No outcome of interest |
| Rhodes, Andrew | The Surviving Sepsis Campaign bundles and outcome: results from the International Multicentre Prevalence Study on Sepsis (the IMPreSS study). | Intensive Care Med. 2015 Sep;41(9):1620-8 | No outcome of interest |
| Ribas Ripoli, Vicent | Metabolite analysis in sepsis through conditional independence maps. | Conf Proc IEEE Eng Med Biol Soc. 2015;2015:6477-80 | Animal/laboratory data only |
| Rice, T | Enteral omega-3 fatty acid, gamma-linolenic acid, and antioxidant supplementation in acute lung injury. | JAMA. 2011 Oct 12;306(14):1574-81 | No outcome of interest |
| Richard, J | Preload dependence indices to titrate volume expansion during septic shock: a randomized controlled trial. | Crit Care. 2015 Jan 8;19:5 | No outcome of interest |
| Richards, G | CURB-65, PSI, and APACHE II to assess mortality risk in patients with severe sepsis and community acquired pneumonia in PROWESS. | J Intensive Care Med. 2011 Jan-Feb;26(1):34-40 | No outcome of interest |
| Riche, F | Reversal of neutrophil-to-lymphocyte count ratio in early versus late death from septic shock. | Crit Care. 2015 Dec 16;19:439 | No outcome of interest |
| Rimachi, R | Lactate/pyruvate ratio as a marker of tissue hypoxia in circulatory and septic shock. | Anaesth Intensive Care. 2012 May;40(3):427-32 | No outcome of interest |
| Roberts | SaMpling Antibiotics in Renal Replacement Therapy (SMARRT): an observational pharmacokinetic study in critically ill patients. | BMC Infect Dis. 2016 Mar 1;16:103 | Indication |
| Rochwerg | Assessment of Postresuscitation Volume Status by Bioimpedance Analysis in Patients with Sepsis in the Intensive Care Unit: A Pilot Observational Study. | Can Respir J. 2016;2016:8671742 | No outcome of interest |
| Rodriguez | Mortality and regional oxygen saturation index in septic shock patients: a pilot study. | J Trauma. 2011 May;70(5):1145-52 | No outcome of interest |
| Roveran Genga | Two-year follow-up of patients with septic shock presenting with low HDL: the effect upon acute kidney injury, death and estimated glomerular filtration rate. | J Intern Med. 2017 May;281(5):518-529 | No outcome of interest |
| Rowan | Early, Goal-Directed Therapy for Septic Shock - A Patient-Level Meta-Analysis. | N Engl J Med. 2017 Jun 8;376(23):2223-2234 | No original data or duplicate to other study |
| Ruiz-Mesa | Factors associated with severe sepsis or septic shock in complicated pyelonephritis. | Medicine (Baltimore). 2017 Oct;96(43):e8371 | Indication |
| Rumbus | Fever Is Associated with Reduced, Hypothermia with Increased Mortality in Septic Patients: A Meta-Analysis of Clinical Trials. | PLoS One. 2017 Jan 12;12(1):e0170152 | No outcome of interest |
| Russell | The Septic Shock 3.0 Definition and Trials: A Vasopressin and Septic Shock Trial Experience. | Crit Care Med. 2017 Jun;45(6):940-948 | No outcome of interest |
| Russell | Interaction of vasopressin infusion, corticosteroid treatment, and mortality of septic shock. | Crit Care Med. 2009 Mar;37(3):811-8 | No outcome of interest |
| Rygard | Long-term outcomes in patients with septic shock transfused at a lower versus a higher haemoglobin threshold: the TRISS randomised, multicentre clinical trial. | Intensive Care Med. 2016 Nov;42(11) | No outcome of interest |
| Rygard | Low-dose corticosteroids for adult patients with septic shock: a systematic review with meta-analysis and trial sequential analysis. | Intensive Care Med. 2018 Jul;44(7):1003-1016 | No original data or duplicate to other study |
| Saito | Efficacy of polymyxin B-immobilized fiber hemoperfusion for patients with septic shock caused by Gram-negative bacillus infection. | PLoS One. 2017 Mar 30;12(3):e0173633 | Population / region |
| Sakr | Higher Fluid Balance Increases the Risk of Death From Sepsis: Results From a Large International Audit. | Crit Care Med. 2017 Mar;45(3):386-394 | No outcome of interest |
| Sakr | Epidemiology and outcome of sepsis syndromes in Italian ICUs: a muticentre, observational cohort study in the region of Piedmont. | Minerva Anestesiol. 2013 Sep;79(9):993-1002 | No outcome of interest |
| Savioli | Tight glycemic control may favor fibrinolysis in patients with sepsis. | Crit Care Med. 2009 Feb;37(2):424-31 | No outcome of interest |
| Sawa | Direct hemoperfusion with a polymyxin B column versus vasopressin for gram negative septic shock: a matched cohort study of the effect on survival | Clin Nephrol. 2013 Jun;79(6):463-70 | No outcome of interest |
| Scheetz | Life-years gained with meropenem over ciprofloxacin in penicillin-allergic patients with gram-negative bacilli sepsis: results of a probabilistic model. | Pharmacotherapy. 2011 May;31(5):469-79. | No outcome of interest |
| Scherag | A patient cohort on long-term sequelae of sepsis survivors: study protocol of the Mid-German Sepsis Cohort. | BMJ Open. 2017 Aug 23;7(8):e016827 | No original data or duplicate to other study |
| Schmidt | Effect of a Primary Care Management Intervention on Mental Health-Related Quality of Life Among Survivors of Sepsis: A Randomized Clinical Trial. | JAMA. 2016 Jun 28;315(24):2703-11 | No outcome of interest |
| Schortgen | Fever control using external cooling in septic shock: a randomized controlled trial. | Am J Respir Crit Care Med. 2012 May 15;185(10):1088-95 | No outcome of interest |
| Schortgen | Respective impact of lowering body temperature and heart rate on mortality in septic shock: mediation analysis of a randomized trial. | Intensive Care Med. 2015 Oct;41(10):1800-8 | No outcome of interest |
| Schramm | Septic shock: a multidisciplinary response team and weekly feedback to clinicians improve the process of care and mortality. | Crit Care Med. 2011 Feb;39(2):252-8. | No outcome of interest |
| Sehgal | A randomized trial of Mycobacterium w in severe sepsis. | J Crit Care. 2015 Feb;30(1):85-9 | Population / region |
| Sekino | Intestinal fatty acid-binding protein level as a predictor of 28-day mortality and bowel ischemia in patients with septic shock: A preliminary study. | J Crit Care. 2017 Dec;42:92-100 | Population / region |
| Semler | An Electronic Tool for the Evaluation and Treatment of Sepsis in the ICU: A Randomized Controlled Trial. | Crit Care Med. 2015 Aug;43(8):1595-602 | No outcome of interest |
| Seo | Hypoalbuminemia, Low Base Excess Values, and Tachypnea Predict 28-Day Mortality in Severe Sepsis and Septic Shock Patients in the Emergency Department. | Yonsei Med J. 2016 Nov;57(6):1361-9. | Population / region |
| Serpa | The use of the pulse oximetric saturation/fraction of inspired oxygen ratio for risk stratification of patients with severe sepsis and septic shock. | J Crit Care. 2013 Oct;28(5):681-6 | Population / region |
| Seymour | Metabolomics in pneumonia and sepsis: an analysis of the GenIMS cohort study. | Intensive Care Med. 2013 Aug;39(8):1423-34 | Indication |
| Shankar-Hari | Developing a New Definition and Assessing New Clinical Criteria for Septic Shock: For the Third International Consensus Definitions for Sepsis and Septic Shock (Sepsis-3). | JAMA. 2016 Feb 23;315(8):775-87 | No outcome of interest |
| Shankar-Hari | Evidence for a causal link between sepsis and long-term mortality: a systematic review of epidemiologic studies. | Crit Care. 2016 Apr 13;20:101 | No outcome of interest |
| Shapiro | The association of endothelial cell signaling, severity of illness, and organ dysfunction in sepsis. | Crit Care. 2010;14(5):R182 | No outcome of interest |
| Shapiro | The association of near-infrared spectroscopy-derived tissue oxygenation measurements with sepsis syndromes, organ dysfunction and mortality in emergency department patients with sepsis. | Crit Care. 2011;15(5):R223 | No outcome of interest |
| Shaw | Severe protein C deficiency is associated with organ dysfunction in patients with severe sepsis. | J Crit Care. 2011 Dec;26(6):539-45. | No outcome of interest |
| Shehabi | Procalcitonin algorithm in critically ill adults with undifferentiated infection or suspected sepsis. A randomized controlled trial. | Am J Respir Crit Care Med. 2014 Nov 15;190(10):1102-10 | Indication |
| Sherwin | Do low-dose corticosteroids improve mortality or shock reversal in patients with septic shock? A systematic review and position statement prepared for the American Academy of Emergency Medicine. | J Emerg Med. 2012 Jul;43(1):7-12 | No outcome of interest |
| Sheyin | The prognostic significance of troponin elevation in patients with sepsis: a meta-analysis. | Heart Lung. 2015 Jan-Feb;44(1):75-81 | No outcome of interest |
| Shi | Xuebijing in the treatment of patients with sepsis. | Am J Emerg Med. 2017 Feb;35(2):285-291 | No outcome of interest |
| Shiga | Continuous hemodiafiltration with a cytokine-adsorbing hemofilter in patients with septic shock: a preliminary report. | Blood Purif. 2014;38(3-4):211-8 | Population / region |
| Shimizu | The clinical significance of serum procalcitonin levels following direct hemoperfusion with polymyxin B-immobilized fiber column in septic patients with colorectal perforation. | Eur Surg Res. 2009;42(2):109-17 | Population / region |
| Shukeri | Sepsis mortality score for the prediction of mortality in septic patients. | J Crit Care. 2018 Feb;43:163-168 | Population / region |
| Silva | De-escalation of antimicrobial treatment for adults with sepsis, severe sepsis or septic shock. | Cochrane Database Syst Rev. 2013 Mar 28;(3):CD007934 | No outcome of interest |
| Silversides | Conservative fluid management or deresuscitation for patients with sepsis or acute respiratory distress syndrome following the resuscitation phase of critical illness: a systematic review and meta-analysis | Intensive Care Med. 2017 Feb;43(2):155-170 | No outcome of interest |
| Simpson | Early goal-directed therapy for severe sepsis and septic shock: A living systematic review. | J Crit Care. 2016 Dec;36:43-48 | No outcome of interest |
| Sjovall | Empirical mono- versus combination antibiotic therapy in adult intensive care patients with severe sepsis - A systematic review with meta-analysis and trial sequential analysis. | J Infect. 2017 Apr;74(4):331-344 | No outcome of interest |
| Smilowitz | Comparison of Outcomes of Patients With Sepsis With Versus Without Acute Myocardial Infarction and Comparison of Invasive Versus Noninvasive Management of the Patients With Infarction | Am J Cardiol. 2016 Apr 1;117(7):1065-71. | No outcome of interest |
| Smith | Adjunctive treatment of abdominal catastrophes and sepsis with direct peritoneal resuscitation: indications for use in acute care surgery. | J Trauma Acute Care Surg. 2014 Sep;77(3):393-8 | Indication |
| Smith | Not All Abdomens Are the Same: A Comparison of Damage Control Surgery for Intra-abdominal Sepsis versus Trauma. | Am Surg. 2016 May;82(5):427-32. | No outcome of interest |
| Soares | An evaluation of the feasibility, cost and value of information of a multicentre randomised controlled trial of intravenous immunoglobulin for sepsis (severe sepsis and septic shock): incorporating a systematic review, meta-analysis and value of informat | Health Technol Assess. 2012;16(7):1-186 | No outcome of interest |
| Sohn | Outcome of delayed resuscitation bundle achievement in emergency department patients with septic shock. | Intern Emerg Med. 2014 Sep;9(6):671-6 | Population / region |
| Song | Intensive insulin therapy for septic patients: a meta-analysis of randomized controlled trials. | Biomed Res Int. 2014;2014:698265 | No outcome of interest |
| Sood | Early reversible acute kidney injury is associated with improved survival in septic shock. | J Crit Care. 2014 Oct;29(5):711-7 | No outcome of interest |
| Steingrub | Treatment with neuromuscular blocking agents and the risk of in-hospital mortality among mechanically ventilated patients with severe sepsis. | Crit Care Med. 2014 Jan;42(1):90-6. | No outcome of interest |
| Sterling | The Impact of Timing of Antibiotics on Outcomes in Severe Sepsis and Septic Shock: A Systematic Review and Meta-Analysis. | Crit Care Med. 2015 Sep;43(9):1907-15. | No outcome of interest |
| Stiermaier | Incidence and long-term outcome of sepsis on general wards and in an ICU at the General Hospital of Vienna: an observational cohort study. | Wien Klin Wochenschr. 2013 Jun;125(11-12):302-8. | No outcome of interest |
| Stortz | Benchmarking clinical outcomes and the immunocatabolic phenotype of chronic critical illness after sepsis in surgical intensive care unit patients. | J Trauma Acute Care Surg. 2018 Feb;84(2):342-349 | No outcome of interest |
| Stoumpos | Continued monitoring of acute kidney injury survivors might not be necessary in those regaining an estimated glomerular filtration rate >60 mL/min at 1 year. | Nephrol Dial Transplant. 2017 Jan 1;32(1):81-88 | No outcome of interest |
| Su | Dynamic changes in amino acid concentration profiles in patients with sepsis. | PLoS One. 2015; 10(4): e0121933 | Population / region |
| Su | Value of soluble TREM-1, procalcitonin, and C-reactive protein serum levels as biomarkers for detecting bacteremia among sepsis patients with new fever in intensive care units: a prospective cohort study. | BMC Infect Dis. 2012; 12: 157. | Population / region |
| Su | Diagnostic value of urine sCD163 levels for sepsis and relevant acute kidney injury: a prospective study. | BMC Nephrol. 2012; 13: 123. | Population / region |
| Su | Identification of novel biomarkers for sepsis prognosis via urinary proteomic analysis using iTRAQ labeling and 2D-LC-MS/MS. | PLoS One. 2013; 8(1): e54237 | Population / region |
| Suarez | Cost-effectiveness of the Surviving Sepsis Campaign protocol for severe sepsis: a prospective nation-wide study in Spain. | Intensive Care Med (2011) 37:444–452 | No outcome of interest |
| Suberviola | Prognostic value of proadrenomedullin in severe sepsis and septic shock patients with community-acquired pneumonia. | Swiss Med Wkly. 2012;142:w13542 | No outcome of interest |
| Suberviola | Association between exposure to angiotensin-converting enzyme inhibitors and angiotensin receptor blockers prior to septic shock and acute kidney injury. | Med Intensiva. 2017 Jan - Feb;41(1):21-27 | No outcome of interest |
| Suberviola | Effects of antibiotic administration delay and inadequacy upon the survival of septic shock patients. | Med Intensiva. 2015 Nov;39(8):459-66 | No outcome of interest |
| Suh | Acute kidney injury in patients with sepsis and septic shock: risk factors and clinical outcomes. | Yonsei Med J. 2013 Jul 1; 54(4): 965–972 | Population / region |
| Sun | Continuous venovenous hemofiltration versus extended daily hemofiltration in patients with septic acute kidney injury: a retrospective cohort study. | Crit Care. 2014 Apr 9;18(2):R70. | Population / region |
| Sun | Total ginsenosides synergize with ulinastatin against septic acute lung injury and acute respiratory distress syndrome. | Int J Clin Exp Pathol. 2015 Jun 1;8(6):7385-90 | Population / region |
| Sun | Effects of early enteral nutrition on T helper lymphocytes of surgical septic patients: A retrospective observational study. | Medicine (Baltimore). 2017 Aug; 96(32): e7702 | Population / region |
| Sunden-Cullberg | Fever in the Emergency Department Predicts Survival of Patients With Severe Sepsis and Septic Shock Admitted to the ICU. | Crit Care Med. 2017 Apr;45(4):591-599. | No outcome of interest |
| Suzuki | Prospective intervention study with a microarray-based, multiplexed, automated molecular diagnosis instrument (Verigene system) for the rapid diagnosis of bloodstream infections, and its impact on the clinical outcomes. | J Infect Chemother. 2015 Dec;21(12):849-56 | Population / region |
| Svoboda | Terlipressin in the treatment of late phase catecholamine-resistant septic shock. | Hepatogastroenterology. 2012 Jun;59(116):1043-7. | No outcome of interest |
| Szakmany | Sepsis Prevalence and Outcome on the General Wards and Emergency Departments in Wales: Results of a Multi-Centre, Observational, Point Prevalence Study. | PLoS One. 2016 Dec 1;11(12):e0167230 | No outcome of interest |
| Tagami, T | Intravenous immunoglobulin and mortality in pneumonia patients with septic shock: an observational nationwide study. | Clin Infect Dis. 2015 Aug 1;61(3):385-92 | No outcome of interest |
| Taito, S | Rehabilitation for patients with sepsis: A systematic review and meta-analysis. | PLoS One. 2018 Jul 26;13(7):e0201292 | No outcome of interest |
| Tekwani, K | A prospective observational study of the effect of etomidate on septic patient mortality and length of stay. | Acad Emerg Med. 2009 Jan;16(1):11-4 | No outcome of interest |
| Terayama, T | Polymyxin B Hemoperfusion for Sepsis and Septic Shock: A Systematic Review and Meta-Analysis. | Surg Infect (Larchmt). 2017 Apr;18(3):225-233 | Population / region |
| The National Heart, Lung, and Blood Institute ARDS Clinical Trials Network | Rosuvastatin for Sepsis-Associated Acute Respiratory Distress Syndrome | N Engl J Med. 2014 Jun 5;370(23):2191-200 | No outcome of interest |
| Thiele, H | Intraaortic balloon support for myocardial infarction with cardiogenic shock. | N Engl J Med 2012; 367:1287-1296 | No outcome of interest |
| Thomas, G | Statin therapy in critically-ill patients with severe sepsis: a review and meta-analysis of randomized clinical trials. | Minerva Anestesiol. 2015 Aug;81(8):921-30 | No outcome of interest |
| Timsit, J | Empirical Micafungin Treatment and Survival Without Invasive Fungal Infection in Adults With ICU-Acquired Sepsis, Candida Colonization, and Multiple Organ Failure: The EMPIRICUS Randomized Clinical Trial. | JAMA. 2016 Oct 18;316(15):1555-1564 | No outcome of interest |
| Tol, M | A preliminary investigation into adrenal responsiveness and outcomes in patients with cardiogenic shock after acute myocardial infarction. | J Crit Care. 2014 Jun;29(3):470.e1-6 | No outcome of interest |
| Tolsma, V | Sepsis severe or septic shock: outcome according to immune status and immunodeficiency profile. | Chest. 2014 Nov;146(5):1205-1213 | No outcome of interest |
| Torgersen, C | Comparing two different arginine vasopressin doses in advanced vasodilatory shock: a randomized, controlled, open-label trial. | Intensive Care Med. 2010 Jan;36(1):57-65 | No outcome of interest |
| Tralhao, A | Impact of statins in outcomes of septic patients: a systematic review. | Postgrad Med. 2014 Nov;126(7):45-58 | No outcome of interest |
| Trentzsch, H | Female sex protects from organ failure and sepsis after major trauma haemorrhage. | Injury. 2014 Oct;45 Suppl 3:S20-8 | No outcome of interest |
| Trof, R | Volume-limited versus pressure-limited hemodynamic management in septic and nonseptic shock. | Crit Care Med. 2012 Apr;40(4):1177-85 | No outcome of interest |
| Truwit | Rosuvastatin for sepsis-associated acute respiratory distress syndrome. | N Engl J Med. 2014 Jun 5;370(23):2191-200 | No outcome of interest |
| Tsaganos, T | Clarithromycin Leads to Long-Term Survival and Cost Benefit in Ventilator-Associated Pneumonia and Sepsis. | Antimicrob Agents Chemother. 2016 May 23;60(6):3640-6 | No outcome of interest |
| Tsai, Ming-Hung | Acute renal failure in cirrhotic patients with severe sepsis: value of urinary interleukin-18. | Journal of gastroenterology and hepatology | No outcome of interest |
| Tu, Yuexing | Urinary netrin-1 and KIM-1 as early biomarkers for septic acute kidney injury. | Ren Fail. 2014 Nov;36(10):1559-63 | Population / region |
| Tulloch, Luis | Epidemiology and Microbiology of Sepsis Syndromes in a University-Affiliated Urban Teaching Hospital and Level-1 Trauma and Burn Center. | J Intensive Care Med. 2017 May;32(4):264-272 | No outcome of interest |
| Umemura, Y | Efficacy and safety of anticoagulant therapy in three specific populations with sepsis: a meta-analysis of randomized controlled trials. | J Thromb Haemost. 2016 Nov;14(11):2310-2311 | No outcome of interest |
| Unsal, Abdulkadir | Clinical outcomes and mortality in peritoneal dialysis patients: a 10-year retrospective analysis in a single center. | Clin Nephrol. 2013 Oct;80(4):270-9 | No outcome of interest |
| Vales, Jordi | Device-associated infection rates in Adult Intensive Care Units in Catalonia: VINCat Program findings. | Enfermedades infecciosas y microbiologia clinica | No outcome of interest |
| Vallabhajosyula, Saraschandra | Impact of New-Onset Left Ventricular Dysfunction on Outcomes in Mechanically Ventilated Patients With Severe Sepsis and Septic Shock. | J Intensive Care Med. 2018 Dec;33(12):680-686 | No outcome of interest |
| van Vught, Lonneke | Comparative Analysis of the Host Response to Community-acquired and Hospital-acquired Pneumonia in Critically Ill Patients. | Am J Respir Crit Care Med. 2016 Dec 1;194(11):1366-1374 | Indication |
| van Vught, Lonneke | Association of Gender With Outcome and Host Response in Critically Ill Sepsis Patients. | Crit Care Med. 2017 Nov;45(11):1854-1862 | No original data or duplicate to other study |
| van Vught, Lonneke | The Host Response in Patients with Sepsis Developing Intensive Care Unit-acquired Secondary Infections. | Am J Respir Crit Care Med. 2017 Aug 15;196(4):458-470 | No outcome of interest |
| van Vught, Lonneke | Admission Hyperglycemia in Critically Ill Sepsis Patients: Association With Outcome and Host Response. | Crit Care Med. 2016 Jul;44(7):1338-46 | No outcome of interest |
| Van Wert, Ryan | High-dose renal replacement therapy for acute kidney injury: Systematic review and meta-analysis. | Crit Care Med. 2010 May;38(5):1360-9 | Indication |
| van Zanten, Arthur | Guideline bundles adherence and mortality in severe sepsis and septic shock. | Crit Care Med. 2014 Aug;42(8):1890-8 | No outcome of interest |
| Venot, Marion | Acute Kidney Injury in Severe Sepsis and Septic Shock in Patients with and without Diabetes Mellitus: A Multicenter Study. | PLoS One. 2015 May 28;10(5):e0127411 | No outcome of interest |
| Viaggi, Bruno | Mid regional pro-adrenomedullin for the prediction of organ failure in infection. Results from a single centre study. | PLoS One. 2018 Aug 13;13(8):e0201491 | No outcome of interest |
| Vincent, Jean Louis | The value of blood lactate kinetics in critically ill patients: A systematic review | Crit Care. 2016 Aug 13;20(1):257 | No original data or duplicate to other study |
| Vincent, Jean Louis | Acute kidney injury, acute lung injury and septic shock: how does mortality compare? | Contrib Nephrol. 2011;174:71-77 | No original data or duplicate to other study |
| Volbeda, M | Glucocorticosteroids for sepsis: systematic review with meta-analysis and trial sequential analysis. | Intensive Care Med. 2015; 41(7): 1220–1234. | No outcome of interest |
| Vos, Fidel J | Metastatic infectious disease and clinical outcome in Staphylococcus aureus and Streptococcus species bacteremia. | Medicine (Baltimore). 2012 Mar;91(2):86-94 | Indication |
| Wacharasint | Normal-range blood lactate concentration in septic shock is prognostic and predictive | Shock (Augusta, Ga.), 38(1), 4–10 | No outcome of interest |
| Waechter | Interaction between fluids and vasoactive agents on mortality in septic shock: a multicenter, observational study | Critical Care Medicine, 42(10), 2158–2168 | No outcome of interest |
| Walkey | Atrial fibrillation among Medicare beneficiaries hospitalized with sepsis: incidence and risk factors. | Am Heart J. 2013 Jun;165(6):949-955.e3 | No outcome of interest |
| Wan | Effect of statin therapy on mortality from infection and sepsis: a meta-analysis of randomized and observational studies. | Crit Care. 2014 Apr 11;18(2):R7 | Indication |
| Wang | Angiotensin-converting enzyme inhibitor usage and acute kidney injury: a secondary analysis of RENAL study outcomes | Nephrology (Carlton, Vic.), 19(10), 617–622 | No outcome of interest |
| Wang | Diagnostic and prognostic value of neutrophil gelatinase-associated lipocalin, matrix metalloproteinase-9, and tissue inhibitor of matrix metalloproteinases-1 for sepsis in the Emergency Department: an observational study. | Crit Care. 2014 16;18(6):634 | No outcome of interest |
| Wang | Low-dose hydrocortisone therapy attenuates septic shock in adult patients but does not reduce 28-day mortality: a meta-analysis of randomized controlled trials | Anesthesia and Analgesia, 118(2), 346–357 | No outcome of interest |
| Wang | The Efficacy and Immunomodulatory Effects of Ulinastatin and Thymosin alpha1 for Sepsis: A Systematic Review and Meta-Analysis. | Biomed Res Int. 2016;2016:9508493 | No outcome of interest |
| Wang | Early initiation of renal replacement treatment in patients with acute kidney injury: A systematic review and meta-analysis. | Medicine (Baltimore). 2016 Nov;95(46):e5434. | Indication |
| Wang | The role of increased body mass index in outcomes of sepsis: a systematic review and meta-analysis. | BMC Anesthesiol. 2017 Aug 31;17(1):118 | No outcome of interest |
| Wang | Central Venous Pressure Dropped Early is Associated with Organ Function and Prognosis in Septic Shock Patients: A Retrospective Observational Study | Shock (Augusta, Ga.), 44(5), 426–430 | No outcome of interest |
| Wang | Effect of small-dose levosimendan on mortality rates and organ functions in Chinese elderly patients with sepsis. | Clin Interv Aging. 2017 May 29;12:917-92 | Population / region |
| Wang | Risk factors for mortality in patients with septic acute kidney injury in intensive care units in Beijing, China: a multicenter prospective observational study. | Biomed Res Int. 2014;2014:172620 | No outcome of interest |
| Wang | Contrasting treatment and outcomes of septic shock: presentation on hospital floors versus emergency department. | Chinese Medical Journal, 123(24), 3550–3553 | No outcome of interest |
| Warmerdam | The association between systolic blood pressure and in-hospital mortality in older emergency department patients who are hospitalised with a suspected infection | Emerg Med J. 2018 Oct;35(10):619-622 | No outcome of interest |
| Weingart | Comparison of Coagulation Parameters, Anticoagulation, and Need for Transfusion in Patients on Interventional Lung Assist or Veno-Venous Extracorporeal Membrane Oxygenation | Artificial Organs, 39(9), 765–773 | No outcome of interest |
| Weston | The efficacy of daptomycin versus vancomycin for methicillin-resistant Staphylococcus aureus bloodstream infection in patients with impaired renal function. | Clin Infect Dis. 2014 Jun;58(11):1533-9 | Indication |
| Whitson | Feasibility, Utility, and Safety of Midodrine During Recovery Phase From Septic Shock. | Chest. 2016 Jun;149(6):1380-3 | No outcome of interest |
| Whittaker | Epidemiology and outcomes in patients with severe sepsis admitted to the hospital wards | Journal of Critical Care, 30(1), 78–84. | No outcome of interest |
| Wiewel | Chronic antiplatelet therapy is not associated with alterations in the presentation, outcome, or host response biomarkers during sepsis: a propensity-matched analysis. | Intensive Care Med. 2016 Mar;42(3):352-360 | No outcome of interest |
| Wiewel | Risk factors, host response and outcome of hypothermic sepsis | Critical Care (London, England), 20(1), 328 | No outcome of interest |
| Williams | Characteristics, treatment and outcomes for all emergency department patients fulfilling criteria for septic shock: a prospective observational study | Official Journal of the European Society for Emergency Medicine, 25(2), 97–104 | No outcome of interest |
| Williams | Systemic Inflammatory Response Syndrome, Quick Sequential Organ Function Assessment, and Organ Dysfunction: Insights From a Prospective Database of ED Patients With Infection | Chest, 151(3), 586–59 | No outcome of interest |
| Winters | Long-term mortality and quality of life in sepsis: a systematic review. | Crit Care Med. 2010 May;38(5):1276-83 | No outcome of interest |
| Wira | Meta-analysis of protocolized goal-directed hemodynamic optimization for the management of severe sepsis and septic shock in the Emergency Department. | West J Emerg Med. 2014 Feb;15(1):51-9 | No outcome of interest |
| Wong | Comparison of outcomes in patients with methicillin-susceptible Staphylococcus aureus (MSSA) bacteremia who are treated with β-lactam vs vancomycin empiric therapy: a retrospective cohort study | BMC Infect Dis. 2016 May 23;16:224 | No outcome of interest |
| Wong | Comparative effectiveness of beta-lactam versus vancomycin empiric therapy in patients with methicillin-susceptible Staphylococcus aureus (MSSA) bacteremia. | Ann Clin Microbiol Antimicrob. 2016 Apr 26;15:27 | Indication |
| Wong | The influence of intensive care unit-acquired central line-associated bloodstream infection on in-hospital mortality: A single-center risk-adjusted analysis | American Journal of Infection Control, 44(5), 587–592 | No outcome of interest |
| Woth | Platelet aggregation in severe sepsis | Journal of Thrombosis and Thrombolysis, 31(1), 6–12 | No outcome of interest |
| Wu | The efficacy of thymosin alpha 1 for severe sepsis (ETASS): a multicenter, single-blind, randomized and controlled trial. | Crit Care. 2013 Jan 17;17(1):R8 | Population / region |
| Xu | Comparison of the effects of albumin and crystalloid on mortality in adult patients with severe sepsis and septic shock: a meta-analysis of randomized clinical trials | Crit Care. 2014 Dec 15;18(6):702 | No outcome of interest |
| Xu | The Effect of Early Goal-Directed Therapy on Outcome in Adult Severe Sepsis and Septic Shock Patients: A Meta-Analysis of Randomized Clinical Trials | Anesth Analg. 2016 Aug;123(2):371-8 | No outcome of interest |
| Xu | Corticosteroid administration is associated with improved outcome of patients presenting high inflammatory cytokine levels during septic shock | Pediatric Blood & Cancer, 61(12), 2243–2248 | No outcome of interest |
| Yalci | Evaluation of Infectious Complications in the First Year After Kidney Transplantation | Transplantation Proceedings, 47(5), 1429–1432 | No outcome of interest |
| Yamakawa | Benefit profile of anticoagulant therapy in sepsis: a nationwide multicentre registry in Japan. | Crit Care. 2016 Jul 29;20(1):229. | No outcome of interest |
| Yamakawa | Recombinant human soluble thrombomodulin in severe sepsis: a systematic review and meta-analysis. | J Thromb Haemost. 2015 Apr;13(4):508-19. | No outcome of interest |
| Yamamichi | Comparison between non-septic and septic cases in stone-related obstructive acute pyelonephritis and risk factors for septic shock: A multi-center retrospective study | Official Journal of the Japan Society of  Chemotherapy, 24(11), 902–906 | No outcome of interest |
| Yang | The declined levels of inflammatory cytokines related with weaning rate during period of septic patients using ventilators | The Clinical Respiratory Journal, 12(2), 772–778 | No outcome of interest |
| Yang | Prognosis of alcohol-associated lactic acidosis in critically ill patients: an 8-year study. | Sci Rep. 2016 Oct 17;6:35368. | No outcome of interest |
| Yang | Body temperature control in patients with refractory septic shock: too much may be harmful | Chinese Medical Journal, 126(10), 1809–1813 | No outcome of interest |
| Yang | Respiratory Dysfunction in Patients With Sepsis: Protective Effect of Diabetes Mellitus | Am J Crit Care. 2011 Mar;20(2):e41-7 | No outcome of interest |
| Yang | Positive blood culture is not associated with increased mortality in patients with sepsis-induced acute respiratory distress syndrome | Respirology. 2013 Nov;18(8):1210-6 | Population / region |
| Yaroustovsky | Preliminary report regarding the use of selective sorbents in complex cardiac surgery patients with extensive sepsis and prolonged intensive care stay | Blood Purification, 28(3), 227–233 | No outcome of interest |
| Yende | Long-Term Quality of Life Among Survivors of Severe Sepsis: Analyses of Two International Trials. | Crit Care Med. 2016 Aug;44(8):1461-7 | No outcome of interest |
| Yergens | Assessing the association between occupancy and outcome in critically Ill hospitalized patients with sepsis. | BMC Emerg Med. 2015 Oct 19;15:31 | No outcome of interest |
| Yilmaz | Mortality predictors of Staphylococcus aureus bacteremia: a prospective multicenter study | Ann Clin Microbiol Antimicrob. 2016 Feb 9;15:7 | No outcome of interest |
| Ylipalosaari | Comparison of the epidemiology, risk factors, outcome and degree of organ failures of patients with candidemia acquired before or during ICU treatment. | Crit Care. 2012 Dec 12;16(2):R62 | No outcome of interest |
| Yoon | Tree-structured survival analysis of patients with Pseudomonas aeruginosa bacteremia: A multicenter observational cohort study | Diagnostic Microbiology and Infectious Disease, 87(2), 180–187 | No outcome of interest |
| Yousef | The Predictive Prognostic Values of Serum TNF-𝛼 in Comparison to SOFA Score Monitoring in Critically Ill Patients | Biomed Res Int. 2013;2013:258029 | No outcome of interest |
| Yu | Effect of early goal-directed therapy on mortality in patients with severe sepsis or septic shock: a meta-analysis of randomised controlled trials | BMJ Open. 2016 Mar 1;6(3):e008330 | No outcome of interest |
| Yu | Global end-diastolic volume index vs CVP goal-directed fluid resuscitation for COPD patients with septic shock: a randomized controlled trial. | The American Journal of Emergency Medicine, 35(1), 101–105 | No outcome of interest |
| Yu | A prospective randomized trial using blood volume analysis in addition to pulmonary artery catheter, compared with pulmonary artery catheter alone, to guide shock resuscitation in critically ill surgical patients | Shock (Augusta, Ga.), 35(3), 220–228 | No outcome of interest |
| Yu | Comparing hydrocortisone and methylprednisolone in patients with septic shock. | Advances in Therapy, 26(7), 728–735 | No outcome of interest |
| Zahar | Outcomes in severe sepsis and patients with septic shock: pathogen species and infection sites are not associated with mortality | Critical Care Medicine, 39(8), 1886–1895 | No outcome of interest |
| Zamani | Survival benefits of dexmedetomidine used for sedating septic patients in intensive care setting: A systematic review | Journal of Critical Care, 32, 93–100 | No outcome of interest |
| Zamri | Comparison of complication outcomes in acute pancreatitis following ERCP and conservative management at UKM medical centre: a six years retrospective study | La Clinica Terapeutica, 163(6), 467–471 | No outcome of interest |
| Zangrillo | A meta-analysis of complications and mortality of extracorporeal membrane oxygenation | Critical Care Medicine, 15(3), 172–178. | No outcome of interest |
| Zangrillo | Levosimendan reduces mortality in patients with severe sepsis and septic shock: A meta-analysis of randomized trials | Journal of Critical Care, 30(5), 908–913 | No outcome of interest |
| Zarychanski | The efficacy and safety of heparin in patients with sepsis: a systematic review and metaanalysis | Critical Care Medicine, 43(3), 511–518. | No outcome of interest |
| Zasowski | Time Is of the Essence: The Impact of Delayed Antibiotic Therapy on Patient Outcomes in Hospital-Onset Enterococcal Bloodstream Infections | Clin Infect Dis. 2016 May 15;62(10):1242-1250 | No outcome of interest |
| Zechmeister | Continuation of Statin Therapy and Vasopressor Use in Septic Shock | The Annals of Pharmacotherapy, 49(7), 790–795 | No outcome of interest |
| Zeng | The impact of previous hospitalization in the preceding 90 days on the outcome in critically ill patients with gram-negative bloodstream infection | Diagnostic Microbiology and Infectious Disease, 80(2), 136–140. | No outcome of interest |
| Zhang | Time to Appropriate Antibiotic Therapy Is an Independent Determinant of Postinfection ICU and Hospital Lengths of Stay in Patients With Sepsis | Critical Care Medicine, 43(10), 2133–2140 | No outcome of interest |
| Zhang | Early goal-directed therapy in the management of severe sepsis or septic shock in adults: a meta-analysis of randomized controlled trials | BMC Med. 2015 Apr 3;13:71 | No outcome of interest |
| Zhang | Effect of the intensity of continuous renal replacement therapy in patients with sepsis and acute kidney injury: a single-center randomized clinical trial | Nephrol Dial Transplant. 2012 Mar;27(3):967-73 | Population / region |
| Zhang | APACHE III Outcome Prediction in Patients Admitted to the Intensive Care Unit with Sepsis Associated Acute Lung Injury | PLoS One. 2015 Sep 30;10(9):e0139374 | No outcome of interest |
| Zhang | Antipyretic therapy in critically ill patients with established sepsis: a trial sequential analysis. | PLoS One. 2015 Feb 24;10(2):e0117279 | No outcome of interest |
| Zhang | Effectiveness of treatment based on PiCCO parameters in critically ill patients with septic shock and/or acute respiratory distress syndrome: a randomized controlled trial | Intensive Care Med. 2015 Mar;41(3):444-51 | No outcome of interest |
| Zhang | Intensive- vs less-intensive-dose continuous renal replacement therapy for the intensive care unit-related acute kidney injury: a meta-analysis and systematic review | J Crit Care. 2010 Dec;25(4):595-600 | No outcome of interest |
| Zhang | Protective effect of Xuebijing injection on myocardial injury in patients with sepsis: a randomized clinical trial. | J Tradit Chin Med. 2016 Dec;36(6):706-10 | Population / region |
| Zhao | Prognostic Value of Plasma Tight-Junction Proteins for Sepsis in Emergency Department: an Observational Study | Shock. 2016 Mar;45(3):326-32 | No outcome of interest |
| Zhao | Pharmacokinetic and Pharmacodynamic Efficacies of Continuous versus Intermittent Administration of Meropenem in Patients with Severe Sepsis and Septic Shock: A Prospective Randomized Pilot Study | Chin Med J (Engl). 2017 May 20;130(10):1139-1145 | No outcome of interest |
| Zhao | Predictive value of the complement system for sepsis-induced disseminated intravascular coagulation in septic patients in emergency department | Journal of Critical Care, 30(2), 290–295 | Population / region |
| Zhao | Evaluation of the Mortality in Emergency Department Sepsis score combined with procalcitonin in septic patients | Am J Emerg Med. 2013 Jul;31(7):1086-91 | Population / region |
| Zhao | Effects of Hydrocortisone on Regulating Inflammation, Hemodynamic Stability, and Preventing Shock in Severe Sepsis Patients | Med Sci Monit. 2018 May 30;24:3612-3619 | No outcome of interest |
| Zheng | Xuebijing combined with ulinastation benefits patients with sepsis: A meta-analysis | The American Journal of Emergency Medicine, 36(3), 480–487 | No outcome of interest |
| Zhong | Colloid solutions for fluid resuscitation in patients with sepsis: systematic review of randomized controlled trials | The Journal of Emergency Medicine, 45(4), 485–495 | No outcome of interest |
| Zhou | Blood Purification and Mortality in Sepsis: A Meta-analysis of Randomized Trials | Crit Care Med. 2013 Sep;41(9):2209-20 | No outcome of interest |
| Zhou | Persistent hyperlactatemia-high central venous-arterial carbon dioxide to arterial-venous oxygen content ratio is associated with poor outcomes in early resuscitation of septic shock | The American Journal of Emergency Medicine, 35(8), 1136–1141 | No outcome of interest |
| Zhou | Use of stepwise lactate kinetics-oriented hemodynamic therapy could improve the clinical outcomes of patients with sepsisassociated hyperlactatemia | Crit Care. 2017 Feb 16;21(1):33 | No outcome of interest |
| Zhu | Varying Presentations and Outcomes of Septic Shock: Should Septic Shock Be Stratified? | Am Surg. 2017 Nov 1;83(11):1235-1240 | No outcome of interest |
| Zielske | Acute and long-term dysphagia in critically ill patients with severe sepsis: results of a prospective controlled observational study. | Eur Arch Otorhinolaryngol. 2014 Nov;271(11):3085-93 | No outcome of interest |
| Zilberberg | Cost-effectiveness of micafungin as an alternative to fluconazole empiric treatment of suspected ICU-acquired candidemia among patients with sepsis: a model simulation. | Crit Care. 2009;13(3):R94. doi: 10.1186/cc7924. | No outcome of interest |
| Zimmerman | Adjunctive use of physostigmine salicylate (Anticholium(R)) in perioperative sepsis and septic shock: study protocol for a randomized, double-blind, placebo-controlled, monocentric trial (Anticholium(R) per Se). | Trials. 2017 Nov 10;18(1):530 | No original data or duplicate to other study |
| Zitt | Iron Supplementation and Mortality in Incident Dialysis Patients: An Observational Study | PLoS One. 2014 Dec 2;9(12):e114144 | Indication |
| Zitterman | Circulating 25-Hydroxyvitamin D and 1,25-Dihydroxyvitamin D Concentrations and Postoperative Infections in Cardiac Surgical Patients: The CALCITOP-Study. | PLoS One. 2016 Jun 29;11(6):e0158532 | No outcome of interest |
| Zou | Comparison of the effects of albumin and crystalloid on mortality among patients with septic shock: systematic review with meta-analysis and trial sequential analysis. | Sao Paulo Med J. 2018 Sep-Oct;136(5):421-432. | No outcome of interest |
| Zu | Therapeutic Value of Blood Purification and Prognostic Utilities of Early Serum Procalcitonin, C Reactive Protein, and Brain Natriuretic Peptide Levels in Severely Burned Patients with Sepsis. | Cell Biochem Biophys. 2015 May;72(1):259-63 | No outcome of interest |
| Zusman | Venous thromboembolism prophylaxis with anticoagulation in septic patients: a prospective cohort study. | QJM. 2015 Mar;108(3):197-204 | Population / region |
